# Characteristics and varieties of gases enclathrated in natural gas hydrates retrieved at Lake Baikal

**DOI:** 10.1038/s41598-023-31669-7

**Published:** 2023-03-17

**Authors:** Akihiro Hachikubo, Hirotsugu Minami, Hirotoshi Sakagami, Satoshi Yamashita, Alexey Krylov, Gennadiy Kalmychkov, Jeffrey Poort, Marc De Batist, Andrey Manakov, Oleg Khlystov

**Affiliations:** 1 Kitami Institute of Technology, 165 Koen-Cho, Kitami, 090-8507 Japan; 2 Limnological Institute, SB RAS, 3 Ulan-Batorskaya St., Irkutsk, 664033 Russia; 3 Institute of Earth Sciences, St. Petersburg State University, 7-9, Universitetskaya Nab., St. Petersburg, 199034 Russia; 4 VNIIOkeangeologia, Anglyisky Prospect 1, St. Petersburg, 190121 Russia; 5 Vinogradov Institute of Geochemistry, SB RAS, 1-a Favorsky St, Irkutsk, 664033 Russia; 6 Sorbonne Université, CNRS, Institut des Sciences de la Terre de Paris, ISTeP, 4 Place Jussieu, 75252 Paris, France; 7 Renard Centre of Marine Geology, Ghent University, Krijgslaan 281 s8, 9000 Ghent, Belgium; 8 Nikolaev Institute of Inorganic Chemistry, SB RAS, 3 Acad. Lavrentiev Ave, Novosibirsk, 630090 Russia

**Keywords:** Biogeochemistry, Limnology

## Abstract

Molecular and stable isotope compositions of hydrate-bound gases collected from 59 hydrate-bearing sites between 2005 to 2019 in the southern and central sub-basins of Lake Baikal are reported. The δ^2^H of the hydrate-bound methane is distributed between − 310‰ and − 270‰, approximately 120‰ lower than its value in the marine environment, due to the difference in δ^2^H between the lake water and seawater. Hydrate-bound gases originate from microbial (primary and secondary), thermogenic, and mixed gas sources. Gas hydrates with microbial ethane (δ^13^C: − 60‰, δ^2^H: between − 310‰ and − 250‰) were retrieved at approximately one-third of the total sites, and their stable isotope compositions were lower than those of thermogenic ethane (δ^13^C: − 25‰, δ^2^H: − 210‰). The low δ^2^H of ethane, which has rarely been reported, suggests for the first time that lake water with low hydrogen isotope ratios affects the formation process of microbial ethane as well as methane. Structure II hydrates containing enclathrated methane and ethane were collected from eight sites. In thermogenic gas, hydrocarbons heavier than ethane are biodegraded, resulting in a unique system of mixed methane-ethane gases. The decomposition and recrystallization of the hydrates that enclathrate methane and ethane resulted in the formation of structure II hydrates due to the enrichment of ethane.

## Introduction

Natural gas hydrate enclathrated hydrocarbons occur in marine/lacustrine sediments and sub-permafrost layers. Natural gas hydrates are not only a potential future energy resource^1–4^ but also a large reservoir of methane (C_1_), the second most important greenhouse gas^5,6^. Gas hydrates are crystalline compounds in which guest molecules are enclathrated in water cages constructed by hydrogen bonds. The differences in the crystal structure caused by the combination of cages of different sizes affect their physicochemical properties, such as hydration number, cage occupancies, and dissociation heat. Three crystallographic structures of natural gas hydrates have been identified as: cubic structure I (sI), cubic structure II (sII), and hexagonal structure H (sH)^7,8^. sI comprises dodecahedral (5^12^) and tetrakaidecahedral (5^12^6^2^) cages, whereas sII is composed of 5^12^ and hexakaidecahedral (5^12^6^4^) cages. sH has a large icosahedral (5^12^6^8^) cage in its unit cell and can encapsulate larger guest molecules.

Natural hydrocarbon gases can be primarily classified as biogenic or abiogenic gases. Biogenic gases are further divided into two types: microbial and thermogenic. Microbial gases mainly consist of C_1_ produced under anaerobic conditions by methanogens classified as archaea, and two pathways are known: CO_2_ reduction and methyl-type fermentation. In contrast, thermogenic gases are produced by the thermal cracking of organic matter in deep sediment layers and contain heavier hydrocarbons, such as ethane (C_2_), propane (C_3_), and butane (C_4_). Additionally, secondary microbial gases produced by microbes during biodegradation of petroleum appear more abundant than primary microbial gases^9^. To estimate the origin of natural hydrocarbon gases, diagrams have been proposed and refined using the molecular ratio of heavier hydrocarbons to C_1_ and their carbon isotope ratios^10–13^. Recently, a web-based tool was developed to determine the origin of natural gas using machine-learning models^14^.

C_1_ is the main component of guest gases in natural gas hydrates found in marine/lacustrine sediments worldwide. It mostly comprises microbial C_1_ from CO_2_ reduction, with very few other hydrocarbon components, such as C_2_ and C_3_, which generally comprise less than 0.1%^15–19^. Pure C_1_ hydrates form sI; thus, the majority of natural gas hydrates found to date belong to sI^15^.

Cubic structure II (sII) has been discovered in the Caspian Sea^20^, Gulf of Mexico^21^, and Sea of Marmara^22^. Also natural sH hydrates have been retrieved: (1) in the Gulf of Mexico as suggested by the molecular composition of hydrate-bound gases^23^, and (2) off Vancouver Island, Canada, they were directly confirmed by powder X-ray diffraction and ^13^C nuclear magnetic resonance spectroscopy^8^. Because the cages in sII and sH are larger than those in sI, they can enclathrate C_3_, C_4_, and even larger hydrocarbon molecules. For example, the guest gas in the Gulf of Mexico^24^ contained nearly 15% C_3_, whereas samples from the Sea of Marmara^22^ contained approximately 19% C_3_ and 10% isobutane (*i*-C_4_, 2-methylpropane). sH samples from offshore Vancouver Island^8^ enclathrated methylcyclopentane and methylcyclohexane. Though predominantly methane, thermogenic gases contain large amounts of C_2_, C_3_, and C_4_, and these heavier hydrocarbons decrease the equilibrium pressure of the mixed-gas hydrate and stabilize the system of mixed-gas hydrates^7^. Molecular fractionation also occurs during gas hydrate crystallization and affects the molecular composition of hydrate-bound gases^25,26^. Therefore, differences in the molecular composition of natural gas due to their origin determine the diversity of the crystallographic structure of natural gas hydrates.

Lake Baikal is divided into three sub-basins: southern, central, and northern. The southern and central sub-basins are separated by the Selenga Delta accommodation zone, and the central and northern sub-basins are separated by the Olkhon Island and the Academician Ridge. During the 1997 Baikal Drilling Project (BDP), gas hydrates were first discovered in sediments at depths of 121 m and 161 m below the lake bed in the southern sub-basin^27^. Subsequently, researchers have reported that near-surface gas hydrates exist at depths of up to several meters below the lake bottom in the southern and central sub-basins^28–35^.

In particular, the sII discovered in the Kukuy Canyon (central sub-basin) was a C_1_ and C_2_ mixed gas hydrate that contained thermogenic gas but few C_3_, C_4_, or larger hydrocarbon molecules^31^. Laboratory experiments have shown that C_1_ and C_2_, both of which are sI-formers, form sII in a mixed system^36,37^. Because Lake Baikal is the only known place where sII of C_1_ + C_2_ systems exist, further studies are needed to determine how such a system occurs.

**Figure 1:**
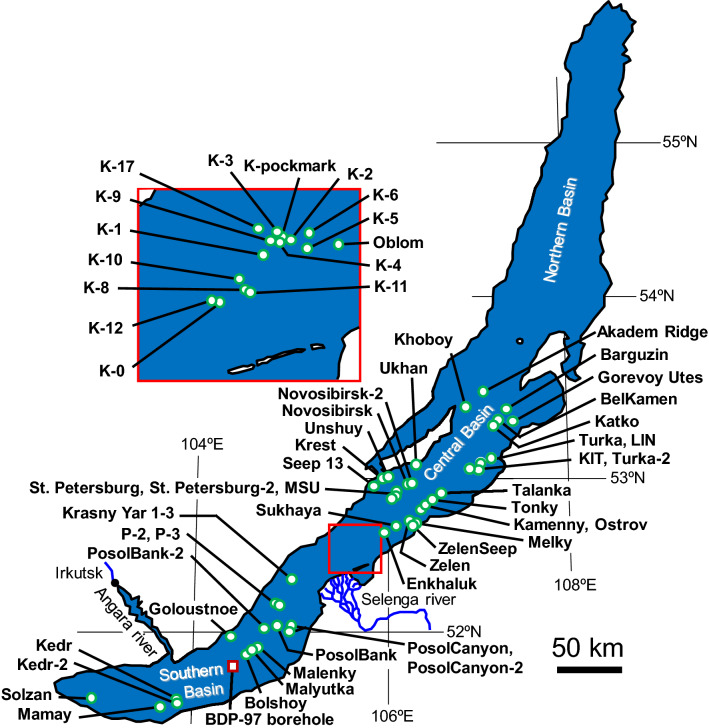
Locations of gas hydrate-bearing sites in Lake Baikal.

The authors continuously investigated near-surface gas hydrates in Lake Baikal within the framework of the multi-phase gas hydrate project (MHP) from 2009 to 2019. Of the 60 hydrate-bearing structures discovered by 2019 (Fig. 1), 48 sites were discovered by the MHP^38,39^. The water depths at sites where gas hydrates were recovered range from 396 m (Goloustnoe) to 1508 m (Novosibirsk-2)^38^. In this study, all gas data obtained so far in the MHP, along with previously reported hydrate-bound gas data^33,40–42^, were analyzed to understand the origin of hydrate-bound gases and their diversity in Lake Baikal.

## Results

**Figure 2:**
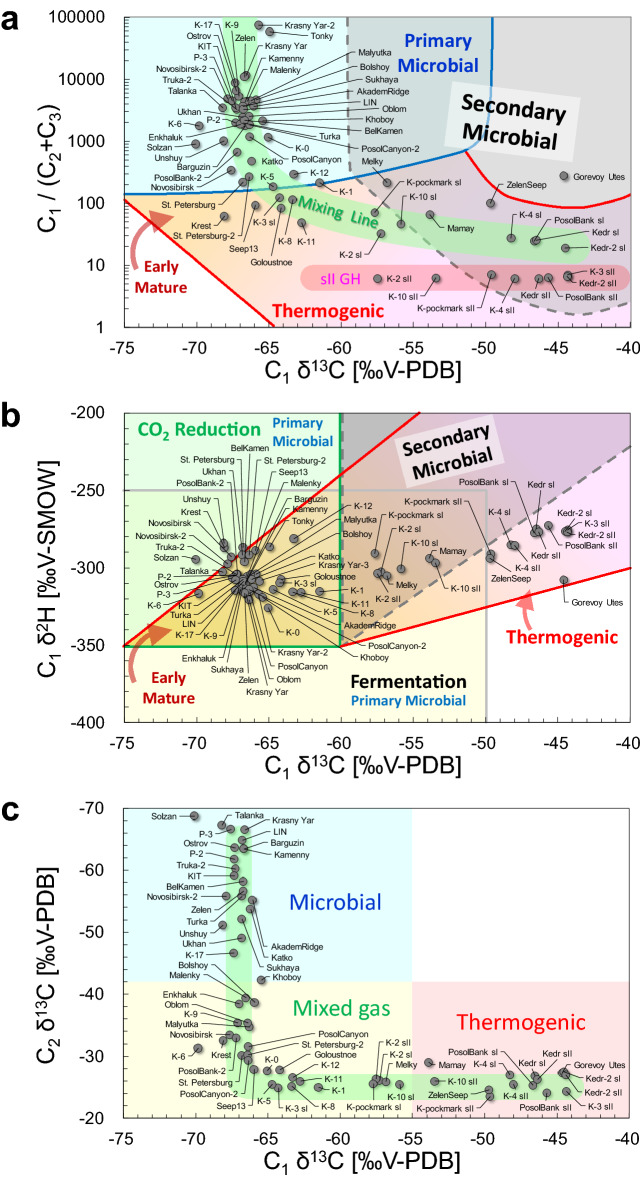
Empirical diagrams of hydrate-bound gases. (**a**) C_1_/(C_2_ + C_3_) plotted against C_1_ δ^13^C, based on the classification of Milkov and Etiope^13^; (**b**) C_1_ δ^2^H plotted against C_1_ δ^13^C, based on the classification of Milkov and Etiope^13^; and (**c**) C_2_ δ^13^C plotted against C_1_ δ^13^C, based on the classification of Milkov^15^. The data for Malenky, Bolshoy, Malyutka, P-2, K-0, K-2 and Goloustnoe are sourced partly from Hachikubo et al.^33^. The data for Kedr and Kedr-2 are sourced partly from Hachikubo et al.^41^.

**Figure 3:**
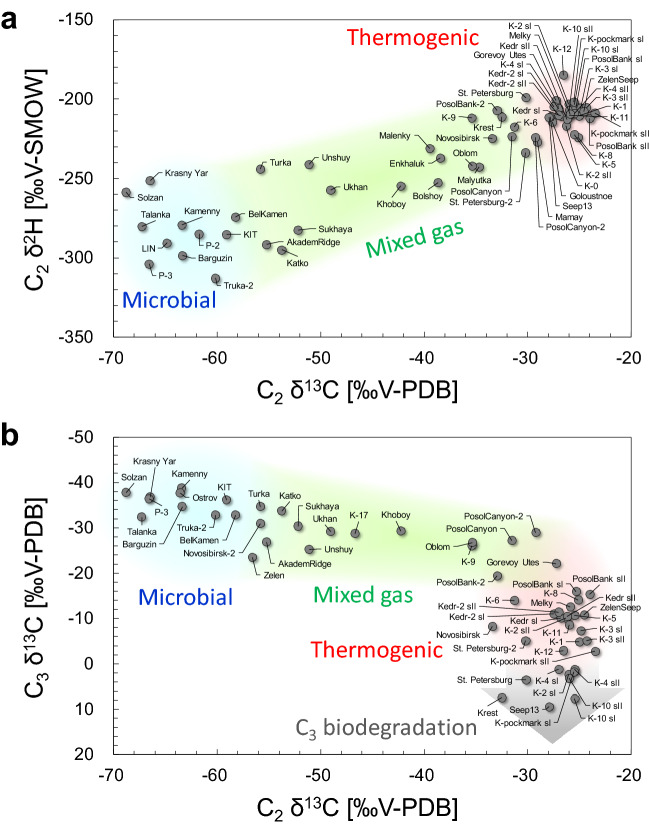
Stable isotope compositions of C_2_ and C_3_. (**a**) Relation between C_2_ δ^2^H and C_2_ δ^13^C. (**b**) Relation between C_3_ δ^13^C and C_2_ δ^13^C. The data for Malenky, Bolshoy, Malyutka, P-2, K-0, K-2 and Goloustnoe are sourced partly from Hachikubo et al.^33^. The data for Kedr and Kedr-2 are sourced partly from Hachikubo et al.^41^.

The gas analysis results are shown in Figs. 2 and 3. These data were obtained from 668 hydrate-bound gas samples collected at each site and the median value for each site was plotted. Of these samples, 479 were analyzed in this study, while the others, i.e. 93 and 96, respectively, were analyzed in previous works^33,41^. For the eight sites where double structure (sI and sII) gas hydrates were obtained, inferred crystallographic structures are specified on the graph. The mixing lines between the microbial and thermogenic origins in Fig. 2a,c are plotted for C_1_ δ^13^C, C_2_ δ^13^C, and C_1_/(C_2_ + C_3_), with assumed end members, respectively, of − 67‰, − 65‰, and 100,000 for microbial origin, and − 44‰, − 25‰, and 20 for thermogenic origin.

Figure 2a shows the relationship between C_1_ δ^13^C and C_1_/(C_2_ + C_3_) plotted in the empirical diagram^13^. More than 20 of the 60 total sites have C_1_ δ^13^C between − 68‰ and − 65‰ and C_1_/(C_2_ + C_3_) concentrated around 1000–5000, which means that microbial gas is enclathrated in more than one-third of the hydrate-bearing sites in Lake Baikal. However, along the mixing line from the microbial to thermogenic regions, C_1_ δ^13^C increases with a decrease in C_1_/(C_2_ + C_3_), passing through the mixed region of microbial and thermogenic gases to thermogenic gas (e.g., K-4, PosolBank, Kedr, and Kedr-2). For the eight sII hydrate data points, C_1_/(C_2_ + C_3_) is nearly constant at 6–7. Furthermore, C_1_ δ^13^C seems independent of the crystallographic structure at the same sites but differs considerably in K-3 and K-pockmark. This is because the hydrate-bearing sediment cores are different, even at the same site, indicating that the characteristics of the hydrate-bound gas can change markedly with slight differences in location. Gorevoy Utes^43,44^ is one of the two oil seep sites and plots in the field of secondary microbial gas (Fig. 2a)^9^. Another point, ZelenSeep, also plots near the Gorevoy Utes. Most of the data plotted for the thermogenic origin overlap with the field of secondary microbial gas.

Figure 2b shows the relationship between C_1_ δ^13^C and C_1_ δ^2^H, which is also plotted in an empirical diagram^13^. The isotopic fractionation of C_1_ between the gas and hydrate phases is negligible when considering gas origins using a diagram^45^. C_1_ δ^13^C tends to increase with C_1_ δ^2^H. In a diagram by Whiticar^12^, hydrate-bound C_1_ in Lake Baikal is interpreted to be of microbial origin via methyl-type fermentation^31–33,46^. However, the latest diagram by Milkov and Etiope^13^ shows that most of the C_1_ δ^13^C values below − 60‰ overlap completely with the microbial origin via CO_2_ reduction and may possibly be of an early mature origin. Therefore, it is difficult to determine the origin of C_1_ in Fig. 2b.

Figure 2c shows the relationship between C_1_ δ^13^C and C_2_ δ^13^C plotted on an empirical diagram^15^. All data are distributed in an "L" shape along the mixing line. This is because the composition of C_2_ in the microbial gas is so small that even a small amount of thermogenic gas with a large C_2_ composition can considerably affect and increase C_2_ δ^13^C during the mixing of microbial and thermogenic gases. As shown in Fig. 2c, hydrate-bound gases of natural gas hydrates in Lake Baikal can be classified into three categories: (1) both C_1_ and C_2_ of microbial origin, (2) C_1_ primarily of microbial origin, C_2_ primarily of thermogenic origin, and (3) both C_1_ and C_2_ of thermogenic origin.

Figure 3a shows that the relationship between C_2_ δ^13^C and C_2_ δ^2^H is similar to that in Fig. 2b for C_1_. This diagram was first proposed by Hachikubo et al.^33^, who showed that both C_2_ δ^13^C and C_2_ δ^2^H in P-2 were relatively lower than those at other sites (approximately − 60‰ and − 285‰, respectively). The amount of data increased over the next 10 years of investigation, and it is mostly concentrated in the area of the diagram interpreted as thermogenic C_2_ (C_2_ δ^13^C: − 25‰, C_2_ δ^2^H: − 210‰). In contrast, a group of C_2_ δ^13^C of approximately − 60‰, which corresponds to microbial C_2_, has low C_2_ δ^2^H ranging from − 310‰ to − 250‰. Therefore, the C_2_ δ^2^H of microbial gas is apparently lower than that of the thermogenic gas.

Figure 3b shows the relationship between C_2_ δ^13^C and C_3_ δ^13^C. The thermogenic gases are concentrated at approximately − 25‰ and − 10‰ for C_2_ δ^13^C and C_3_ δ^13^C, respectively. Based on the empirical diagram^15^, the value of C_2_ δ^13^C at approximately − 42‰ is interpreted as an approximate boundary between microbial and thermogenic C_2_. C_3_ δ^13^C is above 0‰ in K-2 (sI), K-4 (sI and sII), K-10 (sI and sII), K-pockmark (sI), St. Petersburg, Krest, and Seep13 in the field of thermogenic C_2_. In contrast, C_3_ δ^13^C is generally below − 30‰ in the field of microbial C_2_.

## Discussion

### Structure II hydrates in Lake Baikal

The sII hydrates from the Gulf of Mexico and Sea of Marmara contained large amounts (> 10%) of C_3_ and C_4_, whereas in Lake Baikal the ratio of C_3_ and C_4_ in the hydrate-bound gas is < 0.5% in the sII hydrates (Table S1). Therefore, C_1_ and C_2_ mixed-gas systems are responsible for the appearance of sII in Lake Baikal. Pure C_1_ and C_2_ hydrates each form sI, but in C_1_ and C_2_ mixed-gas systems, sII appears at certain mixing ratios^36,37^. In Lake Baikal, sII hydrates, in which the hydrate-bound gas was 85% C_1_ and 15% C_2_, were retrieved at Kukuy K-2 in 2005^31–33,46^. Generally, the sII hydrate is adjacent to the sI hydrate with 1–4% C_2_, forming a "double structure". Current understanding of the process is that the formation processes of double-structure gas hydrates in Lake Baikal have been discussed extensively^34,46–48^. The formation of sI hydrates blocked the gas supply channel, causing dissolution of sI hydrates and secondary formation of sII hydrates by enrichment of C_2_ from the dissociated gas^34,47^. A detailed investigation of the sII hydrates at Kedr and Kedr-2 revealed that besides C_2_ also C_3_, *i*-C_4_, n-butane (*n*-C_4_), and neopentane (*neo*-C_5_, 2,2-dimethylpropane) are enriched in the crystals^41^. The sII hydrates are identified at eight sites: Kukuy K-2, K-3, K-4, K-10, and K-pockmark in the central sub-basin and PosolBank, Kedr, and Kedr-2 in the southern sub-basin. The C_1_/(C_2_ + C_3_) ratios of these hydrate-bound gases are concentrated at approximately 6–7 (Fig. 2a), and the contribution of C_3_ is negligible compared to that of C_2_ (Table S1). The homogeneous gas composition of sII hydrates over a wide area of Lake Baikal can be explained by the decomposition processes of C_1_ and C_2_ mixed gas hydrates and the concentration of C_2_^41,49^.

The C_1_ and C_2_ mixed-gas system that gives rise to sII hydrates in Lake Baikal is established by the relative depletion of heavier hydrocarbons, such as C_3_ and C_4_ (Table S1). For example, in Kedr and Kedr-2, the maximum compositions of C_3_, *i*-C_4_, *n*-C_4_, and *neo*-C_5_ in the hydrate-bound gas were 0.3% and 270, 25, and 540 ppm, respectively^41^. These low compositions are likely due to biodegradation. In the area of Fig. 3b where C_2_ is of thermogenic origin (δ^13^C >  − 42‰), C_3_ δ^13^C is widely distributed from − 20 to + 10‰, suggesting the effect of biodegradation. For example, C_3_ is selectively affected by microbial alteration and exhibits anomalous C_3_ δ^13^C^50^. C_1_-rich dry gas, large C_1_ δ^13^C (− 55‰ to − 35‰), and large CO_2_ δ^13^C (> + 2‰) have been proposed as characteristics of secondary microbial C_1_^9^. The molecular and stable isotope compositions of CO_2_ in the hydrate-bound gas are unknown; however, the CO_2_ δ^13^C in the sediment gas around the hydrate crystals reaches + 20‰ (Kedr) and + 30‰ (Kedr-2)^41^, indicating the generation of secondary microbial C_1_. With two exceptions (Kukuy K-2 and K-10), the hydrate-bound gases of the sII crystals plot in the area of secondary microbial gases in Fig. 2a. Thus, at most sites in Lake Baikal, where thermogenic gas is supplied from a deeper sediment layer, hydrocarbons heavier than C_3_ are selectively and microbially degraded, resulting in the appearance of C_1_ and C_2_ mixed gas, further dissociation of sI hydrate, and the formation of sII hydrate adjacent to sI.

### Hydrogen isotope compositions of methane in hydrate-bound gas

The C_1_ δ^2^H of hydrate-bound gas in marine sediments is generally concentrated between approximately − 200‰ to − 190‰ for microbial gas and is greater for thermogenic gas, reaching approximately − 140‰ for gas hydrates retrieved offshore of Vancouver Island and Costa Rica^15^. The distribution of C_1_ δ^2^H of the thermogenic gas ranges from − 300‰ to − 100‰^11^ and from − 350‰ to − 100‰^13^. In addition, C_1_ δ^2^H tends to increase with C_1_ δ^13^C^11,12^. The factors that determine the C_1_ δ^2^H of thermogenic gas have not yet been clarified; however, it can be assumed that hydrogen isotope exchange occurs between C_1_ and environmental water. Based on the effect of temperature on the hydrogen isotope fractionation between C_1_ and hydrogen, and between hydrogen and water^51^, the hydrogen isotope fractionation between C_1_ and water can be expected to be smaller at higher temperatures. If the thermogenic gas produced by the decomposition of organic matter exchanges isotopically with environmental water during decomposition, the C_1_ δ^2^H of thermogenic gas in the deep sediment layers becomes greater than that of microbial gas produced in shallower sediment layers.

The C_1_ δ^2^H of hydrate-bound gas in Lake Baikal is generally concentrated around − 310‰ for microbial gas and increases to − 280‰ to − 270‰ for gases of thermogenic origin (Fig. 2b). The difference in C_1_ δ^2^H between the seawater and freshwater environments is approximately + 120‰. The δ^2^H of lake bottom water is reported to be − 123 ± 2‰^52^, whereas the δ^2^H of seawater is approximately 0‰ as a standard hydrogen stable isotope. Accordingly, the difference in C_1_ δ^2^H between them can be attributed to the difference in the δ^2^H of the environmental water.

Two processes are involved in the formation of microbial C_1_: CO_2_ reduction and methyl-type fermentation. Previously, it was thought that the hydrogen in C_1_ is derived from environmental water via CO_2_ reduction, whereas a certain percentage of hydrogen is derived from the original organic matter via methyl-type fermentation^12,53^. Based on this, a diagram^12,53^ was proposed that could discriminate between CO_2_ reduction and methyl-type fermentation by C_1_ δ^2^H. It was considered a useful method for evaluating the origin of C_1_. However, it has been suggested that, even for C_1_ produced via methyl-type fermentation, the hydrogen in C_1_ is exchanged with water in the environment^54,55^. Therefore, C_1_ δ^2^H strongly reflects the information of environmental water, and the C_1_ δ^2^H of natural gas hydrates in Lake Baikal (freshwater environment) is interpreted to be distinct from natural gas hydrates from seawater due to the difference in the δ^2^H of water (0‰ for seawater compared to − 123‰ for lake water).

In Fig. 2b, the group with C_1_ δ^2^H in the range of − 298‰ to − 281‰ and C_1_ δ^13^C in the range of − 70‰ to − 63‰ is distinct from the other microbial plots. Except for Belkamen, Tonky, K-12, and Solzan, eight of these sites belong to the Gydratny and Olkhon faults in the central sub-basin. Although the details are unknown, it is possible that the origin of the water involved in methanogenesis in these fault systems differs from those at other sites. Gorevoy Utes is an oil seep site^43,44^, with C_1_ δ^13^C of − 45‰ and C_1_ δ^2^H of − 308‰, and is plotted farther away from the other areas (Fig. 2b). It is outside the thermogenic area with a C_1_/(C_2_ + C_3_) ratio of 274 (Fig. 2a) and small compositions of C_2_ and C_3_, indicating the effect of secondary microbial gas. Anaerobic biodegradation of heavier hydrocarbons likely results in this gas composition^44^. However, the very low C_1_ δ^2^H of the Gorevoy Utes cannot be explained at this stage. ZelenSeep^42^ is also an oil seep site, and its characteristics for hydrate-bound gas are similar to those of the Gorevoy Utes (Fig. 2a,b).

### Microbial ethane and propane

Although information on ethanogens is still scarce, ethane with a small δ^13^C (< − 40‰) suggests an in situ microbial origin^56^. Experiments on biologically generated hydrocarbons in anaerobic sediments showed lower C_2_ δ^13^C values, ranging from − 55‰ to − 35‰^57^. The method of C_2_ formation by ethanogens is not yet understood; however, a process involving the reduction of acetic acid has been proposed^58^. Ethanogens are considered less competitive than methanogens^59^, therefore C_2_ compositions are very low compared to C_1_ compositions. In this study, the sites in the area of microbial C_2_ in Fig. 2c corresponded to sites where C_1_/(C_2_ + C_3_) was generally above 1000 (Fig. 2a).

There are several reports of microbial C_2_ in hydrate-bound gases in other marine areas. Charlou et al.^60^ analyzed gas hydrates collected in the Congo-Angola Basin and reported a C_2_ δ^13^C of − 61.4‰ for hydrate-bound gas. Milkov et al.^61^ reported stable isotope compositions of hydrate-bound gas collected during the Ocean drilling Program (ODP) Leg 204 at the Hydrate Ridge offshore Oregon and found C_2_ δ^13^C in the range between − 50‰ and − 30‰. Sassen and Curiale^16^ reported a C_2_ δ^13^C value of − 52.6‰ in hydrate-bound gas collected from the Makassar Strait in Indonesia, which they attributed to microbial C_2_. Lorenson and Collett^62^ analyzed the void gas and gas in pressure cores obtained from gas hydrate accumulation areas in the Bay of Bengal and reported a C_2_ δ^13^C of − 64‰ to − 52‰. Microbiological experimental work with Lake Baikal sediments showed the possibility of producing C_2_ as well as C_1_ by microbes^63^. Therefore, there are many cases in which microbial C_2_ produced in situ in shallow sediment layers is enclathrated into hydrate crystals. Figure 2c shows that there are 21 sites where gas hydrates enclathrate microbial C_2_, which represents approximately one-third of the total 60 hydrate-bearing sites in Lake Baikal.

Little is known regarding the hydrogen isotope ratios of microbial C_2_. Figure 3a presents valuable information on C_2_ δ^2^H in hydrate-bound gases, following a previous study^33^. Stable isotope compositions of thermogenic C_2_ concentrate with C_2_ δ^13^C and C_2_ δ^2^H values of approximately by − 25‰ and − 210‰, respectively, and C_2_ δ^2^H decreases with C_2_ δ^13^C. Microbial C_2_ is widely distributed, with C_2_ δ^13^C ranging from − 70‰ to − 60‰ and C_2_ δ^2^H from − 310‰ to − 250‰. This trend is similar to the relationship between C_1_ δ^13^C and C_1_ δ^2^H, suggesting that lake water with low hydrogen isotope ratios is also involved in the formation of microbial C_2_.

Although little information is available on microbial C_3_, a mechanism has been proposed by Hinrichs et al. for its formation from acetate and hydrogen^58^, in which it has been noted that C_3_ δ^13^C was greater than C_2_ δ^13^C, and Fig. 3b satisfies this relationship. In the area of microbial C_2_ where C_2_ δ^13^C is below − 42‰, C_3_ δ^13^C is also relatively low, ranging from − 40‰ to − 30‰, indicating that microbial C_3_ is more depleted in^13^C than thermogenic C_3_.

The reason for the presence of C_3_ in the samples of hydrate-bound gas of sI, even if only a small percentage, is still unclear. Due to its molecular size, C_3_ cannot be enclathrated in the 5^12^6^2^ cages of sI, but it can be enclathrated in the 5^12^6^4^ cages of sII. Therefore, it is possible that C_3_ adsorbs onto sediment particles and/or gas hydrate crystals and can be encaged if a small amount of sII crystals are present.

### Origin of hydrate-bound gas in Lake Baikal

Based on the characteristics of the molecular and isotopic compositions of hydrate-bound gases, the origin of the hydrate-bound gases in Lake Baikal is discussed from a geological perspective. The hydrate-bound gases in Lake Baikal can be classified into three main categories:

1. Thermogenic gas derived from crude oil, in some cases accompanied by secondary microbial gas;
2. Thermogenic gas rising up from deep sedimentary layers through faults, mixed with microbial gas in shallow layers;
3. Microbial gas formed in shallow sedimentary layers.

Each category can be associated to specific geological and geographical environments of Lake Baikal. Geological factors that affect gas hydrate formation in the central sub-basin are large amounts of deltaic deposits submitted to significant heating, the presence/absence of major faults, and oil seeping into the subsurface sediments. Oil seepage at the lake floor has been reported at two sites in the central sub-basin: Gorevoy Utes^43,44^ and ZelenSeep^42^. The hydrate-bound gases in these locations are of thermogenic origin, although there are signs of anaerobic biodegradation^44^ and of the influence of secondary microbial gas. The Selenga Delta is located in the southern part of the central sub-basin. It has the thickest lake sediment in Lake Baikal, of approximately 9 km, with abundant organic matter and it experiences frequent earthquakes. Hydrate-bound gases in the Kukuy Canyon (K-0 to K-17, Fig. 1) consist of thermogenic gases supplied from deeper sedimentary layers mixed with microbial gases in shallow layers at various mixing ratios. As mentioned previously, the C_1_ δ^2^H is greater at Seep 13, Krest, Unshuy, Uhkhan, Novosibirsk, Novosibirsk-2, St. Petersburg, and St. Petersburg-2 in the central sub-basin than at other sites (Fig. 2b). The Olkhon and Gydratny faults in the central sub-basin extend all the way up from the basement^64^, in a region where the sediment thickness reaches 7.5 km^65^. Thus, water with a greater δ^2^H than lake water is supplied from deeper sedimentary layers through the faults, possibly affecting the values of C_1_ δ^2^H at these sites.

In contrast, on the southeastern slope of the central sub-basin, with a few exceptions, microbial gases are predominant at most sites from Enkhaluk to Barguzin. No major faults are present in these sites. Based on the formation mechanism of mud volcanoes, such as on Academician Ridge^66^, hydrate-bound gases are microbially produced in relatively shallow layers, that is, from the lake bottom to a depth of 300–400 m, where the maximum temperature is approximately 40 °C.

The sedimentary layers are sufficiently thick, and major faults exist near Goloustnoe, PosolBank, PosolBank-2, PosolCanyon, and PosolCanyon-2 in the southern sub-basin, and the characteristics of the hydrate-bound gas are similar to those of the Kukuy Canyon. Faults are also adjacent to Malenky, Bolshoy, and Malyutka^28,29^. Although the gases at these sites are considered microbial gases^33^, the stable isotope ratios of C_2_ indicate that they are slightly contaminated by thermogenic gases (Figs. 2b and 3a). There is also the influence of coal deposits at Kedr, Kedr-2, and Mamay^67^, and gas hydrate enclathrates thermogenic gas accompanied by secondary microbial gas^41^.

Despite the existence of faults, Unshuy, Ukhan, and Novosibirsk-2 in the central sub-basin show small C_2_ δ^13^C values, indicating microbial gas. P-2, P-3, and Krasny Yar in the southern sub-basin are located in the periphery of the Selenga Delta, and their hydrate-bound gases are expected to be similar to those of the Kukuy Canyon in the central sub-basin; however, they are microbial gases. Further studies are required to explain these discrepancies. In conclusion, microbial gases (C_1_, C_2_, and C_3_) exist everywhere in the shallow sediments of Lake Baikal, and a mixing of small amount of thermogenic gas eliminates any trace of microbial C_2_. These gases form gas hydrates in subsurface sediments and display a rich diversity of gas compositions and crystallographic structures.

## Materials and methods

In the multi-phase gas hydrate project during 2009–2019, 11 cruises (VER09-03, VER10-03, VER11-01, VER12-03, VER13-03, VER14-03, VER15-03, VER16-03, VER17-03, VER18-03, and VER19-03) were conducted, and natural gas hydrates were sampled at 52 sites (Fig. 1). Detailed information on these sites is described in previous reports^35,38,39^. In this study, 668 hydrate-bound gas data points were organized according to sampling site. Hydrate-bound sediment cores were obtained using a gravity corer onboard R/V *G. Yu. Vereshchagin*. Gas hydrate samples in the sediments were collected rapidly and the hydrate-bound gases were stored in glass vials (5 mL) with butyl septum stoppers. From 2005 to 2013, gas hydrate crystals were placed in syringes (50 mL) and connected to a vial with a needle for a hydrate-bound gas sampling method^33^; however, after 2014, a water displacement method was used^41^. To avoid microbial alteration, 0.3 mL of a preservative (50 wt% aqueous solution of benzalkonium chloride) was introduced into the vials using a syringe.

The details of the gas analysis are similar to those described in previous studies^33,41^. Gas chromatography (GC-14B for 2005–2011 and GC-2014 for 2012–2019, Shimadzu, Kyoto, Japan) was used to analyze the molecular composition of the hydrocarbons. Both instruments consisted of a glass-packed column (Shimadzu Sunpak-S; length 2 m, inner diameter 3 mm), a thermal conductivity detector, and a frame ionization detector. The detectors were connected in series. The analytical error estimated by multiple injections of the standard gases was < 1.2% for each gas component. As crystallographic analysis was not conducted in this study, the crystallographic structure of the gas hydrate was estimated by the C_2_ composition in hydrocarbons according to the method in the previous work^41^. For the stable isotope analysis of hydrocarbons, continuous-flow isotope-ratio mass spectrometry (DELTA plus XP, Thermo Finnigan, Waltham, MA, USA for 2005–2013 and DELTA V, Thermo Fisher Scientific, Waltham, MA, USA for 2014–2019) was used. In all cases, a gas chromatograph (TRACE GC Ultra, Thermo Finnigan/Thermo Fisher Scientific, Waltham, MA, USA) equipped with a Carboxen-1006PLOT capillary column (length 30 m, inner diameter 0.32 mm, film thickness 15 μm, Sigma-Aldrich, St. Louis, MO, USA) was connected to the mass spectrometer. The stable isotope compositions are reported as δ values (in ‰).

1 $$\updelta =\left(\frac{{R}_{sample}-{R}_{standard}}{{R}_{standard}}\right)\times 1000 [\permil ]$$

where R denotes the ^13^C/^12^C or ^2^H/^1^H ratio. δ^13^C and δ^2^H were given with reference to the V-PDB and V-SMOW standards, respectively, and were determined using NIST RM8544 (NBS19) for δ^13^C and NIST RM8561 (NGS3) for δ^2^H. The analytical precisions for hydrocarbon (C_1_–C_3_) δ^13^C and δ^2^H were 0.3‰ and 1‰, respectively.

## Supplementary Information

Supplementary Tables.

## Data Availability

All the gas data are reported in the Supplementary Information.
