# Supplementary material for: Characteristics and varieties of gases enclathrated in natural gas hydrates retrieved at Lake Baikal
Source: Sci Rep. 2023 Mar 17;13:4440. doi: 10.1038/s41598-023-31669-7 (PMC10023797; doi:10.1038/s41598-023-31669-7)
Supplement: Supplementary file 1 — Supplementary Tables. [file 41598_2023_31669_MOESM1_ESM.pdf]

Supplementary Information

Characteristics and varieties of gases enclathrated in natural gas hydrates retrieved at Lake Baikal

Akihiro Hachikubo[1], Hirotsugu Minami[1], Hirotoshi Sakagami[1], Satoshi Yamashita[1], Alexey Krylov[2,3,4], Gennadiy Kalmychkov[5†], Jeffrey Poort[6], Marc De Batist[7], Andrey Manakov[8], and Oleg Khlystov[2]

[1] *Kitami Institute of Technology, 165 Koen-cho, Kitami 090-8507, Japan*
[2] *Limnological Institute, SB RAS, 3 Ulan-Batorskaya St., Irkutsk 664033, Russia*
[3] *Institute of Earth Sciences, St. Petersburg State University, 7-9, Universitetskaya Nab., St. Petersburg 199034, Russia*
[4] *VNIIOkeangeologia, Anglyisky prospect 1, St. Petersburg 190121, Russia*
[5] *Vinogradov Institute of Geochemistry, SB RAS, 1-a Favorsky St., Irkutsk 664033, Russia*
[6] *Sorbonne Université, CNRS, Institut des Sciences de la Terre de Paris, ISTeP, 4 Place Jussieu, 75252 Paris, France*
[7] *Renard Centre of Marine Geology, Ghent University, Krijgslaan 281 s8, 9000 Ghent, Belgium*
[8] *Nikolaev Institute of Inorganic Chemistry, SB RAS, 3 Acad. Lavrentiev Ave., Novosibirsk 630090, Russia*

†Deceased November 2020

Corresponding author: Akihiro Hachikubo (hachi@mail.kitami-it.ac.jp)

Table S1. Molecular and stable isotope compositions of hydrate-bound hydrocarbons at Lake Baikal. The site number is referred to in Khlystov et al.[1,2]. a) The gas data are partly referred to in Hachikubo et al.[3] b) The gas data are partly referred to in Hachikubo et al.[4] This work does not include gas data of No.48 MSU. cmblf, centimeters below lake floor. n.d., not determined

Table S2. Raw data of molecular and stable isotope compositions of hydrate-bound hydrocarbons at Lake Baikal. The site number is referred to in Khlystov et al.[1,2]. a) The gas data are referred to in Hachikubo et al.[3] b) The gas data are referred to in Hachikubo et al.[4] Crystallographic structure of gas hydrate is estimated as structure I (sI), where ethane (C_2) composition was <10%; structure II (sII), where C_2 composition was >10%. This work does not include gas data of No.48 MSU. cmblf, centimeters below lake floor. n.d., not determined

References

1 Khlystov, O. M., Khabuev, A. V., Minami, H., Hachikubo, A. & Krylov, A. A. Gas hydrates in Lake Baikal. *Limnol. Freshwater Biol.* **1**, 66–70. https://doi.org/10.31951/2658-3518-2018-A-1-66 (2018).

2 Khlystov, O. M. *et al.* The position of gas hydrates in the sedimentary strata and in the geological structure of Lake Baikal. In: Mienert J., Berndt C., Tréhu A.M., Camerlenghi A., Liu C.-S. (eds) World Atlas of Submarine Gas Hydrates in Continental Margins, Springer, Cham., 465–471. https://doi.org/10.1007/978-3-030-81186-0_39 (2022).

3 Hachikubo, A. *et al.* Molecular and isotopic characteristics of gas hydrate-bound hydrocarbons in southern and central Lake Baikal. *Geo-Mar. Lett.* **30**, 321–329. https://doi.org/10.1007/s00367-010-0203-1 (2010).

4 Hachikubo, A. *et al.* Characteristics of hydrate-bound gas retrieved at the Kedr mud volcano (southern Lake Baikal). *Sci. Rep.* **10**, 14747. https://doi.org/10.1038/s41598-020-71410-2 (2020).

Table S1

No.	Site	Sample	Crystallographic Structure	CH_4 [%]	C_2H_6 [%]	C_3H_8 [%]	i-C_4H_{10} [%]	n-C_4H_{10} [%]	neo-C_5H_{12} [%]	i-C_5H_{12} [%]	n-C_5H_{12} [%]	$C_1/(C_2+C_3)$	CH_4 $\delta^{13}C$ [‰VPDB]	C_2H_6 $\delta^{13}C$ [‰VPDB]	C_3H_8 $\delta^{13}C$ [‰VPDB]	CH_4 δ^2H [‰VSMOW]	C_2H_6 δ^2H [‰VSMOW]
				Molecular Composition									Isotopic Composition				
1	Malenky[a)]	23	sI	99.9774	0.0223	0.0003						4425	-66.5	-39.5		-305.5	-231.1
2	Bolshoy[a)]	5	sI	99.9776	0.0215	0.0009						4649	-65.9	-38.7		-304.1	-252.6
3	Kukuy K-2[a)]	26	sI	97.0147	2.9748	0.0076	0.0014	0.0003	0.0005	0.0006		32	-57.3	-26.1	2.3	-302.9	-203.2
		11	sII	86.0503	13.8920	0.0262	0.0133	0.0011	0.0171			6	-57.5	-26.3	-10.1	-303.8	-216.7
4	Kukuy K-0[a)]	9	sI	99.9038	0.0962							1150	-65.1	-27.6		-325.8	-214.7
5	Goreoy Utes	13	sI	99.6005	0.3554	0.0079	0.0048	0.0040	0.0000	0.0229	0.0046	274	-44.6	-27.3	-22.0	-307.7	-200.9
6	Malyutka[a)]	3	sI	99.9778	0.0222							4497	-66.3	-34.7		-304.5	-242.6
7	Kukuy K-6	4	sI	99.9436	0.0561	0.0003						1773	-69.8	-31.3	-13.9	-316.7	-217.4
8	Peschanka P-2[a)]	16	sI	99.9495	0.0505	0.0000						1982	-67.3	-61.7		-304.9	-285.0
9	Goloustnoe[a)]	19	sI	98.8329	1.1654	0.0017						85	-64.2	-27.8		-307.3	-211.2
10	St. Petersburg	5	sI	99.5480	0.4493	0.0023	0.0003	0.0001	0.0001	0.0000		220	-66.8	-30.2	3.5	-287.0	-199.4
11	Kukuy K-10	6	sI	96.9098	3.0900	0.0002		0.0001				46	-55.9	-25.5	7.7	-300.7	-203.4
		1	sII	86.2232	13.7636	0.0038			0.0093			6	-53.5	-26.0	3.1	-296.9	-207.3
12	Kukuy K-1	4	sI	99.5338	0.4657	0.0005						213	-61.5	-25.0	-4.9	-315.3	-210.6
13	Novosibirsk	21	sI	99.7053	0.2943	0.0005						341	-67.6	-33.4	-8.2	-292.8	-224.9
14	Kukuy K-8	5	sI	99.1428	0.8570	0.0001						116	-63.3	-25.1	-14.1	-315.9	-224.2
15	Kukuy K-9	50	sI	99.9814	0.0184	0.0002						5369	-67.1	-35.4	-26.1	-313.1	-211.9
16	PosolBank	6	sI	96.0436	3.9494	0.0048	0.0010	0.0010	0.0002	0.0001		24	-46.7	-25.4	-15.9	-277.7	-205.7
		5	sII	86.3942	13.5603	0.0246	0.0060	0.0013	0.0136			6	-45.7	-24.0	-15.3	-273.0	-212.1
17	Kukuy K-4	9	sI	96.4414	3.5546	0.0040						27	-48.3	-27.0	1.1	-285.3	-206.3
		4	sII	85.9954	13.9952	0.0094						6	-48.0	-25.5	1.2	-285.5	-209.1
18	Kukuy K-11	8	sI	97.9690	2.0270	0.0040						48	-62.8	-26.0	-8.6	-316.3	-210.2
19	Kukuy K-12	5	sI	99.6605	0.3391	0.0004						294	-63.3	-26.6	-2.9	-281.3	-184.9
20	Seep13	9	sI	98.9343	1.0653	0.0003		0.0000				93	-65.9	-27.9	9.4	-289.0	-211.4
21	Krest	2	sI	98.4099	1.5897	0.0004						63	-68.1	-32.5	7.4	-287.3	-211.1
22	Kukuy K-3	16	sI	99.1988	0.7998	0.0014						124	-64.2	-24.9	-7.4	-309.8	-205.9
		2	sII	87.5885	11.8916	0.4386	0.0545	0.0167	0.0083	0.0018		7	-44.3	-24.3	-5.1	-275.5	-205.5
23	Krasny Yar	25	sI	99.9912	0.0087	0.0001						11375	-66.6	-66.5	-36.8	-317.2	-251.4
24	Kukuy K-pockmark	7	sI	98.5986	1.3909	0.0069	0.0017	0.0003	0.0003	0.0012		71	-57.7	-25.5	1.7	-291.0	-201.8
		5	sII	87.7288	12.1257	0.1048	0.0126	0.0021	0.0256	0.0004		7	-49.6	-23.5	-2.7	-291.3	-208.7
25	Ukhan	5	sI	99.9587	0.0408	0.0005						2419	-66.8	-49.0	-29.2	-291.8	-257.5
26	Unshuy	6	sI	99.9019	0.0980	0.0001						1018	-68.1	-51.1	-25.2	-284.1	-241.2
27	Krasny Yar-2	1	sI	99.9987	0.0013	0.0000						74185	-65.7			-317.1	
28	Krasny Yar-3	1	sI										-65.6			-308.7	
29	Kukuy K-5	15	sI	99.4617	0.5381	0.0001	0.0001	0.0000				185	-64.7	-25.5	-10.7	-314.2	-222.4
30	St. Petersburg-2	13	sI	99.6246	0.3547	0.0205	0.0001	0.0000				266	-66.4	-30.2	-5.1	-292.0	-233.8
31	Khoboy	18	sI	99.9533	0.0466	0.0001		0.0001				2152	-65.4	-42.3	-29.2	-319.2	-254.8

Table S1 (continue)				Molecular Composition									Isotopic Composition				
No.	Site	Sample	Crystallographic Structure	CH_4 [%]	C_2H_6 [%]	C_3H_8 [%]	i-C_4H_{10} [%]	n-C_4H_{10} [%]	neo-C_5H_{12} [%]	i-C_5H_{12} [%]	n-C_5H_{12} [%]	$C_1/(C_2+C_3)$	CH_4 $\delta^{13}C$ [‰VPDB]	C_2H_6 $\delta^{13}C$ [‰VPDB]	C_3H_8 $\delta^{13}C$ [‰VPDB]	CH_4 δ^2H [‰VSMOW]	C_2H_6 δ^2H [‰VSMOW]
32	AkademRidge	31	sI	99.9723	0.0271	0.0006	0.0001	0.0000				3618	-66.0	-55.2	-26.8	-312.9	-291.5
33	Barguzin	8	sI	99.9430	0.0567	0.0002	0.0000					1755	-66.7	-63.4	-34.6	-309.7	-298.4
34	Mamay	9	sI	98.4916	1.5082	0.0002						65	-53.9	-29.1		-293.8	-227.0
35	Kamenny	4	sI	99.9769	0.0228	0.0003	0.0000	0.0000				4341	-66.7	-63.4	-38.7	-310.4	-279.1
36	Tonky	2	sI	99.9983	0.0016	0.0001						57769	-64.9			-286.6	
37	Talanka	4	sI	99.9712	0.0287	0.0002						3469	-68.2	-67.3	-32.3	-302.7	-280.2
38	Novosibirsk-2	5	sI	99.9794	0.0203	0.0002		0.0000				4862	-67.9	-55.8	-30.9	-297.5	
39	Kedr [b)]	46	sI	96.2299	3.7645	0.0040	0.0003	0.0001	0.0012	0.0000		26	-46.5	-26.8	-10.2	-274.8	-210.4
		47	sII	85.9564	13.9862	0.0371	0.0031	0.0004	0.0167	0.0001		6	-46.3	-26.2	-10.4	-276.9	-212.8
40	PosolBank-2	18	sI	99.8485	0.1485	0.0029	0.0001	0.0001				660	-67.2	-33.0	-19.4	-304.2	-207.3
41	Ostrov	5	sI	99.9844	0.0154	0.0002	0.0000	0.0000				6408	-67.2	-63.6	-37.7	-304.9	
42	Turka	10	sI	99.9488	0.0510	0.0002		0.0000				1957	-66.8	-55.8	-34.6	-307.4	-244.2
43	Kedr-2 [b)]	3	sI	94.9870	4.9987	0.0112	0.0008	0.0003	0.0019	0.0001		19	-44.5	-27.5	-11.0	-276.4	-204.8
		9	sII	86.8662	13.1142	0.0120	0.0006	0.0001	0.0069			7	-44.4	-27.0	-11.4	-276.9	-210.4
44	Solzan	4	sI	99.8876	0.1122	0.0001		0.0000				897	-70.1	-68.8	-37.7	-294.5	-258.6
45	Oblom	3	sI	99.9605	0.0392	0.0002	0.0000	0.0000				2532	-66.3	-35.3	-26.7	-320.6	-242.1
46	Truka-2	3	sI	99.9681	0.0318	0.0001		0.0000				3239	-67.2	-60.1	-32.8	-304.6	-312.9
47	Peschanka P-3	2	sI	99.9753	0.0243	0.0003	0.0000	0.0000	0.0000			4457	-67.5	-66.6	-36.4	-306.4	-303.8
48	MSU	0															
49	Enkhaluk	2	sI	99.9451	0.0548	0.0000		0.0000				1854	-67.0	-38.5		-313.7	-237.2
50	Sukhaya	8	sI	99.9724	0.0274	0.0001	0.0000	0.0000				3633	-66.8	-52.1	-30.3	-310.8	-282.7
51	KIT (Kitami)	9	sI	99.9707	0.0287	0.0005	0.0000	0.0000				3423	-67.3	-59.1	-36.1	-307.6	-285.2
52	LIN	7	sI	99.9691	0.0306	0.0002	0.0000	0.0001				3250	-66.7	-64.8		-308.7	-290.6
53	PosolCanyon	9	sI	99.9157	0.0837	0.0005		0.0001				1187	-66.3	-31.5	-27.2	-315.5	-223.6
54	PosolCanyon-2	11	sI	99.9450	0.0540	0.0008	0.0002	0.0001				1825	-66.3	-29.3	-29.0	-314.0	-224.0
55	Zelen	5	sI	99.9907	0.0088	0.0005	0.0000	0.0000				10792	-66.7	-56.6	-23.5	-316.4	
56	ZelenSeep	4	sI	99.0086	0.9823	0.0087	0.0001	0.0001	0.0004			100	-49.7	-24.6	-10.8	-293.8	-207.9
57	Melky	6	sI	99.5361	0.4491	0.0124	0.0003	0.0001	0.0019	0.0000		216	-56.8	-25.9	-12.5	-305.1	-209.6
58	Katko	3	sI	99.7920	0.2079	0.0001	0.0000	0.0000				480	-66.2	-53.7	-33.7	-307.4	-294.7
59	BelKamen	4	sI	99.9502	0.0336	0.0122	0.0033	0.0004	0.0001	0.0002		2178	-66.7	-58.2	-32.8	-296.2	-274.4
60	Kukuy K-17	4	sI	99.9885	0.0113	0.0002	0.0000	0.0000				8732	-67.3	-46.7	-28.7	-314.8	

Table S2

No.	Site	Cruise	Core	Sample Depth [cmblf]	Molecular Composition CH$_4$ [%]	C$_2$H$_6$ [%]	C$_3$H$_8$ [%]	i-C$_4$H$_{10}$ [%]	n-C$_4$H$_{10}$ [%]	neo-C$_5$H$_{12}$ [%]	i-C$_5$H$_{12}$ [%]	n-C$_5$H$_{12}$ [%]	C$_1$/(C$_2$+C$_3$)	Isotopic Composition CH$_4$ δ^{13}C [‰VPDB]	C$_2$H$_6$ δ^{13}C [‰VPDB]	C$_3$H$_8$ δ^{13}C [‰VPDB]	CH$_4$ δ^2H [‰VSMOW]	C$_2$H$_6$ δ^2H [‰VSMOW]	Crystallographic Structure	note
1	Malenky	VER05-03	2005St3GC1	122	99.9736	0.0260	0.0004	n.d.	n.d.	n.d.	n.d.	n.d.	3784	-65.7	n.d.	n.d.	-306.3	n.d.	sI	[a]
1	Malenky	VER05-03	2005St3GC1	122	99.9817	0.0178	0.0005	n.d.	n.d.	n.d.	n.d.	n.d.	5463	-66.0	n.d.	n.d.	-306.8	n.d.	sI	[a]
1	Malenky	VER05-03	2005St3GC1	126	99.9713	0.0284	0.0003	n.d.	n.d.	n.d.	n.d.	n.d.	3485	-65.1	n.d.	n.d.	-308.4	n.d.	sI	[a]
1	Malenky	VER05-03	2005St3GC1	126	99.9722	0.0275	0.0003	n.d.	n.d.	n.d.	n.d.	n.d.	3599	-65.9	n.d.	n.d.	-307.6	n.d.	sI	[a]
1	Malenky	VER05-03	2005St3GC2	118	99.9781	0.0216	0.0004	n.d.	n.d.	n.d.	n.d.	n.d.	4559	-67.0	-36.8	n.d.	-306.9	n.d.	sI	[a]
1	Malenky	VER05-03	2005St3GC2	152	99.9833	0.0163	0.0004	n.d.	n.d.	n.d.	n.d.	n.d.	5990	-65.8	n.d.	n.d.	-301.3	n.d.	sI	[a]
1	Malenky	VER05-03	2005St3GC2	152	99.9858	0.0139	0.0003	n.d.	n.d.	n.d.	n.d.	n.d.	7028	-65.4	n.d.	n.d.	-305.5	n.d.	sI	[a]
1	Malenky	VER05-03	2005St3GC2	152	99.9889	0.0107	0.0004	n.d.	n.d.	n.d.	n.d.	n.d.	9030	-65.8	n.d.	n.d.	-307.7	n.d.	sI	[a]
1	Malenky	VER05-03	2005St3GC2	175	99.9774	0.0223	0.0003	n.d.	n.d.	n.d.	n.d.	n.d.	4425	-65.7	n.d.	n.d.	-303.6	n.d.	sI	[a]
1	Malenky	VER05-03	2005St3GC2	175	99.9775	0.0223	0.0003	n.d.	n.d.	n.d.	n.d.	n.d.	4437	-66.0	n.d.	n.d.	-304.5	n.d.	sI	[a]
1	Malenky	VER05-03	2005St3GC2	175	99.9788	0.0209	0.0003	n.d.	n.d.	n.d.	n.d.	n.d.	4712	-65.6	n.d.	n.d.	-307.5	n.d.	sI	[a]
1	Malenky	VER07-04	2007St1GC4	120	99.9625	0.0369	0.0006	n.d.	n.d.	n.d.	n.d.	n.d.	2668	-67.4	-37.5	n.d.	-303.0	n.d.	sI	[a]
1	Malenky	VER07-04	2007St1GC4	120	99.9854	0.0126	0.0020	n.d.	n.d.	n.d.	n.d.	n.d.	6868	-66.9	-40.6	n.d.	-302.4	-227.0	sI	[a]
1	Malenky	VER07-04	2007St1GC4	232	99.9690	0.0307	0.0002	n.d.	n.d.	n.d.	n.d.	n.d.	3228	-67.3	-39.1	n.d.	-304.2	-228.7	sI	[a]
1	Malenky	VER07-04	2007St1GC5	69	99.9778	0.0209	0.0013	n.d.	n.d.	n.d.	n.d.	n.d.	4495	-66.8	-37.4	n.d.	-300.4	-242.2	sI	[a]
1	Malenky	VER07-04	2007St1GC5	69	99.9856	0.0134	0.0010	n.d.	n.d.	n.d.	n.d.	n.d.	6951	-67.1	-40.3	n.d.	-302.0	-224.8	sI	[a]
1	Malenky	VER07-04	2007St1GC5	78	99.9708	0.0282	0.0010	n.d.	n.d.	n.d.	n.d.	n.d.	3427	-67.2	-40.8	n.d.	-303.2	-230.0	sI	[a]
1	Malenky	VER07-04	2007St1GC5	78	99.9837	0.0151	0.0011	n.d.	n.d.	n.d.	n.d.	n.d.	6142	-67.0	-37.9	n.d.	-303.0	-232.2	sI	[a]
1	Malenky	VER07-04	2007St1GC5	84	99.9732	0.0266	0.0002	n.d.	n.d.	n.d.	n.d.	n.d.	3730	-67.2	-39.5	n.d.	-307.7	n.d.	sI	[a]
1	Malenky	VER07-04	2007St1GC6	72	99.9548	0.0446	0.0006	n.d.	n.d.	n.d.	n.d.	n.d.	2211	-67.4	-39.7	n.d.	-306.7	-232.9	sI	[a]
1	Malenky	VER07-04	2007St1GC6	72	99.9708	0.0286	0.0007	n.d.	n.d.	n.d.	n.d.	n.d.	3418	-67.1	-39.5	n.d.	-304.2	-233.4	sI	[a]
1	Malenky	VER07-04	2007St1GC6	72	99.9677	0.0313	0.0009	n.d.	n.d.	n.d.	n.d.	n.d.	3097	-66.5	-39.1	n.d.	-305.5	n.d.	sI	[a]
1	Malenky	VER07-04	2007St1GC6	72	99.9580	0.0413	0.0007	n.d.	n.d.	n.d.	n.d.	n.d.	2378	-65.4	-40.2	n.d.	-308.0	n.d.	sI	[a]
2	Bolshoy	VER05-03	2005St1GC3	unknown	99.9776	0.0215	0.0009	n.d.	n.d.	n.d.	n.d.	n.d.	4454	-66.2	n.d.	n.d.	-300.4	n.d.	sI	[a]
2	Bolshoy	VER05-03	2005St1GC3	unknown	99.9765	0.0227	0.0008	n.d.	n.d.	n.d.	n.d.	n.d.	4252	-65.9	-38.7	n.d.	-305.4	-252.0	sI	[a]
2	Bolshoy	VER05-03	2005St1GC4	96	99.9770	0.0207	0.0023	n.d.	n.d.	n.d.	n.d.	n.d.	4340	-65.9	-40.0	n.d.	-304.1	-253.3	sI	[a]
2	Bolshoy	VER05-03	2005St1GC4	96	99.9673	0.0188	0.0139	n.d.	n.d.	n.d.	n.d.	n.d.	3053	-65.7	n.d.	n.d.	-303.8	n.d.	sI	[a]
2	Bolshoy	VER05-03	2005St1GC4	104	99.9811	0.0178	0.0010	n.d.	n.d.	n.d.	n.d.	n.d.	5297	n.d.	-35.3	n.d.	-307.2	n.d.	sI	[a]
3	Kukuy K-2	VER06-02	2006St2GC5	245	86.0328	13.9407	0.0265	n.d.	n.d.	n.d.	n.d.	n.d.	6	-57.3	-27.8	n.d.	-299.5	-215.9	sII	[a]
3	Kukuy K-2	VER06-02	2006St2GC5	245	85.5579	14.4421	n.d.	n.d.	n.d.	n.d.	n.d.	n.d.	6	-57.5	-25.9	n.d.	-303.8	-216.1	sII	[a]
3	Kukuy K-2	VER06-02	2006St2GC5	266	96.4160	3.5728	0.0112	n.d.	n.d.	n.d.	n.d.	n.d.	27	-57.5	-28.5	n.d.	-305.9	-205.8	sI	[a]
3	Kukuy K-2	VER06-02	2006St2GC5	266	97.7247	2.2753	n.d.	n.d.	n.d.	n.d.	n.d.	n.d.	43	-58.3	-26.0	n.d.	-308.5	-208.8	sI	[a]
3	Kukuy K-2	VER06-02	2006St2GC5	270	96.5940	3.3928	0.0131	n.d.	n.d.	n.d.	n.d.	n.d.	28	-57.9	-25.1	n.d.	-306.2	-202.9	sI	[a]
3	Kukuy K-2	VER06-02	2006St2GC7	253	85.8439	14.1343	0.0218	n.d.	n.d.	n.d.	n.d.	n.d.	6	-58.0	-27.3	n.d.	-302.8	-220.0	sII	[a]
3	Kukuy K-2	VER06-02	2006St2GC7	255	86.0309	13.9691	n.d.	n.d.	n.d.	n.d.	n.d.	n.d.	6	-57.6	-24.3	n.d.	-308.1	-219.4	sII	[a]
3	Kukuy K-2	VER06-02	2006St2GC7	267	96.8055	3.1902	0.0044	n.d.	n.d.	n.d.	n.d.	n.d.	30	-58.2	-28.9	n.d.	-302.6	-200.2	sI	[a]
3	Kukuy K-2	VER06-02	2006St2GC7	275	96.8330	3.1670	n.d.	n.d.	n.d.	n.d.	n.d.	n.d.	31	-57.3	-26.1	n.d.	-302.5	-211.5	sI	[a]
3	Kukuy K-2	VER06-02	2006St2GC7	280	96.9719	3.0231	0.0050	n.d.	n.d.	n.d.	n.d.	n.d.	32	-58.0	-25.9	n.d.	-303.1	-198.8	sI	[a]
3	Kukuy K-2	VER06-02	2006St2GC8	157	97.3511	2.6393	0.0095	n.d.	n.d.	n.d.	n.d.	n.d.	37	-57.0	-30.1	n.d.	-304.2	-199.8	sI	[a]
3	Kukuy K-2	VER06-02	2006St2GC22	275	85.9997	13.9792	0.0211	n.d.	n.d.	n.d.	n.d.	n.d.	6	-58.2	-26.4	n.d.	-303.5	-217.7	sII	[a]
3	Kukuy K-2	VER06-02	2006St2GC22	290	97.5564	2.4374	0.0062	n.d.	n.d.	n.d.	n.d.	n.d.	40	-57.4	-30.0	n.d.	-303.2	-196.1	sI	[a]
3	Kukuy K-2	VER06-02	2006St2GC22	300	97.4990	2.4939	0.0071	n.d.	n.d.	n.d.	n.d.	n.d.	39	-59.1	-29.7	n.d.	-305.9	-203.0	sI	[a]

Table S2 (continue)

No.	Site	Cruise	Core	Sample Depth [cmblf]	Molecular Composition CH_4 [%]	C_2H_6 [%]	C_3H_8 [%]	i-C_4H_{10} [%]	n-C_4H_{10} [%]	neo-C_5H_{12} [%]	i-C_5H_{12} [%]	n-C_5H_{12} [%]	$C_1/(C_2+C_3)$	Isotopic Composition CH_4 $\delta^{13}C$ [‰VPDB]	C_2H_6 $\delta^{13}C$ [‰VPDB]	C_3H_8 $\delta^{13}C$ [‰VPDB]	CH_4 δ^2H [‰VSMOW]	C_2H_6 δ^2H [‰VSMOW]	Crystallographic Structure	note
3	Kukuy K-2	VER06-02	2006St2GC23	172	86.2806	13.6912	0.0282	n.d.	n.d.	n.d.	n.d.	n.d.	6	-56.7	-28.0	n.d.	-304.1	-216.7	sII	a)
3	Kukuy K-2	VER06-02	2006St2GC23	184	95.5214	4.4561	0.0226	n.d.	n.d.	n.d.	n.d.	n.d.	21	-56.5	-24.5	n.d.	-303.4	-206.5	sI	a)
3	Kukuy K-2	VER06-02	2006St2GC23	187	98.4155	1.5636	0.0209	n.d.	n.d.	n.d.	n.d.	n.d.	62	-58.4	-29.7	n.d.	-301.0	-203.2	sI	a)
3	Kukuy K-2	VER06-02	2006St2GC25	334	85.7757	14.2003	0.0240	n.d.	n.d.	n.d.	n.d.	n.d.	6	-57.9	-25.5	n.d.	-304.7	-218.4	sII	a)
3	Kukuy K-2	VER06-02	2006St2GC30	93	86.3471	13.6301	0.0228	n.d.	n.d.	n.d.	n.d.	n.d.	6	-56.7	-25.1	n.d.	-303.6	-214.9	sII	a)
3	Kukuy K-2	VER06-02	2006St2GC30	200	98.0494	1.9478	0.0028	n.d.	n.d.	n.d.	n.d.	n.d.	50	-58.0	-29.2	n.d.	-301.2	-210.7	sI	a)
3	Kukuy K-2	VER06-02	2006St2GC30	240	98.2524	1.7427	0.0049	n.d.	n.d.	n.d.	n.d.	n.d.	56	-56.8	-23.2	n.d.	-305.1	-210.9	sI	a)
3	Kukuy K-2	VER06-02	2006St2GC32	113	86.1664	13.8155	0.0180	n.d.	n.d.	n.d.	n.d.	n.d.	6	-57.6	-27.4	n.d.	-307.3	-215.1	sII	a)
3	Kukuy K-2	VER13-03	2013St33GC1	295	86.6640	13.2498	0.0606	0.0104	0.0021	0.0126	0.0003	0.0001	7	-51.5	-26.3	-7.4	-285.1	-203.9	sII	
3	Kukuy K-2	VER13-03	2013St33GC1	300	90.6150	9.2653	0.0907	0.0156	0.0030	0.0100	0.0004	0.0000	10	-49.9	-26.2	-7.1	-279.8	-201.4	sI	
3	Kukuy K-2	VER13-03	2013St33GC1	305	92.1498	7.6753	0.1359	0.0233	0.0049	0.0097	0.0011	0.0000	12	-49.6	-26.0	-7.6	-279.4	-202.3	sI	
3	Kukuy K-2	VER13-03	2013St33GC1	313	95.8040	4.1917	0.0030	0.0008	0.0001	0.0003	0.0001	n.d.	23	-53.4	-25.7	3.3	-292.8	-199.2	sI	
3	Kukuy K-2	VER13-03	2013St35GC3	145	96.6762	3.3084	0.0097	0.0023	0.0004	0.0019	0.0011	n.d.	29	-56.8	-25.6	6.1	-297.3	-206.7	sI	
3	Kukuy K-2	VER13-03	2013St35GC3	150	97.5019	2.4888	0.0063	0.0018	0.0003	0.0003	0.0007	n.d.	39	-57.5	-25.8	4.4	-298.7	-202.9	sI	
3	Kukuy K-2	VER13-03	2013St35GC3	156	95.5119	4.4612	0.0181	0.0032	0.0005	0.0047	0.0004	n.d.	21	-57.0	-25.5	6.7	-298.6	-200.8	sI	
3	Kukuy K-2	VER13-03	2013St35GC3	165	97.0147	2.9748	0.0076	0.0014	0.0003	0.0005	0.0006	n.d.	33	-57.2	-25.6	5.2	-298.4	-203.1	sI	
3	Kukuy K-2	VER13-03	2013St35GC3	170	97.1367	2.8530	0.0074	0.0011	0.0003	0.0013	0.0000	n.d.	34	-58.0	-25.6	1.3	-300.6	-203.2	sI	
3	Kukuy K-2	VER14-03	2014St1GC5	227	98.6571	1.3390	0.0039	n.d.	n.d.	n.d.	n.d.	n.d.	73	-55.3	-26.5	n.d.	-298.5	n.d.	sI	
3	Kukuy K-2	VER14-03	2014St1GC5	233	97.9227	2.0565	0.0132	0.0068	0.0005	0.0003	n.d.	n.d.	47	-58.7	-26.7	-8.1	-314.6	-231.4	sI	
3	Kukuy K-2	VER14-03	2014St1GC5	240	97.5859	2.4025	0.0074	0.0031	0.0003	0.0008	n.d.	n.d.	40	-54.9	-25.8	-7.3	-304.6	-222.2	sI	
3	Kukuy K-2	VER14-03	2014St1GC6	241	86.0503	13.8920	0.0262	0.0133	0.0011	0.0171	n.d.	n.d.	6	-57.3	-26.2	-12.9	-310.4	-226.8	sII	
3	Kukuy K-2	VER14-03	2014St1GC6	246	96.8844	3.1142	0.0011	0.0001	0.0001	0.0001	n.d.	n.d.	31	-55.2	-26.6	n.d.	-305.7	-222.3	sI	
3	Kukuy K-2	VER14-03	2014St1GC6	252	96.3498	3.6485	0.0012	0.0003	0.0002	0.0001	n.d.	n.d.	26	-56.0	-26.4	n.d.	-307.8	-220.7	sI	
4	Kukuy K-0	VER06-02	2006St6GC1	195	99.9038	0.0962	n.d.	n.d.	n.d.	n.d.	n.d.	n.d.	1039	-65.9	-26.8	n.d.	n.d.	n.d.	sI	a)
4	Kukuy K-0	VER06-02	2006St6GC1	230	99.9079	0.0921	n.d.	n.d.	n.d.	n.d.	n.d.	n.d.	1084	-64.8	-27.1	n.d.	n.d.	n.d.	sI	a)
4	Kukuy K-0	VER06-02	2006St6GC3	85	99.9130	0.0870	n.d.	n.d.	n.d.	n.d.	n.d.	n.d.	1149	-66.4	-27.5	n.d.	n.d.	n.d.	sI	a)
4	Kukuy K-0	VER06-02	2006St6GC3	114	99.9241	0.0757	0.0003	n.d.	n.d.	n.d.	n.d.	n.d.	1316	-64.1	-27.6	n.d.	-326.3	-208.6	sI	a)
4	Kukuy K-0	VER06-02	2006St6GC3	150	99.9217	0.0781	0.0002	n.d.	n.d.	n.d.	n.d.	n.d.	1275	-64.3	-28.9	n.d.	-325.3	-211.9	sI	a)
4	Kukuy K-0	VER06-02	2006St6GC3	197	99.9278	0.0720	0.0003	n.d.	n.d.	n.d.	n.d.	n.d.	1384	-63.1	-28.6	n.d.	-324.5	-217.4	sI	a)
4	Kukuy K-0	VER06-02	2006St6GC3	261	99.9244	0.0756	n.d.	n.d.	n.d.	n.d.	n.d.	n.d.	1323	n.d.	-27.4	n.d.	n.d.	n.d.	sI	a)
4	Kukuy K-0	VER11-01	2011St4GC1	239	99.9029	0.0969	0.0002	n.d.	n.d.	n.d.	n.d.	n.d.	1029	-65.3	-28.7	n.d.	-327.5	-217.7	sI	
4	Kukuy K-0	VER11-01	2011St4GC4	160	99.8842	0.1158	n.d.	n.d.	n.d.	n.d.	n.d.	n.d.	863	-67.4	-29.6	n.d.	n.d.	n.d.	sI	
5	Gorevoy Utes	VER10-03	2010St1GC2	163	99.5119	0.4505	0.0238	0.0086	0.0030	n.d.	0.0021	n.d.	210	n.d.	-27.3	-21.2	n.d.	-193.9	sI	
5	Gorevoy Utes	VER10-03	2010St1GC2	201	99.5504	0.4243	0.0107	0.0014	0.0017	n.d.	0.0082	0.0033	229	-45.1	-27.0	-21.8	-306.2	-200.9	sI	
5	Gorevoy Utes	VER10-03	2010St1GC4	72	99.6017	0.3845	0.0081	0.0016	0.0005	n.d.	0.0035	0.0002	254	-44.6	-27.1	-22.0	-313.3	-200.9	sI	
5	Gorevoy Utes	VER10-03	2010St1GC4	74	99.6498	0.3346	0.0096	0.0012	0.0005	n.d.	0.0037	0.0006	289	-44.4	-27.2	-22.7	-311.4	-191.5	sI	
5	Gorevoy Utes	VER10-03	2010St1GC4	93	99.6005	0.3554	0.0079	0.0048	0.0040	n.d.	0.0229	0.0046	274	-45.0	-27.3	-22.3	-308.6	-194.2	sI	
5	Gorevoy Utes	VER10-03	2010St1GC4	114	99.5600	0.3884	0.0110	0.0066	0.0065	0.0002	0.0208	0.0065	249	-45.5	-27.5	-21.9	-307.2	-197.2	sI	
5	Gorevoy Utes	VER10-03	2010St1GC4	175	98.9509	0.3732	0.1025	0.1294	0.1126	0.0144	0.2398	0.0772	208	n.d.	-26.5	-22.6	-304.1	-195.5	sI	
5	Gorevoy Utes	VER10-03	2010St1GC4	unknown	99.5621	0.3990	0.0128	0.0049	0.0048	n.d.	0.0114	0.0050	242	-44.6	-27.3	-23.2	-307.9	-192.1	sI	
5	Gorevoy Utes	VER14-03	2014St14GC1	315	99.7971	0.1928	0.0059	0.0010	0.0009	0.0001	0.0019	0.0003	502	-44.9	-27.8	-19.2	-309.1	-221.7	sI	
5	Gorevoy Utes	VER14-03	2014St14GC1	330	99.6835	0.2760	0.0146	0.0090	0.0058	0.0003	0.0085	0.0023	343	-44.2	-27.6	-22.9	-300.6	-224.5	sI	

Table S2 (continue)

No.	Site	Cruise	Core	Sample Depth [cmblf]	Molecular Composition: CH_4 [%]	C_2H_6 [%]	C_3H_8 [%]	$i\text{-}C_4H_{10}$ [%]	$n\text{-}C_4H_{10}$ [%]	$neo\text{-}C_5H_{12}$ [%]	$i\text{-}C_5H_{12}$ [%]	$n\text{-}C_5H_{12}$ [%]	$C_1/(C_2+C_3)$	Isotopic Composition: CH_4 $\delta^{13}C$ [‰VPDB]	C_2H_6 $\delta^{13}C$ [‰VPDB]	C_3H_8 $\delta^{13}C$ [‰VPDB]	CH_4 δ^2H [‰VSMOW]	C_2H_6 δ^2H [‰VSMOW]	Crystallographic Structure	note
5	Gorevoy Utes	VER14-03	2014St14GC1	350	99.7447	0.2247	0.0125	0.0048	0.0047	0.0001	0.0065	0.0020	421	-44.3	-28.0	-22.5	-300.0	-222.7	sI	
5	Gorevoy Utes	VER14-03	2014St14GC1	370	99.8051	0.1790	0.0095	0.0024	0.0012	n.d.	0.0018	0.0010	529	-45.0	-28.3	-21.0	-307.5	-221.3	sI	
5	Gorevoy Utes	VER14-03	2014St14GC1	370	99.7929	0.1935	0.0097	0.0005	0.0009	0.0000	0.0019	0.0006	491	-44.5	-29.0	-21.9	-311.2	-225.6	sI	
6	Malyutka	VER06-02	2006St7GC1	125	99.9778	0.0222	n.d.	n.d.	n.d.	n.d.	n.d.	n.d.	4496	-67.1	-36.6	n.d.	n.d.	n.d.	sI	a)
6	Malyutka	VER06-02	2006St7GC1	144	99.9775	0.0209	0.0016	n.d.	n.d.	n.d.	n.d.	n.d.	4434	-65.4	-34.5	n.d.	-304.0	-239.2	sI	a)
6	Malyutka	VER06-02	2006St7GC3	170	99.9819	0.0174	0.0007	n.d.	n.d.	n.d.	n.d.	n.d.	5520	n.d.	-34.7	n.d.	-305.0	-246.0	sI	a)
7	Kukuy K-6	VER11-01	2011St19GC1	90	n.d.	n.d.	n.d.	n.d.	n.d.	n.d.	n.d.	n.d.	n.d.	-63.9	-31.3	n.d.	-320.4	-216.0	sI	
7	Kukuy K-6	VER11-01	2011St19GC1	100	99.9360	0.0638	0.0002	n.d.	n.d.	n.d.	n.d.	n.d.	1561	-70.3	-31.2	-13.8	-316.6	-217.2	sI	
7	Kukuy K-6	VER11-01	2011St19GC1	107	99.9436	0.0561	0.0003	n.d.	n.d.	n.d.	n.d.	n.d.	1773	-70.1	-31.3	-13.9	-316.8	-217.6	sI	
7	Kukuy K-6	VER11-01	2011St19GC1	114	99.9494	0.0503	0.0003	n.d.	n.d.	n.d.	n.d.	n.d.	1977	-69.6	-31.5	-19.6	-315.3	-219.4	sI	
8	Peschanka P-2	VER07-04	2007St2GC2	230	99.9279	0.0721	0.0000	n.d.	n.d.	n.d.	n.d.	n.d.	1386	-66.1	-62.6	n.d.	-310.2	n.d.	sI	a)
8	Peschanka P-2	VER07-04	2007St2GC2	243	99.9182	0.0818	0.0000	n.d.	n.d.	n.d.	n.d.	n.d.	1221	-67.8	-62.5	n.d.	n.d.	n.d.	sI	a)
8	Peschanka P-2	VER07-04	2007St2GC2	254	99.9338	0.0662	0.0000	n.d.	n.d.	n.d.	n.d.	n.d.	1510	-65.2	-62.7	n.d.	-311.8	n.d.	sI	a)
8	Peschanka P-2	VER07-04	2007St2GC5	297	99.9471	0.0529	0.0000	n.d.	n.d.	n.d.	n.d.	n.d.	1890	-66.8	-60.4	n.d.	-305.5	-289.2	sI	a)
8	Peschanka P-2	VER07-04	2007St2GC5	418	99.9556	0.0444	0.0000	n.d.	n.d.	n.d.	n.d.	n.d.	2250	-67.9	-62.7	n.d.	n.d.	n.d.	sI	a)
8	Peschanka P-2	VER07-04	2007St2GC5	423	99.9448	0.0552	0.0000	n.d.	n.d.	n.d.	n.d.	n.d.	1811	-67.7	-62.0	n.d.	-306.1	-294.1	sI	a)
8	Peschanka P-2	VER07-04	2007St2GC6	unknown	99.9627	0.0371	0.0001	n.d.	n.d.	n.d.	n.d.	n.d.	2682	-67.0	-62.6	n.d.	-304.8	-274.3	sI	a)
8	Peschanka P-2	VER07-04	2007St2GC11	412	99.9410	0.0590	0.0000	n.d.	n.d.	n.d.	n.d.	n.d.	1695	-68.1	-61.5	n.d.	n.d.	n.d.	sI	a)
8	Peschanka P-2	VER07-04	2007St2GC14	130	99.9625	0.0375	0.0000	n.d.	n.d.	n.d.	n.d.	n.d.	2667	n.d.	-62.2	n.d.	-308.6	n.d.	sI	a)
8	Peschanka P-2	VER07-04	2007St2GC14	150	99.9631	0.0366	0.0004	n.d.	n.d.	n.d.	n.d.	n.d.	2706	-67.2	-61.5	n.d.	-306.3	-281.0	sI	a)
8	Peschanka P-2	VER07-04	2007St2GC14	165	99.9632	0.0365	0.0003	n.d.	n.d.	n.d.	n.d.	n.d.	2717	-67.3	-60.2	n.d.	-304.9	-285.0	sI	a)
8	Peschanka P-2	VER07-04	2007St2GC14	252	99.9328	0.0671	0.0001	n.d.	n.d.	n.d.	n.d.	n.d.	1487	n.d.	-61.4	n.d.	-302.7	n.d.	sI	a)
8	Peschanka P-2	VER07-04	2007St2GC14	265	99.9419	0.0581	0.0000	n.d.	n.d.	n.d.	n.d.	n.d.	1719	-67.6	-62.3	n.d.	-304.3	-291.6	sI	a)
8	Peschanka P-2	VER07-04	2007St2GC14	287	99.9520	0.0480	0.0000	n.d.	n.d.	n.d.	n.d.	n.d.	2081	n.d.	-60.1	n.d.	-301.2	n.d.	sI	a)
8	Peschanka P-2	VER07-04	2007St2GC14	290	99.9518	0.0482	0.0000	n.d.	n.d.	n.d.	n.d.	n.d.	2074	-67.4	-60.0	n.d.	-303.1	-282.9	sI	a)
8	Peschanka P-2	VER07-04	2007St2GC14	290	99.9530	0.0468	0.0001	n.d.	n.d.	n.d.	n.d.	n.d.	2128	-66.9	-61.0	n.d.	-303.9	n.d.	sI	a)
9	Goloustnoe	VER07-04	2007St8GC1	289	99.0467	0.9503	0.0030	n.d.	n.d.	n.d.	n.d.	n.d.	104	-63.9	-28.6	n.d.	-306.0	-202.3	sI	a)
9	Goloustnoe	VER07-04	2007St8GC1	289	99.0243	0.9703	0.0054	n.d.	n.d.	n.d.	n.d.	n.d.	101	-64.3	-28.7	n.d.	-308.5	-201.6	sI	a)
9	Goloustnoe	winter expedition	2008St8wGC3	174	99.3384	0.6581	0.0035	n.d.	n.d.	n.d.	n.d.	n.d.	150	-64.1	-27.8	n.d.	-306.9	-210.5	sI	a)
9	Goloustnoe	winter expedition	2008St8wGC3	174	99.3322	0.6642	0.0035	n.d.	n.d.	n.d.	n.d.	n.d.	149	-64.4	-27.8	n.d.	-308.0	-212.2	sI	a)
9	Goloustnoe	winter expedition	2008St8wGC3	174	99.3295	0.6670	0.0035	n.d.	n.d.	n.d.	n.d.	n.d.	148	-64.4	-27.8	n.d.	-308.7	-212.9	sI	a)
9	Goloustnoe	winter expedition	2008St8wGC3	174	99.3238	0.6728	0.0035	n.d.	n.d.	n.d.	n.d.	n.d.	147	-64.3	-28.2	n.d.	-306.9	-226.0	sI	a)
9	Goloustnoe	winter expedition	2008St8wGC6	190	99.1541	0.8398	0.0061	n.d.	n.d.	n.d.	n.d.	n.d.	117	-64.3	-28.4	n.d.	-307.3	-213.9	sI	a)
9	Goloustnoe	winter expedition	2008St8wRGC2	130	98.8179	1.1806	0.0015	n.d.	n.d.	n.d.	n.d.	n.d.	84	-63.4	-28.0	n.d.	-306.0	-209.3	sI	a)
9	Goloustnoe	winter expedition	2008St8wRGC2	130	98.8329	1.1654	0.0017	n.d.	n.d.	n.d.	n.d.	n.d.	85	-63.3	-27.9	n.d.	-306.4	-210.6	sI	a)
9	Goloustnoe	winter expedition	2008St8wRGC2	130	98.8509	1.1474	0.0017	n.d.	n.d.	n.d.	n.d.	n.d.	86	-63.2	-28.0	n.d.	-306.9	-208.2	sI	a)
9	Goloustnoe	winter expedition	2008St8wHGC3	86	98.2410	1.7572	0.0018	n.d.	n.d.	n.d.	n.d.	n.d.	56	-63.8	-27.8	n.d.	-309.2	-216.5	sI	a)
9	Goloustnoe	winter expedition	2008St8wHGC3	86	98.2778	1.7205	0.0017	n.d.	n.d.	n.d.	n.d.	n.d.	57	-63.8	-27.8	n.d.	-309.7	-214.7	sI	a)
9	Goloustnoe	winter expedition	2008St8wHGC3	86	98.3123	1.6860	0.0017	n.d.	n.d.	n.d.	n.d.	n.d.	58	-63.9	-27.7	n.d.	-309.8	-213.5	sI	a)
9	Goloustnoe	winter expedition	2008St8wHGC3	86	98.6640	1.3334	0.0025	n.d.	n.d.	n.d.	n.d.	n.d.	74	-64.0	-28.2	n.d.	-308.8	-206.1	sI	a)

Table S2 (continue)

No.	Site	Cruise	Core	Sample Depth [cmblf]	Molecular Composition CH_4 [%]	C_2H_6 [%]	C_3H_8 [%]	$i\text{-}C_4H_{10}$ [%]	$n\text{-}C_4H_{10}$ [%]	$neo\text{-}C_5H_{12}$ [%]	$i\text{-}C_5H_{12}$ [%]	$n\text{-}C_5H_{12}$ [%]	$C_1/(C_2+C_3)$	Isotopic Composition CH_4 $\delta^{13}C$ [‰VPDB]	C_2H_6 $\delta^{13}C$ [‰VPDB]	C_3H_8 $\delta^{13}C$ [‰VPDB]	CH_4 δ^2H [‰VSMOW]	C_2H_6 δ^2H [‰VSMOW]	Crystallographic Structure	note
9	Goloustnoe	VER08-05	2008St2GC1	125	98.7880	1.2073	0.0046	n.d.	n.d.	n.d.	n.d.	n.d.	82	-64.2	-27.8	n.d.	-307.1	-211.2	sI	a)
9	Goloustnoe	VER08-05	2008St2GC1	125	98.5424	1.4532	0.0044	n.d.	n.d.	n.d.	n.d.	n.d.	68	-64.2	-27.8	n.d.	-307.7	-209.4	sI	a)
9	Goloustnoe	VER08-05	2008St2GC1	131	98.7656	1.2311	0.0033	n.d.	n.d.	n.d.	n.d.	n.d.	80	-64.4	-27.4	n.d.	-308.8	-209.1	sI	a)
9	Goloustnoe	VER09-03	2009St3GC1	107	n.d.	n.d.	n.d.	n.d.	n.d.	n.d.	n.d.	n.d.	n.d.	-64.2	-26.7	n.d.	-304.2	-221.4	sI	
9	Goloustnoe	VER09-03	2009St3GC1	113	n.d.	n.d.	n.d.	n.d.	n.d.	n.d.	n.d.	n.d.	n.d.	-64.9	-26.8	n.d.	-307.3	-217.8	sI	
10	St. Petersburg	VER13-03	2013St41GC4	40	99.5480	0.4493	0.0023	0.0003	0.0001	0.0001	0.0000	n.d.	220	-67.1	-30.1	3.5	-287.0	-199.4	sI	
10	St. Petersburg	VER13-03	2013St41GC4	48	99.3822	0.6169	0.0009	0.0000	0.0000	n.d.	n.d.	n.d.	161	-66.4	-30.5	-3.9	-283.8	-212.6	sI	
10	St. Petersburg	VER13-03	2013St41GC4	48	99.5993	0.4000	0.0004	n.d.	0.0003	0.0000	0.0000	n.d.	249	-66.8	n.d.	n.d.	-289.8	-191.9	sI	
10	St. Petersburg	VER13-03	2013St41GC4	55	99.6980	0.2993	0.0025	0.0001	n.d.	0.0001	0.0000	n.d.	330	-66.9	-30.2	4.8	-285.7	-199.5	sI	
10	St. Petersburg	VER13-03	2013St41GC4	60	99.2733	0.7266	0.0001	n.d.	n.d.	n.d.	n.d.	n.d.	137	-66.5	-27.6	n.d.	-287.3	-182.5	sI	
11	Kukuy K-10	VER10-03	2010St11GC1	224	86.2232	13.7636	0.0038	n.d.	n.d.	0.0093	n.d.	n.d.	6	-53.5	-26.0	3.1	-296.9	-207.3	sII	
11	Kukuy K-10	VER10-03	2010St11GC1	235	95.5804	4.4192	0.0004	n.d.	n.d.	n.d.	n.d.	n.d.	22	n.d.	-26.0	7.6	-296.9	-201.5	sI	
11	Kukuy K-10	VER10-03	2010St11GC1	250	96.8615	3.1383	0.0002	n.d.	n.d.	n.d.	n.d.	n.d.	31	-53.4	-27.2	11.0	-296.8	-201.7	sI	
11	Kukuy K-10	VER10-03	2010St11GC1	262	95.3115	4.6882	0.0003	n.d.	n.d.	n.d.	n.d.	n.d.	20	0.0	-27.7	7.8	-300.7	-203.4	sI	
11	Kukuy K-10	VER12-03	2012St1GC1	153	98.4155	1.5836	0.0005	n.d.	0.0004	n.d.	n.d.	n.d.	62	-54.6	-24.0	n.d.	-303.5	-206.8	sI	
11	Kukuy K-10	VER12-03	2012St1GC1	155	98.3629	1.6345	0.0007	n.d.	0.0020	n.d.	n.d.	n.d.	60	-57.2	-24.9	n.d.	n.d.	n.d.	sI	
11	Kukuy K-10	VER12-03	2012St1GC1	177	99.0588	0.9403	0.0003	0.0002	0.0001	0.0003	n.d.	n.d.	105	-58.2	-24.7	-4.1	-307.2	-206.1	sI	
12	Kukuy K-1	VER10-03	2010St9GC1	156	99.5279	0.4716	0.0005	n.d.	n.d.	n.d.	n.d.	n.d.	211	-64.4	-27.6	n.d.	-314.1	-209.9	sI	
12	Kukuy K-1	VER10-03	2010St9GC1	168	99.5106	0.4889	0.0005	n.d.	n.d.	n.d.	n.d.	n.d.	203	-60.7	-24.6	n.d.	-316.5	-208.8	sI	
12	Kukuy K-1	VER10-03	2010St9GC1	168	99.5388	0.4607	0.0005	n.d.	n.d.	n.d.	n.d.	n.d.	216	-59.5	-25.4	n.d.	-316.7	-212.1	sI	
12	Kukuy K-1	VER10-03	2010St9GC1	175	99.5408	0.4587	0.0005	n.d.	n.d.	n.d.	n.d.	n.d.	217	-62.2	-23.5	-4.9	-310.8	-211.3	sI	
13	Novosibirsk	VER10-03	2010St2GC8	224	99.4595	0.5401	0.0004	n.d.	n.d.	n.d.	n.d.	n.d.	184	-69.0	-33.8	n.d.	-296.6	-223.7	sI	
13	Novosibirsk	VER10-03	2010St2GC8	227	99.7828	0.2159	0.0013	n.d.	n.d.	n.d.	n.d.	n.d.	459	-68.1	-33.8	n.d.	-290.0	-216.1	sI	
13	Novosibirsk	VER10-03	2010St2GC8	244	99.7553	0.2442	0.0005	n.d.	n.d.	n.d.	n.d.	n.d.	408	-67.6	-33.6	n.d.	-292.1	-224.9	sI	
13	Novosibirsk	VER10-03	2010St2GC8	244	99.7185	0.2812	0.0004	n.d.	n.d.	n.d.	n.d.	n.d.	354	-67.4	-33.4	n.d.	-293.4	-222.6	sI	
13	Novosibirsk	VER10-03	2010St2GC8	244	99.6763	0.3228	0.0009	n.d.	n.d.	n.d.	n.d.	n.d.	308	-68.6	-33.4	n.d.	-294.6	-225.4	sI	
13	Novosibirsk	VER10-03	2010St2GC8	244	99.5623	0.4348	0.0023	0.0006	n.d.	n.d.	n.d.	n.d.	228	-67.3	-33.9	-8.5	-294.7	-225.2	sI	
13	Novosibirsk	VER10-03	2010St2GC11	178	99.6578	0.3410	0.0011	n.d.	n.d.	n.d.	n.d.	n.d.	291	-67.2	-33.8	-6.7	-290.7	-228.7	sI	
13	Novosibirsk	VER10-03	2010St2GC11	178	n.d.	n.d.	n.d.	n.d.	n.d.	n.d.	n.d.	n.d.	n.d.	n.d.	n.d.	n.d.	n.d.	-224.7	sI	
13	Novosibirsk	VER10-03	2010St2GC11	178	n.d.	n.d.	n.d.	n.d.	n.d.	n.d.	n.d.	n.d.	n.d.	n.d.	n.d.	n.d.	-292.8	-221.0	sI	
13	Novosibirsk	VER10-03	2010St2GC12	149	99.7825	0.2166	0.0009	0.0000	n.d.	n.d.	n.d.	n.d.	459	-67.0	-33.3	-5.3	-294.9	-223.0	sI	
13	Novosibirsk	VER10-03	2010St2GC12	149	99.7452	0.2538	0.0010	n.d.	n.d.	n.d.	n.d.	n.d.	392	-68.1	-32.5	n.d.	-295.3	-225.3	sI	
13	Novosibirsk	VER10-03	2010St2GC12	149	n.d.	n.d.	n.d.	n.d.	n.d.	n.d.	n.d.	n.d.	n.d.	n.d.	n.d.	n.d.	n.d.	-222.6	sI	
13	Novosibirsk	VER10-03	2010St2GC12	149	n.d.	n.d.	n.d.	n.d.	n.d.	n.d.	n.d.	n.d.	n.d.	n.d.	n.d.	n.d.	n.d.	-230.5	sI	
13	Novosibirsk	VER10-03	2010St2GC18	83	99.6606	0.3382	0.0012	n.d.	n.d.	n.d.	n.d.	n.d.	294	-68.6	-34.2	n.d.	-292.6	-224.7	sI	
13	Novosibirsk	VER10-03	2010St2GC18	90	99.7560	0.2433	0.0006	n.d.	n.d.	n.d.	n.d.	n.d.	409	-67.6	-34.7	n.d.	-291.2	-227.8	sI	
13	Novosibirsk	VER10-03	2010St2GC18	110	99.7494	0.2501	0.0005	n.d.	n.d.	n.d.	n.d.	n.d.	398	-68.5	-35.3	n.d.	-292.6	-230.9	sI	
13	Novosibirsk	VER10-03	2010St2GC18	110	99.6954	0.3040	0.0006	n.d.	n.d.	n.d.	n.d.	n.d.	327	-67.2	-32.0	-12.0	-291.6	-232.5	sI	
13	Novosibirsk	VER10-03	2010St2GC22	245	99.6475	0.3521	0.0004	n.d.	n.d.	n.d.	n.d.	n.d.	283	-67.6	-32.4	-11.3	-293.5	-221.1	sI	
13	Novosibirsk	VER11-01	2011St15GC1	135	99.7367	0.2614	0.0019	n.d.	n.d.	n.d.	n.d.	n.d.	379	-69.3	-32.1	-4.9	-290.9	-229.5	sI	

Table S2 (continue)

No.	Site	Cruise	Core	Sample Depth [cmblf]	Molecular Composition CH$_4$ [%]	C$_2$H$_6$ [%]	C$_3$H$_8$ [%]	i-C$_4$H$_{10}$ [%]	n-C$_4$H$_{10}$ [%]	neo-C$_5$H$_{12}$ [%]	i-C$_5$H$_{12}$ [%]	n-C$_5$H$_{12}$ [%]	C$_1$/(C$_2$+C$_3$)	Isotopic Composition CH$_4$ δ^{13}C [‰VPDB]	C$_2$H$_6$ δ^{13}C [‰VPDB]	C$_3$H$_8$ δ^{13}C [‰VPDB]	CH$_4$ δ^{2}H [‰VSMOW]	C$_2$H$_6$ δ^{2}H [‰VSMOW]	Crystallographic Structure	note
13	Novosibirsk	VER11-01	2011St15GC1	138	99.6234	0.3761	0.0005	n.d.	n.d.	n.d.	n.d.	n.d.	265	-70.4	-31.5	-8.2	-293.7	-219.1	sI	
13	Novosibirsk	VER11-01	2011St15GC1	148	n.d.	n.d.	n.d.	n.d.	n.d.	n.d.	n.d.	n.d.	n.d.	-67.6	-31.6	n.d.	-292.8	-230.0	sI	
14	Kukuy K-8	VER10-03	2010St10GC1	262	98.7286	1.2712	0.0003	n.d.	n.d.	n.d.	n.d.	n.d.	78	-63.1	-26.9	-14.1	-314.6	-212.0	sI	
14	Kukuy K-8	VER10-03	2010St10GC1	262	98.7542	1.2454	0.0003	n.d.	n.d.	n.d.	n.d.	n.d.	79	-62.9	-26.6	n.d.	-317.2	-211.2	sI	
14	Kukuy K-8	VER15-03	2015St17GC2	101	99.1428	0.8570	0.0001	n.d.	n.d.	n.d.	n.d.	n.d.	116	-63.5	-25.1	n.d.	-317.1	-224.2	sI	
14	Kukuy K-8	VER15-03	2015St17GC2	113	99.3739	0.6259	0.0002	0.0000	n.d.	n.d.	n.d.	n.d.	159	-63.5	-25.1	n.d.	-315.6	-228.8	sI	
14	Kukuy K-8	VER15-03	2015St17GC2	113	99.3732	0.6267	0.0001	0.0000	n.d.	n.d.	n.d.	n.d.	159	-63.3	-25.0	n.d.	-315.9	-230.4	sI	
15	Kukuy K-9	VER10-03	2010St6GC2	170	99.9747	0.0250	0.0003	n.d.	n.d.	n.d.	n.d.	n.d.	3957	n.d.	-32.7	n.d.	n.d.	-214.8	sI	
15	Kukuy K-9	VER10-03	2010St6GC2	179	99.9781	0.0213	0.0006	n.d.	n.d.	n.d.	n.d.	n.d.	4565	n.d.	-35.3	n.d.	n.d.	-208.7	sI	
15	Kukuy K-9	VER10-03	2010St6GC2	193	99.9813	0.0185	0.0002	n.d.	n.d.	n.d.	n.d.	n.d.	5354	-68.0	-33.1	n.d.	-314.5	n.d.	sI	
15	Kukuy K-9	VER10-03	2010St6GC2	193	99.9785	0.0214	0.0001	n.d.	n.d.	n.d.	n.d.	n.d.	4645	-67.1	-36.6	n.d.	-314.2	-206.3	sI	
15	Kukuy K-9	VER10-03	2010St6GC3	100	99.9796	0.0202	0.0002	n.d.	n.d.	n.d.	n.d.	n.d.	4910	-66.6	-34.6	n.d.	-314.7	-221.8	sI	
15	Kukuy K-9	VER10-03	2010St6GC3	110	99.9846	0.0153	0.0002	n.d.	n.d.	n.d.	n.d.	n.d.	6475	-66.6	-34.7	n.d.	-312.8	-217.0	sI	
15	Kukuy K-9	VER10-03	2010St6GC3	170	99.9850	0.0147	0.0003	n.d.	n.d.	n.d.	n.d.	n.d.	6662	-65.7	-34.2	n.d.	-312.3	-218.1	sI	
15	Kukuy K-9	VER10-03	2010St6GC3	180	99.9770	0.0226	0.0004	n.d.	n.d.	n.d.	n.d.	n.d.	4342	n.d.	n.d.	n.d.	-310.3	-213.9	sI	
15	Kukuy K-9	VER10-03	2010St6GC3	180	99.9825	0.0173	0.0002	n.d.	n.d.	n.d.	n.d.	n.d.	5714	-67.4	-34.7	-30.9	-312.6	-207.1	sI	
15	Kukuy K-9	VER10-03	2010St6GC4	129	99.9884	0.0112	0.0004	n.d.	n.d.	n.d.	n.d.	n.d.	8626	-67.6	-34.7	n.d.	-310.4	-227.4	sI	
15	Kukuy K-9	VER10-03	2010St6GC4	146	99.9840	0.0158	0.0002	n.d.	n.d.	n.d.	n.d.	n.d.	6243	-67.8	-36.8	n.d.	-314.9	-221.7	sI	
15	Kukuy K-9	VER10-03	2010St6GC4	163	99.9855	0.0143	0.0002	n.d.	n.d.	n.d.	n.d.	n.d.	6872	-67.2	-37.2	n.d.	-315.1	-217.0	sI	
15	Kukuy K-9	VER10-03	2010St6GC4	165	99.9842	0.0157	0.0002	n.d.	n.d.	n.d.	n.d.	n.d.	6311	-66.5	-36.3	-31.6	-315.6	-208.1	sI	
15	Kukuy K-9	VER10-03	2010St6GC5	40	99.9761	0.0235	0.0003	n.d.	n.d.	n.d.	n.d.	n.d.	4192	-67.6	n.d.	n.d.	-316.3	-224.7	sI	
15	Kukuy K-9	VER10-03	2010St6GC5	90	99.9497	0.0487	0.0016	n.d.	n.d.	n.d.	n.d.	n.d.	1989	n.d.	n.d.	n.d.	n.d.	n.d.	sI	
15	Kukuy K-9	VER10-03	2010St6GC5	120	99.9810	0.0186	0.0003	n.d.	n.d.	n.d.	n.d.	n.d.	5274	n.d.	n.d.	n.d.	-312.7	n.d.	sI	
15	Kukuy K-9	VER10-03	2010St6GC5	151	99.9866	0.0132	0.0003	n.d.	n.d.	n.d.	n.d.	n.d.	7436	-67.8	-35.7	n.d.	-313.4	-212.4	sI	
15	Kukuy K-9	VER10-03	2010St6GC5	167	99.9856	0.0141	0.0003	n.d.	n.d.	n.d.	n.d.	n.d.	6932	-66.9	-35.2	-26.1	-313.1	-209.4	sI	
15	Kukuy K-9	VER10-03	2010St6GC6	80	99.9869	0.0128	0.0003	n.d.	n.d.	n.d.	n.d.	n.d.	7618	-65.9	-35.7	n.d.	-309.3	n.d.	sI	
15	Kukuy K-9	VER10-03	2010St6GC6	110	99.9836	0.0159	0.0005	n.d.	n.d.	n.d.	n.d.	n.d.	6100	-65.4	-32.9	n.d.	-308.0	n.d.	sI	
15	Kukuy K-9	VER10-03	2010St6GC6	150	99.9835	0.0162	0.0003	n.d.	n.d.	n.d.	n.d.	n.d.	6052	-66.5	-33.2	n.d.	-312.2	n.d.	sI	
15	Kukuy K-9	VER10-03	2010St6GC6	176	99.9850	0.0147	0.0003	n.d.	n.d.	n.d.	n.d.	n.d.	6650	-67.5	-35.4	n.d.	-313.5	-216.0	sI	
15	Kukuy K-9	VER10-03	2010St6GC6	200	99.9877	0.0119	0.0004	n.d.	n.d.	n.d.	n.d.	n.d.	8119	-66.7	-35.1	-25.5	-311.0	-211.4	sI	
15	Kukuy K-9	VER10-03	2010St6GC7	130	99.9694	0.0300	0.0006	n.d.	n.d.	n.d.	n.d.	n.d.	3264	n.d.	n.d.	n.d.	n.d.	n.d.	sI	
15	Kukuy K-9	VER10-03	2010St6GC7	150	99.9736	0.0262	0.0002	n.d.	n.d.	n.d.	n.d.	n.d.	3781	-66.0	n.d.	n.d.	-305.4	n.d.	sI	
15	Kukuy K-9	VER10-03	2010St6GC7	175	99.9745	0.0252	0.0003	n.d.	n.d.	n.d.	n.d.	n.d.	3917	n.d.	n.d.	n.d.	-307.3	-212.0	sI	
15	Kukuy K-9	VER10-03	2010St6GC7	176	99.9814	0.0184	0.0002	n.d.	n.d.	n.d.	n.d.	n.d.	5384	-67.1	-31.8	n.d.	-313.9	-206.5	sI	
15	Kukuy K-9	VER10-03	2010St6GC7	197	99.9692	0.0302	0.0006	n.d.	n.d.	n.d.	n.d.	n.d.	3248	n.d.	n.d.	n.d.	n.d.	-204.1	sI	
15	Kukuy K-9	VER10-03	2010St6GC7	226	99.9858	0.0140	0.0003	n.d.	n.d.	n.d.	n.d.	n.d.	7025	-66.9	-33.9	n.d.	-310.5	-211.1	sI	
15	Kukuy K-9	VER10-03	2010St6GC7	230	99.9843	0.0155	0.0002	n.d.	n.d.	n.d.	n.d.	n.d.	6372	-67.0	-34.2	-22.1	-311.9	-209.6	sI	
15	Kukuy K-9	VER10-03	2010St6GC8	109	99.9686	0.0309	0.0006	n.d.	n.d.	n.d.	n.d.	n.d.	3181	n.d.	n.d.	n.d.	-307.9	-217.9	sI	
15	Kukuy K-9	VER10-03	2010St6GC8	153	99.9830	0.0167	0.0003	n.d.	n.d.	n.d.	n.d.	n.d.	5882	-67.5	-36.4	n.d.	-313.1	-213.0	sI	
15	Kukuy K-9	VER10-03	2010St6GC8	190	99.9837	0.0159	0.0004	n.d.	n.d.	n.d.	n.d.	n.d.	6151	-67.1	-35.8	-24.4	-310.9	-206.6	sI	
15	Kukuy K-9	VER10-03	2010St6GC10	54	99.9803	0.0194	0.0003	n.d.	n.d.	n.d.	n.d.	n.d.	5080	n.d.	n.d.	n.d.	-315.2	-227.4	sI	
15	Kukuy K-9	VER10-03	2010St6GC10	75	99.9822	0.0177	0.0001	n.d.	n.d.	n.d.	n.d.	n.d.	5612	-67.5	-36.4	n.d.	-317.0	-211.7	sI	

Table S2 (continue)

No.	Site	Cruise	Core	Sample Depth [cmblf]	Molecular Composition CH$_4$ [%]	C$_2$H$_6$ [%]	C$_3$H$_8$ [%]	i-C$_4$H$_{10}$ [%]	n-C$_4$H$_{10}$ [%]	neo-C$_5$H$_{12}$ [%]	i-C$_5$H$_{12}$ [%]	n-C$_5$H$_{12}$ [%]	C$_1$/(C$_2$+C$_3$)	Isotopic Composition CH$_4$ δ^{13}C [‰VPDB]	C$_2$H$_6$ δ^{13}C [‰VPDB]	C$_3$H$_8$ δ^{13}C [‰VPDB]	CH$_4$ δ^{2}H [‰VSMOW]	C$_2$H$_6$ δ^{2}H [‰VSMOW]	Crystallographic Structure	note
15	Kukuy K-9	VER10-03	2010St6GC10	98	99.9848	0.0150	0.0001	n.d.	n.d.	n.d.	n.d.	n.d.	6597	-67.0	-38.4	n.d.	-314.5	-206.1	sI	
15	Kukuy K-9	VER10-03	2010St6GC10	100	99.9889	0.0108	0.0004	n.d.	n.d.	n.d.	n.d.	n.d.	8994	n.d.	-33.8	-27.5	-308.4	n.d.	sI	
15	Kukuy K-9	VER10-03	2010St6GC11	89	99.9777	0.0223	0.0001	n.d.	n.d.	n.d.	n.d.	n.d.	4478	-67.5	-38.2	n.d.	-316.4	-206.9	sI	
15	Kukuy K-9	VER10-03	2010St6GC11	121	99.9817	0.0180	0.0003	n.d.	n.d.	n.d.	n.d.	n.d.	5472	n.d.	-35.1	n.d.	-314.2	-206.1	sI	
15	Kukuy K-9	VER10-03	2010St6GC12	125	99.9844	0.0152	0.0003	n.d.	n.d.	n.d.	n.d.	n.d.	6416	n.d.	n.d.	n.d.	n.d.	n.d.	sI	
15	Kukuy K-9	VER10-03	2010St6GC12	179	99.9786	0.0213	0.0001	n.d.	n.d.	n.d.	n.d.	n.d.	4662	-67.7	-36.1	n.d.	-314.8	-222.1	sI	
15	Kukuy K-9	VER10-03	2010St6GC12	185	99.9778	0.0220	0.0001	n.d.	n.d.	n.d.	n.d.	n.d.	4513	n.d.	n.d.	n.d.	-315.4	-203.4	sI	
15	Kukuy K-9	VER10-03	2010St6GC12	186	99.9785	0.0214	0.0001	n.d.	n.d.	n.d.	n.d.	n.d.	4647	n.d.	-35.4	n.d.	-312.0	n.d.	sI	
15	Kukuy K-9	VER10-03	2010St6GC13	84	99.9804	0.0195	0.0001	n.d.	n.d.	n.d.	n.d.	n.d.	5098	-66.9	-36.4	n.d.	-315.4	-229.8	sI	
15	Kukuy K-9	VER10-03	2010St6GC13	177	99.9813	0.0185	0.0002	n.d.	n.d.	n.d.	n.d.	n.d.	5342	n.d.	n.d.	n.d.	n.d.	n.d.	sI	
15	Kukuy K-9	VER10-03	2010St6GC13	216	99.9848	0.0149	0.0002	n.d.	n.d.	n.d.	n.d.	n.d.	6581	-66.4	-36.0	n.d.	-313.8	-202.3	sI	
15	Kukuy K-9	VER10-03	2010St6GC14	182	99.9761	0.0238	0.0001	n.d.	n.d.	n.d.	n.d.	n.d.	4189	-67.1	-39.3	n.d.	-317.6	-213.8	sI	
15	Kukuy K-9	VER10-03	2010St6GC14	199	99.9604	0.0394	0.0002	n.d.	n.d.	n.d.	n.d.	n.d.	2521	n.d.	n.d.	n.d.	n.d.	-199.1	sI	
15	Kukuy K-9	VER10-03	2010St6GC15	110	99.9198	0.0800	0.0002	n.d.	n.d.	n.d.	n.d.	n.d.	1246	n.d.	n.d.	n.d.	-309.4	n.d.	sI	
15	Kukuy K-9	VER10-03	2010St6GC15	140	99.9622	0.0375	0.0003	n.d.	n.d.	n.d.	n.d.	n.d.	2641	n.d.	n.d.	n.d.	-309.4	n.d.	sI	
16	PosolBank	VER12-03	2012St20GC1	85	95.4573	4.5421	0.0001	0.0003	0.0001	0.0000	n.d.	n.d.	21	-46.8	-25.4	n.d.	-278.7	-205.2	sI	
16	PosolBank	VER12-03	2012St20GC1	100	95.6018	4.3980	0.0000	n.d.	0.0001	0.0001	n.d.	n.d.	22	-47.5	-26.0	n.d.	-280.6	-205.1	sI	
16	PosolBank	VER12-03	2012St20GC1	114	95.7069	4.2929	0.0000	n.d.	0.0001	0.0001	n.d.	n.d.	22	-46.5	-22.8	n.d.	-278.9	-201.7	sI	
16	PosolBank	VER12-03	2012St20GC1	165	96.6194	3.3801	0.0006	n.d.	n.d.	n.d.	n.d.	n.d.	29	-45.1	-25.3	-25.5	-271.7	-206.1	sI	
16	PosolBank	VER12-03	2012St20GC2	212	87.0450	12.9168	0.0169	0.0180	0.0028	0.0005	n.d.	n.d.	7	-45.7	-23.4	-15.3	-274.8	-214.8	sII	
16	PosolBank	VER12-03	2012St20GC2	220	86.1600	13.7739	0.0312	0.0279	0.0053	0.0013	0.0004	n.d.	6	-47.0	-24.0	-23.8	-274.8	-212.4	sII	
16	PosolBank	VER12-03	2012St20GC2	230	86.0030	13.9716	0.0122	0.0016	0.0004	0.0111	n.d.	n.d.	6	-43.5	-26.0	-14.1	-268.1	-208.5	sII	
16	PosolBank	VER12-03	2012St20GC2	252	87.2193	12.7438	0.0203	0.0113	0.0045	0.0008	0.0001	n.d.	7	-46.1	-23.9	-15.8	-273.0	-212.1	sII	
16	PosolBank	VER12-03	2012St20GC5	83	86.3942	13.5603	0.0246	0.0060	0.0013	0.0136	n.d.	n.d.	6	-44.7	-26.1	-15.0	-270.5	-208.9	sII	
16	PosolBank	VER12-03	2012St20GC5	93	96.3713	3.6150	0.0094	0.0020	0.0018	0.0003	0.0001	n.d.	27	-49.5	-24.1	-15.9	-276.7	-209.9	sI	
16	PosolBank	VER12-03	2012St20GC5	110	96.9147	3.0727	0.0068	0.0015	0.0004	0.0035	0.0004	n.d.	31	-45.1	-26.4	-14.1	-269.5	-209.9	sI	
17	Kukuy K-4	VER11-01	2011St8GC3	135	86.0164	13.9736	0.0100	n.d.	n.d.	n.d.	n.d.	n.d.	6	-48.2	-25.5	0.5	-284.6	-204.9	sII	
17	Kukuy K-4	VER11-01	2011St8GC3	175	96.7615	3.2374	0.0011	n.d.	n.d.	n.d.	n.d.	n.d.	30	-48.6	-27.4	-1.5	-285.5	-199.6	sI	
17	Kukuy K-4	VER11-01	2011St8GC3	205	96.7562	3.2421	0.0017	n.d.	n.d.	n.d.	n.d.	n.d.	30	-48.5	-27.4	-2.3	-283.3	-200.8	sI	
17	Kukuy K-4	VER11-01	2011St8GC3	214	97.0735	2.9252	0.0013	n.d.	n.d.	n.d.	n.d.	n.d.	33	-48.5	-27.4	-5.3	-285.2	-198.6	sI	
17	Kukuy K-4	VER11-01	2011St8GC5	25	85.8302	14.1605	0.0093	n.d.	n.d.	n.d.	n.d.	n.d.	6	-48.0	-25.5	n.d.	-284.4	-208.5	sII	
17	Kukuy K-4	VER11-01	2011St8GC5	25	88.0305	11.9639	0.0056	n.d.	n.d.	n.d.	n.d.	n.d.	7	-47.8	-25.5	1.8	-286.3	-210.2	sII	
17	Kukuy K-4	VER11-01	2011St8GC5	30	85.9747	14.0165	0.0088	n.d.	n.d.	n.d.	n.d.	n.d.	6	-48.0	-25.4	n.d.	-286.7	-209.6	sII	
17	Kukuy K-4	VER11-01	2011St8GC5	30	94.5285	5.4690	0.0025	n.d.	n.d.	n.d.	n.d.	n.d.	17	-48.2	-26.6	2.7	n.d.	n.d.	sI	
17	Kukuy K-4	VER11-01	2011St8GC5	70	94.8179	5.1773	0.0048	n.d.	n.d.	n.d.	n.d.	n.d.	18	-47.5	-26.7	0.5	-285.4	-208.1	sI	
17	Kukuy K-4	VER11-01	2011St8GC5	80	95.5847	4.4135	0.0018	n.d.	n.d.	n.d.	n.d.	n.d.	22	-48.2	-27.0	3.7	-284.3	-210.3	sI	
17	Kukuy K-4	VER11-01	2011St8GC5	90	94.7660	5.2279	0.0062	n.d.	n.d.	n.d.	n.d.	n.d.	18	-48.5	-27.0	1.1	-286.5	-208.9	sI	
17	Kukuy K-4	VER11-01	2011St8GC5	110	96.4414	3.5546	0.0040	n.d.	n.d.	n.d.	n.d.	n.d.	27	-48.1	-26.9	1.2	-286.9	-206.6	sI	
17	Kukuy K-4	VER11-01	2011St8GC5	120	96.6505	3.3473	0.0023	n.d.	n.d.	n.d.	n.d.	n.d.	29	-48.3	-27.3	1.8	-284.2	-206.1	sI	
18	Kukuy K-11	VER11-01	2011St2GC3	63	94.1362	5.8597	0.0041	n.d.	n.d.	n.d.	n.d.	n.d.	16	-59.9	-26.3	n.d.	-319.7	-218.8	sI	
18	Kukuy K-11	VER11-01	2011St2GC3	83	95.4785	4.5189	0.0025	n.d.	n.d.	n.d.	n.d.	n.d.	21	-60.6	-26.2	-8.7	-316.5	-212.3	sI	

Table S2 (continue)

No.	Site	Cruise	Core	Sample Depth [cmblf]	Molecular Composition CH$_4$ [%]	C$_2$H$_6$ [%]	C$_3$H$_8$ [%]	i-C$_4$H$_{10}$ [%]	n-C$_4$H$_{10}$ [%]	neo-C$_5$H$_{12}$ [%]	i-C$_5$H$_{12}$ [%]	n-C$_5$H$_{12}$ [%]	C$_1$/(C$_2$+C$_3$)	Isotopic Composition CH$_4$ δ^{13}C [‰VPDB]	C$_2$H$_6$ δ^{13}C [‰VPDB]	C$_3$H$_8$ δ^{13}C [‰VPDB]	CH$_4$ δ^2H [‰VSMOW]	C$_2$H$_6$ δ^2H [‰VSMOW]	Crystallographic Structure	note
18	Kukuy K-11	VER11-01	2011St2GC3	83	n.d.	n.d.	n.d.	n.d.	n.d.	n.d.	n.d.	n.d.	n.d.	n.d.	-22.2	n.d.	-316.9	-201.7	sI	
18	Kukuy K-11	VER11-01	2011St2GC3	110	98.3989	1.5988	0.0022	n.d.	n.d.	n.d.	n.d.	n.d.	61	-62.8	-25.9	-8.4	-317.5	-210.5	sI	
18	Kukuy K-11	VER11-01	2011St2GC3	110	n.d.	n.d.	n.d.	n.d.	n.d.	n.d.	n.d.	n.d.	n.d.	-63.1	-25.9	n.d.	-313.4	-201.2	sI	
18	Kukuy K-11	VER11-01	2011St2GC4	130	97.9690	2.0270	0.0040	n.d.	n.d.	n.d.	n.d.	n.d.	48	-61.6	-26.1	-8.6	-316.0	-210.3	sI	
18	Kukuy K-11	VER11-01	2011St2GC4	130	n.d.	n.d.	n.d.	n.d.	n.d.	n.d.	n.d.	n.d.	n.d.	-62.8	-26.1	n.d.	-313.0	-203.8	sI	
18	Kukuy K-11	VER11-01	2011St2GC4	160	98.6609	1.3378	0.0014	n.d.	n.d.	n.d.	n.d.	n.d.	74	-63.0	-25.7	-10.3	-316.0	-210.0	sI	
19	Kukuy K-12	VER11-01	2011St5GC3	80	n.d.	n.d.	n.d.	n.d.	n.d.	n.d.	n.d.	n.d.	n.d.	-63.3	-26.6	n.d.	-277.7	-172.7	sI	
19	Kukuy K-12	VER11-01	2011St5GC3	110	n.d.	n.d.	n.d.	n.d.	n.d.	n.d.	n.d.	n.d.	n.d.	-63.2	-26.8	n.d.	-279.0	-183.1	sI	
19	Kukuy K-12	VER11-01	2011St5GC3	110	99.6726	0.3270	0.0005	n.d.	n.d.	n.d.	n.d.	n.d.	304	-63.6	-26.6	-2.8	-281.5	-188.6	sI	
19	Kukuy K-12	VER11-01	2011St5GC3	130	n.d.	n.d.	n.d.	n.d.	n.d.	n.d.	n.d.	n.d.	n.d.	-63.5	-26.2	n.d.	-283.4	-186.7	sI	
19	Kukuy K-12	VER11-01	2011St5GC3	135	99.6491	0.3505	0.0004	n.d.	n.d.	n.d.	n.d.	n.d.	284	-63.0	-25.8	-3.1	-281.3	-184.9	sI	
20	Seep13	VER11-01	2011St13GC3	165	98.6144	1.3852	0.0004	n.d.	n.d.	n.d.	n.d.	n.d.	71	-66.3	-30.1	8.7	-295.1	-213.5	sI	
20	Seep13	VER11-01	2011St13GC3	182	98.3391	1.6603	0.0006	n.d.	n.d.	n.d.	n.d.	n.d.	59	-66.4	-29.9	9.4	-296.0	-211.4	sI	
20	Seep13	VER11-01	2011St13GC3	200	98.6083	1.3914	0.0003	n.d.	n.d.	n.d.	n.d.	n.d.	71	-67.1	-28.9	12.3	-294.3	-216.4	sI	
20	Seep13	VER13-03	2013St37GC3	68	99.3473	0.6526	0.0000	n.d.	n.d.	n.d.	n.d.	n.d.	152	-65.3	-25.1	n.d.	-285.7	-194.2	sI	
20	Seep13	VER13-03	2013St37GC3	78	98.9343	1.0653	0.0003	n.d.	0.0000	n.d.	n.d.	n.d.	93	-65.9	-27.9	n.d.	-290.9	-197.7	sI	
20	Seep13	VER13-03	2013St37GC3	83	98.2264	1.7734	0.0000	n.d.	0.0000	0.0002	n.d.	n.d.	55	-65.8	-26.7	n.d.	-287.6	-203.0	sI	
20	Seep13	VER13-03	2013St37GC3	85	98.9575	1.0421	0.0002	n.d.	0.0001	0.0000	n.d.	n.d.	95	-66.1	-28.4	n.d.	-286.2	-224.9	sI	
20	Seep13	VER13-03	2013St37GC3	110	99.3385	0.6612	0.0002	n.d.	0.0000	n.d.	n.d.	n.d.	150	-65.2	-26.9	n.d.	-284.5	-222.8	sI	
20	Seep13	VER13-03	2013St37GC3	112	99.2406	0.7585	0.0005	0.0004	0.0000	0.0000	n.d.	n.d.	131	-65.9	-27.7	n.d.	-289.0	-197.8	sI	
21	Krest	VER11-01	2011St14GC1	205	98.2410	1.7587	0.0003	n.d.	n.d.	n.d.	n.d.	n.d.	56	-68.2	-32.9	7.3	-288.7	-210.8	sI	
21	Krest	VER11-01	2011St14GC1	210	98.5772	1.4223	0.0006	n.d.	n.d.	n.d.	n.d.	n.d.	69	-67.9	-32.2	7.6	-286.0	-211.4	sI	
22	Kukuy K-3	VER12-03	2012St9GC1	211	99.4741	0.5242	0.0017	n.d.	n.d.	n.d.	n.d.	n.d.	189	-64.2	-25.6	-8.4	-310.3	-205.5	sI	
22	Kukuy K-3	VER12-03	2012St9GC1	253	99.7321	0.2584	0.0016	n.d.	0.0078	n.d.	n.d.	n.d.	383	-64.8	-25.1	-32.9	n.d.	n.d.	sI	
22	Kukuy K-3	VER12-03	2012St9GC6	101	98.4778	1.5182	0.0034	0.0004	0.0001	0.0001	n.d.	n.d.	65	-59.9	-23.1	-6.8	-315.3	-219.7	sI	
22	Kukuy K-3	VER12-03	2012St9GC6	118	99.1988	0.7998	0.0014	n.d.	n.d.	n.d.	n.d.	n.d.	124	-63.6	-25.9	-11.9	-308.7	-212.8	sI	
22	Kukuy K-3	VER12-03	2012St9GC6	118	n.d.	n.d.	n.d.	n.d.	n.d.	n.d.	n.d.	n.d.	n.d.	n.d.	-25.6	-11.6	n.d.	n.d.	sI	
22	Kukuy K-3	VER12-03	2012St9GC6	120	99.2236	0.7755	0.0008	0.0001	0.0000	n.d.	n.d.	n.d.	128	-64.8	-26.0	-11.8	-315.5	-208.4	sI	
22	Kukuy K-3	VER12-03	2012St9GC7	153	99.0886	0.9076	0.0030	0.0006	0.0001	0.0001	n.d.	n.d.	109	-64.3	-24.7	-7.1	-314.7	-206.2	sI	
22	Kukuy K-3	VER12-03	2012St9GC7	203	n.d.	n.d.	n.d.	n.d.	n.d.	n.d.	n.d.	n.d.	n.d.	n.d.	-25.2	-7.6	n.d.	n.d.	sI	
22	Kukuy K-3	VER12-03	2012St9GC7	205	99.7043	0.2940	0.0016	0.0001	0.0000	n.d.	n.d.	n.d.	337	-66.1	-25.6	-8.2	-313.8	-203.6	sI	
22	Kukuy K-3	VER12-03	2012St9GC7	205	n.d.	n.d.	n.d.	n.d.	n.d.	n.d.	n.d.	n.d.	n.d.	n.d.	-23.7	-6.5	n.d.	n.d.	sI	
22	Kukuy K-3	VER12-03	2012St9GC7	211	99.3516	0.6435	0.0049	n.d.	n.d.	n.d.	n.d.	n.d.	153	-64.7	-24.7	-17.2	-303.0	-210.6	sI	
22	Kukuy K-3	VER12-03	2012St9GC10	90	98.0638	1.9281	0.0070	0.0008	0.0001	0.0002	0.0001	n.d.	51	-59.7	-23.0	-5.4	-308.1	-204.6	sI	
22	Kukuy K-3	VER12-03	2012St9GC10	90	98.2524	1.7414	0.0051	0.0009	0.0002	0.0001	n.d.	n.d.	56	-60.5	-22.3	-5.1	-309.4	-204.7	sI	
22	Kukuy K-3	VER12-03	2012St9GC10	110	98.5392	1.4578	0.0030	n.d.	n.d.	n.d.	n.d.	n.d.	67	-60.8	-25.6	-6.2	-300.9	-203.4	sI	
22	Kukuy K-3	VER12-03	2012St9GC10	unknown	n.d.	n.d.	n.d.	n.d.	n.d.	n.d.	n.d.	n.d.	n.d.	n.d.	-24.5	-7.1	n.d.	n.d.	sI	
22	Kukuy K-3	VER12-03	2012St9GC10	unknown	n.d.	n.d.	n.d.	n.d.	n.d.	n.d.	n.d.	n.d.	n.d.	n.d.	-23.4	-6.4	n.d.	n.d.	sI	
22	Kukuy K-3	VER12-03	2012St9GC13	285	85.9845	13.4099	0.5128	0.0646	0.0214	0.0067	n.d.	n.d.	6	-44.1	-25.4	-5.1	-270.0	-204.5	sII	
22	Kukuy K-3	VER12-03	2012St9GC13	292	88.7791	10.7646	0.3835	0.0469	0.0131	0.0095	0.0031	n.d.	8	-44.6	-23.2	-5.1	-280.9	-206.4	sII	

Table S2 (continue)

No.	Site	Cruise	Core	Sample Depth [cmblf]	CH_4 [%]	C_2H_6 [%]	C_3H_8 [%]	i-C_4H_{10} [%]	n-C_4H_{10} [%]	neo-C_5H_{12} [%]	i-C_5H_{12} [%]	n-C_5H_{12} [%]	$C_1/(C_2+C_3)$	CH_4 $\delta^{13}C$ [‰VPDB]	C_2H_6 $\delta^{13}C$ [‰VPDB]	C_3H_8 $\delta^{13}C$ [‰VPDB]	CH_4 δ^2H [‰VSMOW]	C_2H_6 δ^2H [‰VSMOW]	Crystallographic Structure	note
					Molecular Composition									Isotopic Composition						
23	Krasny Yar	VER12-03	2012St19GC2	6	99.9892	0.0107	0.0001	n.d.	n.d.	n.d.	n.d.	n.d.	9260	-66.6	-66.9	-28.4	-318.3	-258.7	sI	
23	Krasny Yar	VER12-03	2012St19GC2	45	99.9956	0.0043	0.0001	n.d.	n.d.	n.d.	n.d.	n.d.	22520	-66.5	-65.4	-39.5	-315.7	n.d.	sI	
23	Krasny Yar	VER12-03	2012St19GC2	47	99.9933	0.0066	0.0001	n.d.	n.d.	n.d.	n.d.	n.d.	14825	-66.7	-66.9	-31.6	-316.2	-248.3	sI	
23	Krasny Yar	VER12-03	2012St19GC2	89	99.9912	0.0087	0.0001	n.d.	n.d.	n.d.	n.d.	n.d.	11361	-66.8	-66.4	n.d.	-317.7	n.d.	sI	
23	Krasny Yar	VER12-03	2012St19GC2	105	99.9896	0.0102	0.0002	n.d.	n.d.	n.d.	n.d.	n.d.	9641	-66.5	-67.2	-37.6	-318.8	-245.9	sI	
23	Krasny Yar	VER12-03	2012St19GC2	129	99.9887	0.0111	0.0001	n.d.	n.d.	n.d.	n.d.	n.d.	8887	-67.0	-63.7	-41.9	-318.6	-250.2	sI	
23	Krasny Yar	VER12-03	2012St19GC2	130	99.9896	0.0103	0.0001	n.d.	n.d.	n.d.	n.d.	n.d.	9585	-64.7	-64.8	n.d.	-312.3	-251.4	sI	
23	Krasny Yar	VER12-03	2012St19GC2	150	99.9911	0.0081	0.0005	0.0001	0.0002	n.d.	n.d.	n.d.	11657	-66.2	-65.1	-40.0	-315.2	n.d.	sI	
23	Krasny Yar	VER12-03	2012St19GC3	15	99.9925	0.0074	0.0002	n.d.	n.d.	n.d.	n.d.	n.d.	13275	-65.9	-66.6	n.d.	-315.8	-255.4	sI	
23	Krasny Yar	VER12-03	2012St19GC3	100	99.9931	0.0067	0.0002	n.d.	n.d.	n.d.	n.d.	n.d.	14495	-66.6	-67.2	-40.0	-318.5	-262.7	sI	
23	Krasny Yar	VER12-03	2012St19GC3	unknown	99.9916	0.0083	0.0001	n.d.	n.d.	n.d.	n.d.	n.d.	11864	-66.9	-66.7	-38.9	-318.8	-244.4	sI	
23	Krasny Yar	VER12-03	2012St19GC3	unknown	99.9932	0.0066	0.0002	n.d.	n.d.	n.d.	n.d.	n.d.	14739	-66.9	-66.2	-36.0	-316.8	n.d.	sI	
23	Krasny Yar	VER12-03	2012St19GC3	unknown	99.9124	0.0858	0.0018	n.d.	n.d.	n.d.	n.d.	n.d.	1141	n.d.	-63.0	-34.6	n.d.	n.d.	sI	
23	Krasny Yar	VER12-03	2012St19GC5	61	99.9915	0.0084	0.0001	n.d.	n.d.	n.d.	n.d.	n.d.	11742	-66.9	-66.5	-27.9	-317.7	-241.3	sI	
23	Krasny Yar	VER12-03	2012St19GC5	95	99.9901	0.0098	0.0001	n.d.	n.d.	n.d.	n.d.	n.d.	10098	-65.7	-70.0	-42.3	-309.1	n.d.	sI	
23	Krasny Yar	VER12-03	2012St19GC5	unknown	99.9912	0.0087	0.0001	n.d.	n.d.	n.d.	n.d.	n.d.	11389	-66.8	-66.5	-35.6	-320.7	-243.0	sI	
23	Krasny Yar	VER12-03	2012St19GC5	unknown	99.9896	0.0103	0.0001	n.d.	n.d.	n.d.	n.d.	n.d.	9621	-67.0	-66.8	-32.6	-320.9	-242.1	sI	
23	Krasny Yar	VER12-03	2012St19GC7	4	99.9899	0.0100	0.0001	n.d.	n.d.	n.d.	n.d.	n.d.	9926	-67.2	-66.6	-34.4	-319.1	-262.0	sI	
23	Krasny Yar	VER12-03	2012St19GC7	85	99.9904	0.0095	0.0001	n.d.	n.d.	n.d.	n.d.	n.d.	10393	-66.6	-66.5	-40.0	-320.3	-254.0	sI	
23	Krasny Yar	VER12-03	2012St19GC7	132	99.9935	0.0061	0.0004	n.d.	n.d.	n.d.	n.d.	n.d.	15384	-65.3	-65.3	-25.5	-314.0	n.d.	sI	
23	Krasny Yar	VER12-03	2012St19GC7	153	99.9902	0.0097	0.0001	n.d.	n.d.	n.d.	n.d.	n.d.	10191	-66.5	-66.4	-40.2	-319.1	-262.9	sI	
23	Krasny Yar	VER12-03	2012St19GC7	197	99.9870	0.0129	0.0001	n.d.	n.d.	n.d.	n.d.	n.d.	7706	-66.3	-65.3	n.d.	-316.2	n.d.	sI	
23	Krasny Yar	VER13-03	2013St3GC2	30	99.9912	0.0077	0.0001	0.0011	0.0000	0.0000	0.0000	n.d.	12951	-66.5	-66.3	n.d.	-311.7	-258.3	sI	
23	Krasny Yar	VER13-03	2013St3GC3	60	100.0000	0.0000	0.0000	n.d.	n.d.	n.d.	n.d.	n.d.	3069907	-65.7	n.d.	n.d.	-310.7	n.d.	sI	
23	Krasny Yar	VER13-03	2013St3GC3	125	100.0000	0.0000	0.0000	n.d.	n.d.	n.d.	n.d.	n.d.	4391384	-66.3	n.d.	n.d.	-305.0	n.d.	sI	
24	Kukuy K-pockmark	VER12-03	2012St9GC14	48	88.0804	11.2516	0.1176	0.0206	0.0032	0.5263	0.0003	n.d.	8	-48.9	-23.5	2.4	-296.4	-208.4	sII	
24	Kukuy K-pockmark	VER12-03	2012St9GC14	48	n.d.	n.d.	n.d.	n.d.	n.d.	n.d.	n.d.	n.d.	n.d.	n.d.	-27.6	5.8	n.d.	n.d.	sII	
24	Kukuy K-pockmark	VER12-03	2012St9GC14	52	86.5032	13.3835	0.0697	0.0069	0.0014	0.0352	0.0001	n.d.	6	-50.3	-23.1	-2.7	-290.8	-209.8	sII	
24	Kukuy K-pockmark	VER12-03	2012St9GC14	80	87.5492	12.3197	0.0933	0.0104	0.0019	0.0252	0.0003	n.d.	7	-50.4	-23.0	-3.5	-291.8	-209.1	sII	
24	Kukuy K-pockmark	VER12-03	2012St9GC14	80	87.8995	11.9413	0.1158	0.0147	0.0023	0.0260	0.0006	n.d.	7	-48.4	-24.8	-3.3	-288.9	-208.1	sII	
24	Kukuy K-pockmark	VER13-03	2013St24GC2	230	96.8203	3.1725	0.0049	0.0007	0.0003	0.0009	0.0003	0.0001	30	-54.0	-25.5	1.7	-289.2	n.d.	sI	
24	Kukuy K-pockmark	VER13-03	2013St24GC2	238	97.7468	2.2485	0.0039	0.0001	0.0003	0.0001	0.0003	n.d.	43	-53.5	-25.8	3.1	-290.6	-201.8	sI	
24	Kukuy K-pockmark	VER13-03	2013St24GC8	70	98.1151	1.8739	0.0062	0.0026	0.0005	0.0004	0.0013	0.0000	52	-57.9	-26.1	-5.6	-291.0	-234.8	sI	
24	Kukuy K-pockmark	VER13-03	2013St24GC8	75	98.6973	1.2937	0.0040	0.0017	0.0003	0.0002	0.0028	n.d.	76	-57.5	-24.9	-2.4	-290.9	-189.8	sI	
24	Kukuy K-pockmark	VER13-03	2013St24GC8	85	98.6848	1.3057	0.0064	0.0010	0.0004	0.0002	0.0016	n.d.	75	-58.1	-25.6	4.8	-296.1	-205.1	sI	
24	Kukuy K-pockmark	VER13-03	2013St24GC8	125	98.5986	1.3909	0.0069	0.0017	0.0003	0.0003	0.0012	n.d.	71	-58.1	-25.5	6.0	-296.0	n.d.	sI	
24	Kukuy K-pockmark	VER13-03	2013St24GC8	130	98.9326	1.0556	0.0058	0.0031	0.0004	0.0009	0.0013	0.0004	93	-57.7	-24.7	-4.9	-293.1	-193.8	sI	
25	Ukhan	VER12-03	2012St15GC2	90	n.d.	n.d.	n.d.	n.d.	n.d.	n.d.	n.d.	n.d.	n.d.	n.d.	-48.3	-29.2	n.d.	-250.4	sI	
25	Ukhan	VER12-03	2012St15GC2	90	n.d.	n.d.	n.d.	n.d.	n.d.	n.d.	n.d.	n.d.	n.d.	n.d.	n.d.	n.d.	n.d.	-258.9	sI	
25	Ukhan	VER12-03	2012St15GC2	90	99.9434	0.0564	0.0002	n.d.	n.d.	n.d.	n.d.	n.d.	1766	-66.8	-48.5	-29.5	-288.7	-256.1	sI	
25	Ukhan	VER12-03	2012St15GC2	90	99.9587	0.0408	0.0005	n.d.	n.d.	n.d.	n.d.	n.d.	2419	-68.1	-49.5	-27.9	-298.4	-260.3	sI	
25	Ukhan	VER12-03	2012St15GC3	150	99.9752	0.0245	0.0003	n.d.	n.d.	n.d.	n.d.	n.d.	4037	-66.0	-55.5	n.d.	-291.8	n.d.	sI	

Table S2 (continue)

No.	Site	Cruise	Core	Sample Depth [cmblf]	CH$_4$ [%]	C$_2$H$_6$ [%]	C$_3$H$_8$ [%]	i-C$_4$H$_{10}$ [%]	n-C$_4$H$_{10}$ [%]	neo-C$_5$H$_{12}$ [%]	i-C$_5$H$_{12}$ [%]	n-C$_5$H$_{12}$ [%]	C$_1$/(C$_2$+C$_3$)	CH$_4$ δ^{13}C [‰VPDB]	C$_2$H$_6$ δ^{13}C [‰VPDB]	C$_3$H$_8$ δ^{13}C [‰VPDB]	CH$_4$ δ^{2}H [‰VSMOW]	C$_2$H$_6$ δ^{2}H [‰VSMOW]	Crystallographic Structure	note
					Molecular Composition									Isotopic Composition						
26	Unshuy	VER12-03	2012St14GC3	206	99.8552	0.1448	0.0000	n.d.	n.d.	n.d.	n.d.	n.d.	689	-67.2	-51.2	n.d.	n.d.	n.d.	sI	
26	Unshuy	VER12-03	2012St14GC3	243	99.8376	0.1624	0.0000	n.d.	n.d.	n.d.	n.d.	n.d.	615	-69.4	-51.8	n.d.	-288.5	-239.0	sI	
26	Unshuy	VER12-03	2012St14GC3	254	99.9115	0.0885	0.0001	n.d.	n.d.	n.d.	n.d.	n.d.	1128	-67.9	-51.0	-30.5	-275.6	-241.2	sI	
26	Unshuy	VER12-03	2012St14GC3	254	99.9019	0.0980	0.0001	n.d.	n.d.	n.d.	n.d.	n.d.	1018	-69.2	-51.4	-23.1	-284.6	-239.2	sI	
26	Unshuy	VER12-03	2012St14GC3	254	n.d.	n.d.	n.d.	n.d.	n.d.	n.d.	n.d.	n.d.	n.d.	n.d.	-51.0	-25.7	n.d.	-246.5	sI	
26	Unshuy	VER14-03	2014St16GC1	408	99.9049	0.0947	0.0005	n.d.	n.d.	n.d.	n.d.	n.d.	1050	-68.1	-50.8	-24.6	-283.7	-280.0	sI	
27	Krasny Yar-2	VER13-03	2013St1GC1	40	99.9987	0.0013	0.0000	n.d.	n.d.	n.d.	n.d.	n.d.	74185	-65.7	n.d.	n.d.	-317.1	n.d.	sI	
28	Krasny Yar-3	VER13-03	2013St2GC1	30	n.d.	n.d.	n.d.	n.d.	n.d.	n.d.	n.d.	n.d.	n.d.	-65.6	n.d.	n.d.	-308.7	n.d.	sI	
29	Kukuy K-5	VER14-03	2014St2GC2	146	99.3105	0.6879	0.0013	0.0002	0.0000	0.0001	n.d.	n.d.	144	-63.8	-26.1	-10.5	-318.5	-223.2	sI	
29	Kukuy K-5	VER14-03	2014St2GC2	160	99.3538	0.6447	0.0012	0.0002	0.0000	0.0001	n.d.	n.d.	154	-64.0	-26.3	2.1	-318.1	-222.4	sI	
29	Kukuy K-5	VER14-03	2014St2GC2	172	99.4210	0.5772	0.0014	0.0002	0.0000	0.0002	n.d.	n.d.	172	-64.1	-25.5	-12.7	-318.0	-216.3	sI	
29	Kukuy K-5	VER14-03	2014St2GC3	250	99.3518	0.6478	0.0003	0.0000	0.0001	n.d.	n.d.	n.d.	153	-65.1	-25.3	-10.7	-313.2	-213.7	sI	
29	Kukuy K-5	VER14-03	2014St2GC3	260	99.4617	0.5381	0.0001	0.0001	0.0000	n.d.	n.d.	n.d.	185	-65.0	n.d.	n.d.	-313.6	-198.7	sI	
29	Kukuy K-5	VER14-03	2014St2GC3	270	99.3742	0.6255	0.0002	0.0000	0.0001	n.d.	n.d.	n.d.	159	-64.9	-25.2	-4.4	-313.1	-222.8	sI	
29	Kukuy K-5	VER14-03	2014St2GC3	280	99.4995	0.4987	0.0013	0.0004	0.0001	n.d.	n.d.	n.d.	199	-65.0	-25.3	-14.2	-314.1	-222.6	sI	
29	Kukuy K-5	VER14-03	2014St2GC3	290	99.5035	0.4961	0.0002	0.0001	0.0001	n.d.	n.d.	n.d.	200	-64.8	-25.2	-22.7	-312.5	-214.7	sI	
29	Kukuy K-5	VER14-03	2014St2GC3	295	99.4796	0.5200	0.0003	0.0001	0.0000	n.d.	n.d.	n.d.	191	-64.6	-26.0	n.d.	-319.0	-223.2	sI	
29	Kukuy K-5	VER14-03	2014St2GC4	183	99.6452	0.3547	0.0000	0.0001	0.0000	n.d.	n.d.	n.d.	281	-64.7	n.d.	n.d.	-315.3	n.d.	sI	
29	Kukuy K-5	VER14-03	2014St2GC4	193	99.3033	0.6962	0.0004	0.0001	0.0001	n.d.	n.d.	n.d.	143	-64.4	-25.5	-12.6	-314.2	-218.7	sI	
29	Kukuy K-5	VER14-03	2014St2GC4	210	99.3343	0.6651	0.0004	0.0001	0.0001	n.d.	n.d.	n.d.	149	-64.4	-25.5	-12.2	-314.9	-226.0	sI	
29	Kukuy K-5	VER14-03	2014St2GC4	250	99.6439	0.3556	0.0003	0.0001	0.0001	n.d.	n.d.	n.d.	280	-64.6	n.d.	-9.1	-312.4	n.d.	sI	
29	Kukuy K-5	VER14-03	2014St2GC4	265	99.6157	0.3838	0.0004	0.0000	0.0001	n.d.	n.d.	n.d.	259	-65.0	-26.1	-9.9	-313.3	-217.7	sI	
29	Kukuy K-5	VER14-03	2014St2GC4	275	99.5665	0.4331	0.0003	0.0001	0.0000	0.0001	n.d.	n.d.	230	-64.7	-25.9	n.d.	-318.7	-224.6	sI	
30	St. Petersburg-2	VER14-03	2014St15GC2	241	99.4748	0.5234	0.0018	n.d.	n.d.	n.d.	n.d.	n.d.	189	-66.5	-32.0	n.d.	-292.0	n.d.	sI	
30	St. Petersburg-2	VER14-03	2014St15GC2	242	99.6298	0.3670	0.0022	0.0009	0.0000	n.d.	n.d.	n.d.	270	-66.2	-30.3	-4.9	-292.5	-235.5	sI	
30	St. Petersburg-2	VER14-03	2014St15GC6	78	99.8124	0.1869	0.0006	0.0002	n.d.	n.d.	n.d.	n.d.	533	-66.2	0.0	-4.4	-285.6	n.d.	sI	
30	St. Petersburg-2	VER14-03	2014St15GC6	93	99.7089	0.2869	0.0037	0.0004	0.0001	n.d.	n.d.	n.d.	343	-66.4	-29.5	-4.8	-286.5	-231.5	sI	
30	St. Petersburg-2	VER14-03	2014St15GC6	103	99.5972	0.4011	0.0018	n.d.	n.d.	n.d.	n.d.	n.d.	247	-66.4	-30.5	-9.4	-292.4	-233.8	sI	
30	St. Petersburg-2	VER14-03	2014St15GC6	213	99.4727	0.5260	0.0013	0.0000	0.0000	n.d.	n.d.	n.d.	189	-65.6	-30.1	-15.7	-293.7	-231.2	sI	
30	St. Petersburg-2	VER14-03	2014St15GC6	216	99.6246	0.3547	0.0205	0.0001	0.0000	n.d.	n.d.	n.d.	266	-66.3	-30.2	-9.6	-292.4	-233.9	sI	
30	St. Petersburg-2	VER14-03	2014St15GC6	219	99.6338	0.3632	0.0028	0.0001	0.0000	n.d.	n.d.	n.d.	272	-66.4	-30.3	-5.6	-291.9	-233.5	sI	
30	St. Petersburg-2	VER14-03	2014St15GC6	221	99.6411	0.3578	0.0010	0.0000	0.0000	n.d.	n.d.	n.d.	278	-66.5	-30.2	-11.8	-293.2	-234.7	sI	
30	St. Petersburg-2	VER14-03	2014St15GC8	197	99.3994	0.6000	0.0006	n.d.	n.d.	n.d.	n.d.	n.d.	165	-66.6	-28.5	-3.2	-289.5	-229.4	sI	
30	St. Petersburg-2	VER14-03	2014St15GC8	216	99.8037	0.1914	0.0046	0.0003	0.0000	n.d.	n.d.	n.d.	509	-66.3	-29.6	-3.4	-285.0	-235.9	sI	
30	St. Petersburg-2	VER14-03	2014St15GC8	222	99.4234	0.5755	0.0011	n.d.	n.d.	n.d.	n.d.	n.d.	172	-66.4	-28.7	0.3	-288.2	-232.8	sI	
30	St. Petersburg-2	VER14-03	2014St15GC8	252	99.5474	0.4489	0.0033	0.0004	0.0001	n.d.	n.d.	n.d.	220	-66.0	-30.9	-5.2	-292.0	-234.4	sI	
31	Khoboy	VER14-03	2014St5GC4	212	99.9776	0.0224	0.0000	n.d.	n.d.	n.d.	n.d.	n.d.	4459	-65.0	-33.4	n.d.	-316.6	n.d.	sI	
31	Khoboy	VER14-03	2014St5GC4	223	99.9470	0.0527	0.0003	n.d.	n.d.	n.d.	n.d.	n.d.	1885	-64.9	-43.9	-28.5	-321.9	-272.6	sI	
31	Khoboy	VER14-03	2014St5GC5	319	99.9635	0.0363	0.0000	n.d.	0.0001	n.d.	n.d.	n.d.	2748	-65.6	-39.0	n.d.	-317.3	-234.8	sI	

Table S2 (continue)

No.	Site	Cruise	Core	Sample Depth [cmblf]	Molecular Composition CH_4 [%]	C_2H_6 [%]	C_3H_8 [%]	$i\text{-}C_4H_{10}$ [%]	$n\text{-}C_4H_{10}$ [%]	$neo\text{-}C_5H_{12}$ [%]	$i\text{-}C_5H_{12}$ [%]	$n\text{-}C_5H_{12}$ [%]	$C_1/(C_2+C_3)$	Isotopic Composition CH_4 $\delta^{13}C$ [‰VPDB]	C_2H_6 $\delta^{13}C$ [‰VPDB]	C_3H_8 $\delta^{13}C$ [‰VPDB]	CH_4 δ^2H [‰VSMOW]	C_2H_6 δ^2H [‰VSMOW]	Crystallographic Structure	note
31	Khoboy	VER14-03	2014St5GC5	360	99.9557	0.0441	0.0001	n.d.	0.0001	n.d.	n.d.	n.d.	2264	-65.6	-40.6	-38.1	-316.7	-249.3	sI	
31	Khoboy	VER14-03	2014St5GC5	420	99.9625	0.0374	0.0001	n.d.	0.0000	n.d.	n.d.	n.d.	2668	-65.2	-41.8	n.d.	-321.4	-265.8	sI	
31	Khoboy	VER14-03	2014St5GC7	433	99.8990	0.1009	0.0000	n.d.	0.0001	n.d.	n.d.	n.d.	990	-65.5	-40.5	n.d.	-317.8	-242.4	sI	
31	Khoboy	VER14-03	2014St5GC7	450	99.9496	0.0503	0.0000	n.d.	0.0000	n.d.	n.d.	n.d.	1986	-65.6	-36.0	n.d.	-317.9	-212.3	sI	
31	Khoboy	VER14-03	2014St5GC7	460	99.9205	0.0794	0.0001	n.d.	0.0000	n.d.	n.d.	n.d.	1257	-65.1	-41.5	n.d.	-321.9	-268.8	sI	
31	Khoboy	VER14-03	2014St5GC7	460	99.9472	0.0524	0.0004	n.d.	n.d.	n.d.	n.d.	n.d.	1893	-65.3	-42.3	-29.2	-321.0	-275.7	sI	
31	Khoboy	VER14-03	2014St5GC9	128	99.9668	0.0331	0.0000	n.d.	0.0001	n.d.	n.d.	n.d.	3016	-65.4	-39.8	n.d.	-321.3	-219.2	sI	
31	Khoboy	VER14-03	2014St5GC9	148	99.9444	0.0555	0.0001	n.d.	0.0000	n.d.	n.d.	n.d.	1799	-64.9	-44.2	n.d.	-325.1	-276.4	sI	
31	Khoboy	VER14-03	2014St5GC10	215	99.9603	0.0395	0.0001	n.d.	0.0001	n.d.	n.d.	n.d.	2522	-65.9	-43.3	-43.7	-319.3	-254.8	sI	
31	Khoboy	VER14-03	2014St5GC10	260	99.9822	0.0178	0.0000	n.d.	0.0001	n.d.	n.d.	n.d.	5625	-65.5	n.d.	n.d.	-316.9	-227.1	sI	
31	Khoboy	VER14-03	2014St5GC10	300	99.9721	0.0275	0.0002	n.d.	0.0001	n.d.	n.d.	n.d.	3601	-65.7	-43.2	-38.1	-315.7	-262.6	sI	
31	Khoboy	VER14-03	2014St5GC10	330	99.9672	0.0326	0.0002	n.d.	0.0000	n.d.	n.d.	n.d.	3046	-65.4	-44.1	-27.0	-322.8	-278.7	sI	
31	Khoboy	VER14-03	2014St5GC11	235	99.9456	0.0543	0.0000	n.d.	n.d.	n.d.	n.d.	n.d.	1838	-65.2	-42.9	n.d.	-323.7	-277.4	sI	
31	Khoboy	VER15-03	2015St12GC10	20	99.9510	0.0490	0.0000	n.d.	n.d.	n.d.	n.d.	n.d.	2040	-65.9	-43.6	n.d.	-319.1	-249.3	sI	
31	Khoboy	VER15-03	2015St12GC10	45	99.9479	0.0519	0.0002	n.d.	0.0000	n.d.	n.d.	n.d.	1920	-65.9	-43.2	-27.8	-319.2	-254.8	sI	
32	AkademRidge	VER14-03	2014St9GC4	97	99.9750	0.0230	0.0020	n.d.	n.d.	n.d.	n.d.	n.d.	4003	-65.6	-55.4	-26.8	-315.1	-305.1	sI	
32	AkademRidge	VER14-03	2014St9GC4	130	99.9865	0.0115	0.0020	0.0000	n.d.	n.d.	n.d.	n.d.	7397	-66.0	-55.8	-27.0	-313.0	-287.5	sI	
32	AkademRidge	VER14-03	2014St9GC7	50	99.9709	0.0273	0.0012	0.0003	0.0001	n.d.	n.d.	n.d.	3499	-66.3	-54.9	-28.9	-308.9	-289.7	sI	
32	AkademRidge	VER14-03	2014St9GC7	95	99.9723	0.0256	0.0016	0.0005	0.0001	n.d.	n.d.	n.d.	3680	-66.3	-54.9	-29.0	-308.2	-291.3	sI	
32	AkademRidge	VER14-03	2014St9GC7	110	99.9737	0.0228	0.0026	0.0007	0.0002	n.d.	n.d.	n.d.	3936	-66.0	-55.5	-28.9	-314.4	-302.1	sI	
32	AkademRidge	VER14-03	2014St9GC10	21	99.9824	0.0167	0.0006	0.0001	0.0002	n.d.	n.d.	n.d.	5790	-65.9	n.d.	-23.3	-308.5	-248.4	sI	
32	AkademRidge	VER14-03	2014St9GC10	25	99.9753	0.0231	0.0012	0.0001	0.0002	n.d.	n.d.	n.d.	4116	-66.0	-53.6	-25.8	-309.9	-291.8	sI	
32	AkademRidge	VER14-03	2014St9GC10	97	99.9701	0.0282	0.0014	0.0002	0.0001	n.d.	n.d.	n.d.	3376	-66.3	-54.9	-27.2	-311.3	-290.0	sI	
32	AkademRidge	VER14-03	2014St9GC10	295	99.9747	0.0228	0.0020	0.0005	n.d.	n.d.	n.d.	n.d.	4030	-65.9	-54.7	-24.9	-314.3	-304.6	sI	
32	AkademRidge	VER14-03	2014St9GC13	75	99.9683	0.0305	0.0007	0.0005	0.0000	n.d.	n.d.	n.d.	3200	-65.8	-56.2	-26.8	-318.5	-312.6	sI	
32	AkademRidge	VER14-03	2014St9GC13	79	99.9742	0.0248	0.0010	0.0000	0.0000	n.d.	n.d.	n.d.	3877	-65.8	-55.7	-26.9	-316.3	-310.3	sI	
32	AkademRidge	VER14-03	2014St9GC13	82	99.9713	0.0281	0.0005	0.0001	0.0000	n.d.	n.d.	n.d.	3492	-66.0	-56.2	-26.4	-317.7	-313.6	sI	
32	AkademRidge	VER14-03	2014St9GC13	86	99.9737	0.0256	0.0006	0.0001	0.0000	n.d.	n.d.	n.d.	3817	-65.9	-56.2	-25.1	-318.1	-311.6	sI	
32	AkademRidge	VER14-03	2014St9GC13	90	99.9735	0.0260	0.0005	0.0001	0.0000	n.d.	n.d.	n.d.	3779	-65.9	-51.3	n.d.	-316.9	n.d.	sI	
32	AkademRidge	VER14-03	2014St9GC13	94	99.9727	0.0265	0.0007	0.0001	0.0000	n.d.	n.d.	n.d.	3677	-65.8	-52.9	n.d.	-317.4	n.d.	sI	
32	AkademRidge	VER14-03	2014St9GC13	98	99.9707	0.0287	0.0005	0.0001	0.0000	n.d.	n.d.	n.d.	3429	-66.0	-53.2	-23.7	-317.1	n.d.	sI	
32	AkademRidge	VER14-03	2014St9GC14	333	99.9618	0.0376	0.0005	0.0000	0.0001	n.d.	n.d.	n.d.	2621	-66.6	-54.5	-26.9	-312.2	-287.9	sI	
32	AkademRidge	VER14-03	2014St9GC14	343	99.9679	0.0296	0.0020	0.0005	0.0000	n.d.	n.d.	n.d.	3168	-66.0	-55.0	-25.0	-315.5	-310.8	sI	
32	AkademRidge	VER14-03	2014St9GC15	468	99.9598	0.0396	0.0005	0.0002	n.d.	n.d.	n.d.	n.d.	2494	-66.9	-55.2	-29.2	-311.1	n.d.	sI	
32	AkademRidge	VER14-03	2014St9GC15	495	99.9610	0.0383	0.0007	n.d.	n.d.	n.d.	n.d.	n.d.	2566	-66.1	-55.6	-26.8	-317.2	-309.0	sI	
32	AkademRidge	VER14-03	2014St9GC16	432	99.9477	0.0490	0.0023	0.0009	0.0001	n.d.	n.d.	n.d.	1949	-65.3	-54.0	-24.3	n.d.	-305.1	sI	
32	AkademRidge	VER14-03	2014St9GC17	212	99.9644	0.0351	0.0005	n.d.	n.d.	n.d.	n.d.	n.d.	2811	-66.6	-55.9	-31.5	-312.3	-290.0	sI	
32	AkademRidge	VER14-03	2014St9GC17	264	99.9664	0.0323	0.0008	0.0006	0.0000	n.d.	n.d.	n.d.	3027	-65.8	-55.8	-30.4	-316.2	-318.8	sI	
32	AkademRidge	VER14-03	2014St9GC18	245	99.9836	0.0153	0.0011	n.d.	n.d.	n.d.	n.d.	n.d.	6109	-66.4	-55.2	-24.9	-310.8	-273.6	sI	
32	AkademRidge	VER14-03	2014St9GC18	285	99.9772	0.0222	0.0005	0.0000	0.0000	n.d.	n.d.	n.d.	4392	-66.1	-55.9	-24.3	-316.4	-303.9	sI	
32	AkademRidge	VER15-03	2015St13GC3t	53	99.9551	0.0419	0.0020	0.0008	0.0001	n.d.	n.d.	n.d.	2273	-66.2	-52.9	-23.7	-312.2	-277.6	sI	
32	AkademRidge	VER15-03	2015St13GC3t	85	99.9590	0.0373	0.0026	0.0009	0.0001	n.d.	n.d.	n.d.	2501	-66.1	-55.4	-27.8	-312.8	-286.1	sI	
32	AkademRidge	VER15-03	2015St13GC4	63	99.9718	0.0277	0.0004	0.0001	n.d.	n.d.	n.d.	n.d.	3559	-66.4	-55.6	-25.0	-312.1	-289.9	sI	

Table S2 (continue)

No.	Site	Cruise	Core	Sample Depth [cmblf]	CH_4 [%]	C_2H_6 [%]	C_3H_8 [%]	i-C_4H_{10} [%]	n-C_4H_{10} [%]	neo-C_5H_{12} [%]	i-C_5H_{12} [%]	n-C_5H_{12} [%]	$C_1/(C_2+C_3)$	CH_4 $\delta^{13}C$ [‰VPDB]	C_2H_6 $\delta^{13}C$ [‰VPDB]	C_3H_8 $\delta^{13}C$ [‰VPDB]	CH_4 δ^2H [‰VSMOW]	C_2H_6 δ^2H [‰VSMOW]	Crystallographic Structure	note
					Molecular Composition									Isotopic Composition						
32	AkademRidge	VER15-03	2015St13GC4	75	n.d.	n.d.	n.d.	n.d.	n.d.	n.d.	n.d.	n.d.	n.d.	-66.5	n.d.	n.d.	-311.0	n.d.	sI	
32	AkademRidge	VER15-03	2015St13GC4	77	99.9765	0.0224	0.0010	0.0001	n.d.	n.d.	n.d.	n.d.	4270	-66.4	-55.2	-26.9	-312.0	-283.0	sI	
32	AkademRidge	VER15-03	2015St13GC4	92	99.9777	0.0219	0.0003	0.0000	n.d.	n.d.	n.d.	n.d.	4496	-66.4	-55.2	-28.3	-311.9	-286.9	sI	
33	Barguzin	VER14-03	2014St12GC1	287	99.9445	0.0553	0.0003	0.0000	n.d.	n.d.	n.d.	n.d.	1800	-66.4	-63.2	-33.8	-311.7	-300.7	sI	
33	Barguzin	VER14-03	2014St12GC1	330	99.9429	0.0568	0.0003	0.0000	n.d.	n.d.	n.d.	n.d.	1751	-66.7	-63.5	-31.7	-311.3	-298.2	sI	
33	Barguzin	VER14-03	2014St12GC1	333	99.9435	0.0562	0.0002	0.0000	0.0000	n.d.	n.d.	n.d.	1770	-66.6	-63.7	-46.8	-310.4	-299.6	sI	
33	Barguzin	VER14-03	2014St12GC1	347	99.9411	0.0587	0.0002	0.0000	0.0000	n.d.	n.d.	n.d.	1697	-66.4	-63.5	-28.1	-310.5	-298.6	sI	
33	Barguzin	VER14-03	2014St12GC1	388	99.9581	0.0413	0.0005	0.0001	0.0000	n.d.	n.d.	n.d.	2391	-66.2	-63.5	-34.6	-307.4	-298.6	sI	
33	Barguzin	VER15-03	2015St14GC1	290	99.8825	0.1175	0.0001	n.d.	n.d.	n.d.	n.d.	n.d.	850	-66.7	-62.9	n.d.	-308.9	-270.1	sI	
33	Barguzin	VER15-03	2015St14GC1	293	99.9432	0.0566	0.0002	n.d.	n.d.	n.d.	n.d.	n.d.	1759	-67.1	-62.9	-37.3	-306.1	-270.4	sI	
33	Barguzin	VER15-03	2015St14GC1	295	99.9366	0.0632	0.0002	n.d.	n.d.	n.d.	n.d.	n.d.	1575	-66.9	-63.3	-37.0	-305.5	-271.7	sI	
34	Mamay	VER15-03	2015St20GC6	125	97.7948	2.2050	0.0003	n.d.	n.d.	n.d.	n.d.	n.d.	44	-53.6	-28.7	n.d.	-293.9	-227.0	sI	
34	Mamay	VER15-03	2015St20GC6	133	98.4916	1.5082	0.0002	n.d.	n.d.	n.d.	n.d.	n.d.	65	-53.8	-29.0	n.d.	-292.4	-229.5	sI	
34	Mamay	VER15-03	2015St20GC6	137	98.4636	1.5362	0.0002	n.d.	n.d.	n.d.	n.d.	n.d.	64	-53.8	-28.9	n.d.	-293.1	-227.7	sI	
34	Mamay	VER15-03	2015St20GC6	148	98.7195	1.2803	0.0002	n.d.	n.d.	n.d.	n.d.	n.d.	77	-53.9	-29.1	n.d.	-294.3	-229.3	sI	
34	Mamay	VER15-03	2015St20GC6	148	98.7078	1.2920	0.0002	n.d.	n.d.	n.d.	n.d.	n.d.	76	-53.7	-28.9	n.d.	-293.0	-222.5	sI	
34	Mamay	VER15-03	2015St20GC6	148	98.7527	1.2472	0.0002	n.d.	n.d.	n.d.	n.d.	n.d.	79	-53.9	-29.1	n.d.	-293.5	-224.5	sI	
34	Mamay	VER15-03	2015St20GC8	unknown	98.3270	1.6728	0.0002	0.0000	0.0001	n.d.	n.d.	n.d.	59	-53.9	-29.1	n.d.	-293.8	-229.2	sI	
34	Mamay	VER17-03	2017St16GC1	198	98.6260	1.3738	0.0001	n.d.	0.0000	0.0000	n.d.	n.d.	72	-54.4	-30.8	n.d.	-293.8	-216.7	sI	
34	Mamay	VER17-03	2017St16GC1	173	98.2809	1.7188	0.0001	0.0000	0.0000	0.0001	n.d.	n.d.	57	-54.2	-31.0	n.d.	-294.3	-217.3	sI	
35	Kamenny	VER15-03	2015St6GC2	246	99.9777	0.0220	0.0003	n.d.	n.d.	n.d.	n.d.	n.d.	4480	-66.6	-63.3	-37.1	-310.3	-279.2	sI	
35	Kamenny	VER15-03	2015St6GC2	248	99.9762	0.0234	0.0003	0.0000	0.0000	n.d.	n.d.	n.d.	4202	-66.8	-64.0	-37.5	-310.4	-280.1	sI	
35	Kamenny	VER15-03	2015St6GC2	251	99.9810	0.0186	0.0004	n.d.	n.d.	n.d.	n.d.	n.d.	5266	-66.6	-63.4	-39.9	-310.4	-275.8	sI	
35	Kamenny	VER15-03	2015St6GC2	256	99.9722	0.0275	0.0003	n.d.	n.d.	n.d.	n.d.	n.d.	3600	-66.7	-63.3	-41.6	-311.4	-279.1	sI	
36	Tonky	VER15-03	2015St8GC1	215	99.9993	0.0005	0.0000	n.d.	0.0002	n.d.	n.d.	n.d.	207371	-64.4	n.d.	n.d.	-284.5	n.d.	sI	
36	Tonky	VER15-03	2015St8GC5	75	99.9983	0.0016	0.0001	n.d.	n.d.	n.d.	n.d.	n.d.	57769	-65.4	n.d.	n.d.	-288.8	n.d.	sI	
37	Talanka	VER15-03	2015St10GC1	130	99.9715	0.0283	0.0002	0.0000	0.0000	n.d.	n.d.	n.d.	3508	-68.2	-67.6	-26.8	-303.3	-281.6	sI	
37	Talanka	VER15-03	2015St10GC1	139	99.9715	0.0283	0.0002	0.0000	0.0000	n.d.	n.d.	n.d.	3511	-68.2	-67.4	-37.7	-302.5	-278.5	sI	
37	Talanka	VER15-03	2015St10GC1	147	99.9708	0.0291	0.0001	0.0000	0.0000	n.d.	n.d.	n.d.	3429	-68.2	-67.1	-10.5	-302.4	-283.7	sI	
37	Talanka	VER15-03	2015St10GC1	150	99.9686	0.0313	0.0001	0.0000	0.0000	n.d.	n.d.	n.d.	3184	-68.0	-67.1	-45.1	-302.9	-278.8	sI	
38	Novosibirsk-2	VER16-03	2016St2GC1	90	99.9794	0.0203	0.0002	n.d.	0.0000	n.d.	n.d.	n.d.	4862	-67.7	-55.9	-28.8	-297.6	n.d.	sI	
38	Novosibirsk-2	VER16-03	2016St2GC1	95	99.9783	0.0214	0.0003	n.d.	0.0000	n.d.	n.d.	n.d.	4605	-67.9	-55.2	-33.0	-297.4	n.d.	sI	
38	Novosibirsk-2	VER16-03	2016St2GC1	100	99.9826	0.0170	0.0004	n.d.	0.0000	n.d.	n.d.	n.d.	5762	-67.9	-56.1	-26.6	-296.2	n.d.	sI	
38	Novosibirsk-2	VER16-03	2016St2GC1	107	99.9813	0.0183	0.0003	0.0000	0.0000	n.d.	n.d.	n.d.	5375	n.d.	n.d.	n.d.	n.d.	n.d.	sI	
38	Novosibirsk-2	VER16-03	2016St2GC1	107	99.9775	0.0223	0.0001	n.d.	0.0000	n.d.	n.d.	n.d.	4447	-68.0	-55.7	-35.1	-298.2	n.d.	sI	
39	Kedr	VER15-03	2015St1GC2	25	86.9179	13.0663	0.0084	0.0006	0.0004	0.0064	n.d.	n.d.	7	-46.0	-25.8	-8.5	-276.9	-212.8	sII	b)
39	Kedr	VER15-03	2015St1GC2	47	84.9494	14.7780	0.2095	0.0221	0.0020	0.0388	0.0003	n.d.	6	-45.5	-25.7	-9.5	-276.6	-225.5	sII	b)

Table S2 (continue)

No.	Site	Cruise	Core	Sample Depth [cmblf]	Molecular Composition CH_4 [%]	C_2H_6 [%]	C_3H_8 [%]	i-C_4H_{10} [%]	n-C_4H_{10} [%]	neo-C_5H_{12} [%]	i-C_5H_{12} [%]	n-C_5H_{12} [%]	$C_1/(C_2+C_3)$	Isotopic Composition CH_4 $\delta^{13}C$ [‰VPDB]	C_2H_6 $\delta^{13}C$ [‰VPDB]	C_3H_8 $\delta^{13}C$ [‰VPDB]	CH_4 δ^2H [‰VSMOW]	C_2H_6 δ^2H [‰VSMOW]	Crystallographic Structure	note
39	Kedr	VER15-03	2015St1GC2	53	86.8572	13.1287	0.0075	0.0005	0.0003	0.0059	n.d.	n.d.	7	-46.3	-25.7	-11.6	-276.2	-214.7	sII	b)
39	Kedr	VER15-03	2015St1GC8	132	85.0207	14.8395	0.1098	0.0085	0.0010	0.0204	0.0001	n.d.	6	-47.2	-25.9	-10.5	-276.9	-219.2	sII	b)
39	Kedr	VER15-03	2015St1GC8	145	85.0455	14.8417	0.0814	0.0069	0.0007	0.0237	0.0001	n.d.	6	-47.1	-25.7	-11.2	-276.7	-220.7	sII	b)
39	Kedr	VER15-03	2015St1GC8	158	85.0833	14.6817	0.1957	0.0198	0.0025	0.0168	0.0004	n.d.	6	-46.5	-25.9	-11.5	-275.8	-223.8	sII	b)
39	Kedr	VER15-03	2015St1GC8	163	84.3860	15.2569	0.3055	0.0269	0.0024	0.0218	0.0005	n.d.	5	-46.9	-25.9	-11.2	-274.2	-222.3	sII	b)
39	Kedr	VER15-03	2015St1GC8	177	85.2174	14.6019	0.1436	0.0140	0.0013	0.0216	0.0001	n.d.	6	-47.1	-26.0	-11.1	-276.4	-214.7	sII	b)
39	Kedr	VER15-03	2015St1GC8	195	84.3781	15.5596	0.0079	0.0004	0.0001	0.0540	n.d.	n.d.	5	-47.4	-25.3	-3.7	-276.0	-219.0	sII	b)
39	Kedr	VER15-03	2015St1GC10	42	94.5261	5.4608	0.0090	0.0003	0.0001	0.0036	0.0001	n.d.	17	-46.0	-25.5	-7.6	-274.2	-217.4	sI	b)
39	Kedr	VER15-03	2015St1GC10	50	92.8601	7.1120	0.0194	0.0016	0.0002	0.0068	n.d.	n.d.	13	-44.7	-25.8	-9.6	-272.8	-219.9	sI	b)
39	Kedr	VER15-03	2015St1GC10	65	95.1071	4.8889	0.0029	0.0002	0.0001	0.0008	0.0000	n.d.	19	-44.9	-25.8	-12.1	-273.7	-218.3	sI	b)
39	Kedr	VER15-03	2015St1GC11	124	96.8228	3.1758	0.0010	0.0000	0.0001	0.0003	0.0000	n.d.	30	-46.6	-25.6	-6.3	-275.1	-221.5	sI	b)
39	Kedr	VER15-03	2015St1GC11	149	96.9865	3.0060	0.0045	0.0002	0.0004	0.0024	n.d.	n.d.	32	-46.5	-25.4	-8.5	-274.2	-221.6	sI	b)
39	Kedr	VER15-03	2015St1GC11	185	96.7167	3.2795	0.0027	0.0001	0.0001	0.0009	n.d.	n.d.	29	-46.9	-25.6	-12.9	-274.6	-220.2	sI	b)
39	Kedr	VER15-03	2015St1GC12	0	96.5726	3.4238	0.0026	0.0001	0.0001	0.0006	0.0001	n.d.	28	-46.8	-25.9	-15.2	-274.0	-221.0	sI	b)
39	Kedr	VER15-03	2015St1GC12	0	96.5030	3.4924	0.0034	0.0002	0.0001	0.0009	0.0001	n.d.	28	-46.5	-25.7	-16.1	-274.1	-223.1	sI	b)
39	Kedr	VER15-03	2015St1GC12	0	95.1310	4.8651	0.0019	0.0001	0.0001	0.0018	n.d.	n.d.	20	-44.9	-25.8	-14.1	-274.5	-223.9	sI	b)
39	Kedr	VER15-03	2015St1GC12	45	86.5303	13.4530	0.0066	0.0003	0.0001	0.0097	n.d.	n.d.	6	-46.5	-26.2	-6.2	-274.9	-226.0	sII	b)
39	Kedr	VER15-03	2015St1GC12	45	86.2522	13.7314	0.0063	0.0003	0.0001	0.0097	n.d.	n.d.	6	-46.5	-26.1	-20.2	-275.7	-226.0	sII	b)
39	Kedr	VER15-03	2015St1GC14	155	86.2521	13.7382	0.0045	0.0001	0.0001	0.0050	n.d.	n.d.	6	-46.1	-25.6	-9.6	-276.9	-226.3	sII	b)
39	Kedr	VER15-03	2015St1GC14	166	86.4323	13.5565	0.0055	0.0002	0.0001	0.0054	n.d.	n.d.	6	-46.9	-25.8	-8.3	-277.1	-226.1	sII	b)
39	Kedr	VER15-03	2015St1GC14	176	86.4636	13.5245	0.0059	0.0002	0.0001	0.0058	n.d.	n.d.	6	-46.1	-25.8	-11.1	-275.9	-221.9	sII	b)
39	Kedr	VER15-03	2015St1GC14	180	86.7910	13.1952	0.0059	0.0002	0.0001	0.0076	n.d.	n.d.	7	-46.4	-25.9	-3.9	-276.2	-227.3	sII	b)
39	Kedr	VER15-03	2015St1GC15	256	97.0606	2.9373	0.0018	0.0000	0.0000	0.0003	n.d.	n.d.	33	-47.0	-25.4	-8.7	-275.1	-213.0	sI	b)
39	Kedr	VER15-03	2015St1GC15	295	92.6942	7.2743	0.0230	0.0019	0.0003	0.0062	0.0000	n.d.	13	-46.1	-25.9	-10.3	-276.0	-224.7	sI	b)
39	Kedr	VER15-03	2015St1GC15	306	88.0757	11.7142	0.1772	0.0151	0.0021	0.0155	0.0001	n.d.	7	-45.7	-26.1	-11.0	-275.5	-225.5	sII	b)
39	Kedr	VER15-03	2015St1GC15	325	97.8802	2.1180	0.0016	0.0001	0.0000	0.0001	n.d.	n.d.	46	-47.7	-25.9	-10.2	-277.4	-220.4	sI	b)
39	Kedr	VER15-03	2015St1GC15	330	96.6346	3.3625	0.0024	0.0001	0.0000	0.0003	n.d.	n.d.	29	-47.0	-25.7	-7.8	-275.4	-215.6	sI	b)
39	Kedr	VER15-03	2015St1GC18	254	85.5533	14.4248	0.0096	0.0008	0.0001	0.0113	n.d.	n.d.	6	-46.6	-25.8	-9.6	-278.7	-215.9	sII	b)
39	Kedr	VER15-03	2015St1GC18	260	86.6301	13.3598	0.0064	0.0003	0.0001	0.0033	n.d.	n.d.	6	-46.9	-25.8	-15.1	-278.2	-216.6	sII	b)
39	Kedr	VER15-03	2015St1GC18	263	85.9662	14.0111	0.0118	0.0008	0.0001	0.0099	n.d.	n.d.	6	-47.1	-25.7	-7.3	-279.4	-221.1	sII	b)
39	Kedr	VER15-03	2015St1GC19	1	97.7360	2.2626	0.0012	0.0000	0.0000	0.0002	n.d.	n.d.	43	-47.5	-25.7	-10.1	-274.7	-210.1	sI	b)
39	Kedr	VER15-03	2015St1GC19	20	97.3698	2.6278	0.0020	0.0000	0.0000	0.0003	n.d.	n.d.	37	-47.6	-25.7	-11.6	-274.3	-211.1	sI	b)
39	Kedr	VER15-03	2015St1GC19	30	97.0742	2.9247	0.0010	0.0000	0.0000	0.0001	n.d.	n.d.	33	-47.8	-25.3	-20.1	-275.4	-209.8	sI	b)
39	Kedr	VER16-03	2016St18GC1	60	94.7068	5.2627	0.0198	0.0022	0.0006	0.0080	0.0000	n.d.	18	-45.8	-26.7	-8.5	-274.0	-227.4	sI	b)
39	Kedr	VER16-03	2016St18GC1	60	93.9883	5.9663	0.0314	0.0030	0.0009	0.0099	0.0001	n.d.	16	-45.6	-26.9	-9.5	-274.1	-224.0	sI	b)
39	Kedr	VER16-03	2016St18GC1	70	96.2253	3.7685	0.0039	0.0004	0.0001	0.0018	0.0000	n.d.	26	-46.0	-26.8	-14.2	-273.4	-222.8	sI	b)
39	Kedr	VER16-03	2016St18GC1	72	96.5722	3.4238	0.0033	0.0003	0.0001	0.0003	0.0000	n.d.	28	-45.9	-27.0	-10.1	-274.1	-216.9	sI	b)
39	Kedr	VER16-03	2016St18GC1	80	96.2361	3.7572	0.0049	0.0005	0.0001	0.0011	0.0001	n.d.	26	-46.2	-26.8	-12.4	-274.1	-219.0	sI	b)
39	Kedr	VER16-03	2016St18GC1	93	97.2146	2.7845	0.0009	0.0000	0.0001	n.d.	n.d.	n.d.	35	-46.3	-26.7	-12.9	-274.2	-214.5	sI	b)
39	Kedr	VER16-03	2016St18GC2	35	96.2169	3.7748	0.0055	0.0005	0.0001	0.0022	0.0000	n.d.	25	-46.8	-26.3	-13.2	-276.4	-207.0	sI	b)
39	Kedr	VER16-03	2016St18GC2	45	97.0115	2.9866	0.0017	0.0001	0.0000	0.0001	0.0000	n.d.	32	-46.5	-26.7	-10.0	-272.8	-205.5	sI	b)
39	Kedr	VER16-03	2016St18GC2	122	85.7577	14.0539	0.1592	0.0120	0.0020	0.0148	0.0003	n.d.	6	-46.0	-27.0	-10.5	-274.7	-215.4	sII	b)
39	Kedr	VER16-03	2016St18GC2	157	85.4582	14.2245	0.2810	0.0114	0.0009	0.0239	0.0001	n.d.	6	-47.1	-26.9	-9.0	-275.5	-220.1	sII	b)
39	Kedr	VER16-03	2016St18GC2	258	96.3457	3.6515	0.0023	0.0001	0.0001	0.0003	n.d.	n.d.	26	-46.5	-26.8	-11.3	-275.4	-208.7	sI	b)

Table S2 (continue)

No.	Site	Cruise	Core	Sample Depth [cmblf]	Molecular Composition: CH_4 [%]	C_2H_6 [%]	C_3H_8 [%]	i-C_4H_{10} [%]	n-C_4H_{10} [%]	neo-C_5H_{12} [%]	i-C_5H_{12} [%]	n-C_5H_{12} [%]	$C_1/(C_2+C_3)$	Isotopic Composition: CH_4 $\delta^{13}C$ [‰VPDB]	C_2H_6 $\delta^{13}C$ [‰VPDB]	C_3H_8 $\delta^{13}C$ [‰VPDB]	CH_4 δ^2H [‰VSMOW]	C_2H_6 δ^2H [‰VSMOW]	Crystallographic Structure	note
39	Kedr	VER16-03	2016St18GC2	269	97.8945	2.1033	0.0018	0.0000	0.0000	0.0003	0.0000	n.d.	47	-46.7	-26.9	-12.0	-273.0	-208.6	sI	b)
39	Kedr	VER16-03	2016St18GC2	277	96.9408	3.0580	0.0009	0.0000	0.0000	0.0001	0.0000	n.d.	32	-46.5	-26.9	-9.0	-276.8	-206.0	sI	b)
39	Kedr	VER16-03	2016St18GC2	305	95.9572	4.0385	0.0030	0.0002	0.0001	0.0010	0.0000	n.d.	24	-46.2	-26.8	-11.8	-276.4	-207.8	sI	b)
39	Kedr	VER16-03	2016St18GC2	333	97.4304	2.5689	0.0006	n.d.	0.0001	n.d.	n.d.	n.d.	38	-46.6	-27.0	-12.3	-273.8	-207.5	sI	b)
39	Kedr	VER16-03	2016St18GC3	233	85.9564	13.9862	0.0371	0.0031	0.0004	0.0167	0.0001	n.d.	6	-46.2	-26.7	-10.8	-278.0	-209.8	sII	b)
39	Kedr	VER16-03	2016St18GC3	242	85.0107	14.8876	0.0718	0.0060	0.0008	0.0230	0.0002	n.d.	6	-46.3	-27.0	-10.1	-278.5	-209.7	sII	b)
39	Kedr	VER16-03	2016St18GC3	255	84.7520	15.0873	0.1187	0.0099	0.0013	0.0305	0.0003	n.d.	6	-46.1	-27.0	-10.2	-280.5	-209.9	sII	b)
39	Kedr	VER16-03	2016St18GC3	262	85.4060	14.5016	0.0583	0.0048	0.0007	0.0284	0.0001	n.d.	6	-46.1	-26.9	-9.8	-278.9	-208.4	sII	b)
39	Kedr	VER16-03	2016St18GC3	275	84.4123	15.4695	0.0782	0.0065	0.0010	0.0323	0.0001	n.d.	5	-46.2	-26.8	-10.6	-278.8	-209.4	sII	b)
39	Kedr	VER16-03	2016St18GC3	287	85.0138	14.8754	0.0747	0.0060	0.0010	0.0291	0.0001	n.d.	6	-46.2	-26.9	-10.3	-278.6	-209.4	sII	b)
39	Kedr	VER16-03	2016St18GC3	312	85.2076	14.5705	0.1702	0.0145	0.0018	0.0350	0.0003	n.d.	6	-46.4	-27.0	-11.1	-279.2	-211.9	sII	b)
39	Kedr	VER16-03	2016St18GC4	159	86.4821	13.4975	0.0118	0.0007	0.0002	0.0078	0.0000	n.d.	6	-46.3	-26.9	-12.0	-278.3	-210.3	sII	b)
39	Kedr	VER16-03	2016St18GC4	170	86.8558	13.0922	0.0347	0.0023	0.0003	0.0146	0.0001	n.d.	7	-46.3	-26.8	-10.8	-276.7	-212.1	sII	b)
39	Kedr	VER16-03	2016St18GC4	174	87.8659	12.1006	0.0197	0.0015	0.0003	0.0120	0.0000	n.d.	7	-46.4	-26.9	-11.5	-274.8	-209.7	sII	b)
39	Kedr	VER16-03	2016St18GC4	177	90.5533	9.4283	0.0108	0.0007	0.0002	0.0067	0.0000	n.d.	10	-46.4	-26.9	-14.1	-275.8	-210.7	sI	b)
39	Kedr	VER16-03	2016St18GC5	3	95.8648	4.1280	0.0047	0.0005	0.0002	0.0018	0.0001	n.d.	23	-45.3	-26.8	-10.1	-276.3	-207.4	sI	b)
39	Kedr	VER16-03	2016St18GC5	20	95.8606	4.1347	0.0033	0.0003	0.0002	0.0009	0.0000	n.d.	23	-45.4	-26.9	-14.6	-275.8	-209.1	sI	b)
39	Kedr	VER16-03	2016St18GC5	23	96.0458	3.9429	0.0080	0.0007	0.0002	0.0023	0.0001	n.d.	24	-45.9	-26.8	-10.6	-276.1	-208.3	sI	b)
39	Kedr	VER16-03	2016St18GC6	205	85.7667	14.2036	0.0167	0.0011	0.0003	0.0116	0.0000	n.d.	6	-46.1	-26.8	-9.6	-276.2	-211.6	sII	b)
39	Kedr	VER16-03	2016St18GC6	210	85.9409	14.0421	0.0081	0.0005	0.0002	0.0083	n.d.	n.d.	6	-46.0	-26.6	-5.4	-277.2	-210.6	sII	b)
39	Kedr	VER16-03	2016St18GC6	230	85.4890	14.3860	0.0980	0.0084	0.0013	0.0171	0.0001	n.d.	6	-46.1	-26.7	-12.3	-276.1	-210.9	sII	b)
39	Kedr	VER16-03	2016St18GC6	253	86.0023	13.9256	0.0509	0.0043	0.0006	0.0163	0.0001	n.d.	6	-46.0	-26.8	-9.4	-276.1	-211.0	sII	b)
39	Kedr	VER16-03	2016St18GC6	272	90.9728	8.9572	0.0481	0.0043	0.0007	0.0167	0.0001	n.d.	10	-45.9	-26.9	-10.3	-276.7	-211.3	sI	b)
39	Kedr	VER16-03	2016St18GC6	293	96.1119	3.8849	0.0027	0.0001	0.0001	0.0004	n.d.	n.d.	25	-45.8	-27.1	-8.8	-275.8	-209.9	sI	b)
39	Kedr	VER16-03	2016St18GC7	149	86.0408	13.8229	0.1024	0.0091	0.0011	0.0236	0.0002	n.d.	6	-46.5	-26.6	-10.8	-276.9	-212.1	sII	b)
39	Kedr	VER16-03	2016St18GC7	159	96.5651	3.4331	0.0014	0.0000	0.0001	0.0002	0.0000	n.d.	28	-46.3	-27.0	-8.0	-276.5	-206.8	sI	b)
39	Kedr	VER16-03	2016St18GC7	173	96.2230	3.7764	0.0003	0.0000	0.0001	0.0001	0.0000	n.d.	25	-46.1	-27.1	-40.2	-275.8	-207.5	sI	b)
39	Kedr	VER16-03	2016St18GC7	184	95.0863	4.8785	0.0252	0.0028	0.0003	0.0069	0.0000	n.d.	19	-46.3	-26.8	-11.5	-277.1	-209.2	sI	b)
39	Kedr	VER16-03	2016St18GC7	194	95.5244	4.4683	0.0052	0.0006	0.0002	0.0013	0.0000	n.d.	21	-46.9	-26.7	-7.9	-277.8	-207.7	sI	b)
39	Kedr	VER16-03	2016St18GC7	194	95.2979	4.6916	0.0073	0.0008	0.0002	0.0022	0.0000	n.d.	20	-47.1	-26.9	-9.9	-276.9	-207.8	sI	b)
39	Kedr	VER17-03	2017St12GC2	142	95.6821	4.2941	0.0150	0.0010	0.0001	0.0077	0.0000	n.d.	22	-47.5	-27.2	-6.6	-275.1	-209.4	sI	b)
39	Kedr	VER17-03	2017St12GC2	160	96.0669	3.9264	0.0055	0.0003	0.0001	0.0009	0.0000	n.d.	24	-47.1	-27.5	-6.2	-273.5	-209.7	sI	b)
39	Kedr	VER17-03	2017St12GC2	173	95.8277	4.1524	0.0140	0.0009	0.0001	0.0048	0.0000	n.d.	23	-47.6	-27.2	-5.6	-274.8	-209.6	sI	b)
39	Kedr	VER17-03	2017St12GC2	184	97.0408	2.9581	0.0009	0.0000	0.0000	0.0001	n.d.	n.d.	33	-47.6	-27.8	n.d.	-274.5	-211.0	sI	b)
39	Kedr	VER17-03	2017St12GC2	197	96.5592	3.4375	0.0029	0.0001	0.0001	0.0002	n.d.	n.d.	28	-47.7	-27.4	-2.4	-273.6	-209.3	sI	b)
39	Kedr	VER17-03	2017St12GC2	198	96.2346	3.7604	0.0042	0.0002	0.0000	0.0006	0.0000	n.d.	26	-47.4	-27.5	-2.9	-274.2	-208.9	sI	b)
39	Kedr	VER17-03	2017St12GC3	266	85.1822	14.7917	0.0131	0.0008	0.0001	0.0121	0.0000	n.d.	6	-47.2	-27.5	-3.6	-275.3	-212.0	sII	b)
39	Kedr	VER17-03	2017St12GC3	293	86.1243	13.8556	0.0120	0.0009	0.0002	0.0070	0.0000	n.d.	6	-47.1	-27.6	-3.4	-273.5	-212.4	sII	b)
39	Kedr	VER19-03	2019St66GC1	217	86.5419	13.4419	0.0094	0.0003	0.0001	0.0064	n.d.	n.d.	6	-46.0	-26.0	n.d.	-278.9	-211.1	sII	
39	Kedr	VER19-03	2019St66GC1	217	86.7513	13.2325	0.0094	0.0003	0.0001	0.0063	n.d.	n.d.	7	-46.5	-26.5	n.d.	-278.6	-211.2	sII	
39	Kedr	VER19-03	2019St66GC4	168	85.5620	14.4207	0.0067	0.0002	0.0001	0.0104	n.d.	n.d.	6	-45.8	-26.3	n.d.	-276.9	-213.6	sII	
39	Kedr	VER19-03	2019St66GC4	198	84.8101	15.0282	0.1232	0.0103	0.0017	0.0261	0.0003	n.d.	6	-46.2	-26.6	n.d.	-277.9	-213.9	sII	
39	Kedr	VER19-03	2019St66GC4	220	86.0524	13.9324	0.0079	0.0003	0.0001	0.0068	n.d.	n.d.	6	-45.7	-26.0	n.d.	-277.4	-212.6	sII	
39	Kedr	VER19-03	2019St67GC1	171	84.5096	15.4501	0.0241	0.0022	0.0005	0.0135	0.0001	n.d.	5	-46.2	-26.1	n.d.	-278.7	-211.8	sII	

Table S2 (continue)

No.	Site	Cruise	Core	Sample Depth [cmblf]	Molecular Composition CH_4 [%]	C_2H_6 [%]	C_3H_8 [%]	$i\text{-}C_4H_{10}$ [%]	$n\text{-}C_4H_{10}$ [%]	$neo\text{-}C_5H_{12}$ [%]	$i\text{-}C_5H_{12}$ [%]	$n\text{-}C_5H_{12}$ [%]	$C_1/(C_2+C_3)$	Isotopic Composition CH_4 $\delta^{13}C$ [‰VPDB]	C_2H_6 $\delta^{13}C$ [‰VPDB]	C_3H_8 $\delta^{13}C$ [‰VPDB]	CH_4 δ^2H [‰VSMOW]	C_2H_6 δ^2H [‰VSMOW]	Crystallographic Structure	note
39	Kedr	VER19-03	2019St67GC1	220	86.5346	13.4463	0.0120	0.0006	0.0002	0.0064	n.d.	n.d.	6	-46.8	-26.5	n.d.	-278.8	-212.3	sII	
39	Kedr	VER19-03	2019St67GC1	223	87.3452	12.6373	0.0103	0.0005	0.0001	0.0066	n.d.	n.d.	7	-46.0	-25.8	n.d.	-277.8	-213.4	sII	
39	Kedr	VER19-03	2019St67GC1	223	87.3186	12.6634	0.0104	0.0005	0.0001	0.0069	0.0000	n.d.	7	-46.4	-26.2	n.d.	-276.4	-212.6	sII	
40	PosolBank-2	VER16-03	2016St1GC2	213	99.8043	0.1936	0.0018	0.0002	0.0000	n.d.	n.d.	n.d.	511	-67.2	-33.4	-23.2	-303.6	-204.4	sI	
40	PosolBank-2	VER16-03	2016St1GC2	218	99.8772	0.1181	0.0044	0.0002	0.0002	n.d.	n.d.	n.d.	816	-67.2	-33.6	-19.4	-303.6	-208.1	sI	
40	PosolBank-2	VER16-03	2016St1GC2	223	99.8629	0.1330	0.0037	0.0002	0.0001	n.d.	n.d.	n.d.	730	-67.1	-33.4	-19.0	-304.1	-208.6	sI	
40	PosolBank-2	VER16-03	2016St1GC2	227	99.8615	0.1344	0.0038	0.0002	0.0001	n.d.	n.d.	n.d.	723	-67.2	-33.5	-17.9	-304.0	-208.3	sI	
40	PosolBank-2	VER16-03	2016St1GC3	240	99.8352	0.1616	0.0029	0.0002	0.0001	n.d.	n.d.	n.d.	607	-67.1	-32.8	-18.6	-304.1	-203.6	sI	
40	PosolBank-2	VER16-03	2016St1GC3	240	99.8353	0.1613	0.0031	0.0003	0.0001	n.d.	n.d.	n.d.	607	-67.2	-33.0	-19.1	-304.4	-206.1	sI	
40	PosolBank-2	VER16-03	2016St1GC7	46	99.8112	0.1858	0.0018	0.0009	0.0003	n.d.	n.d.	n.d.	532	-67.0	-32.7	-23.1	-305.0	-206.8	sI	
40	PosolBank-2	VER16-03	2016St1GC7	54	99.8350	0.1587	0.0048	0.0012	0.0004	n.d.	n.d.	n.d.	611	-66.9	-32.5	-21.9	-303.1	-207.7	sI	
40	PosolBank-2	VER16-03	2016St1GC7	54	99.8615	0.1329	0.0037	0.0016	0.0003	n.d.	n.d.	n.d.	731	-66.8	-32.8	-23.2	-303.7	-208.8	sI	
40	PosolBank-2	VER16-03	2016St1GC7	69	99.8646	0.1333	0.0015	0.0005	0.0001	n.d.	n.d.	n.d.	741	-67.1	-33.1	-24.7	-303.1	-208.0	sI	
40	PosolBank-2	VER16-03	2016St1GC7	83	99.8305	0.1666	0.0018	0.0008	0.0003	n.d.	n.d.	n.d.	593	-66.7	-31.6	-20.8	-302.5	-206.2	sI	
40	PosolBank-2	VER16-03	2016St1GC8	100	99.8836	0.1127	0.0032	0.0003	0.0002	n.d.	n.d.	n.d.	862	-66.6	-33.2	-19.4	-304.4	-204.6	sI	
40	PosolBank-2	VER16-03	2016St1GC8	120	99.8408	0.1567	0.0019	0.0005	0.0002	n.d.	n.d.	n.d.	630	-67.2	-33.5	-20.9	-305.4	-203.6	sI	
40	PosolBank-2	VER16-03	2016St1GC8	133	99.8506	0.1459	0.0031	0.0002	0.0001	n.d.	n.d.	n.d.	670	-67.1	-33.2	-18.0	-304.5	-206.2	sI	
40	PosolBank-2	VER16-03	2016St1GC11	180	99.8077	0.1829	0.0058	0.0029	0.0007	n.d.	n.d.	n.d.	529	-67.8	-32.7	-23.3	-305.1	-220.2	sI	
40	PosolBank-2	VER16-03	2016St1GC11	190	99.8484	0.1487	0.0027	0.0001	0.0001	n.d.	n.d.	n.d.	659	-67.3	-32.9	-19.2	-304.6	-213.3	sI	
40	PosolBank-2	VER16-03	2016St1GC11	200	99.8485	0.1483	0.0030	0.0001	0.0000	n.d.	n.d.	n.d.	660	-67.5	-32.8	-18.0	-304.5	-210.1	sI	
40	PosolBank-2	VER16-03	2016St1GC11	202	99.8629	0.1340	0.0029	0.0001	0.0000	n.d.	n.d.	n.d.	729	-67.6	-32.8	-17.4	-305.0	-205.5	sI	
41	Ostrov	VER16-03	2016St3GC2	22	99.9764	0.0235	0.0001	n.d.	0.0000	n.d.	n.d.	n.d.	4240	-67.2	-64.0	-37.7	-306.4	n.d.	sI	
41	Ostrov	VER16-03	2016St3GC2	25	99.9834	0.0164	0.0002	0.0000	n.d.	n.d.	n.d.	n.d.	6010	-67.4	-63.8	-36.2	-305.0	n.d.	sI	
41	Ostrov	VER16-03	2016St3GC2	44	99.9850	0.0146	0.0003	0.0000	0.0000	n.d.	n.d.	n.d.	6695	-67.1	-63.5	-37.7	-304.9	n.d.	sI	
41	Ostrov	VER16-03	2016St3GC2	67	99.9844	0.0154	0.0002	0.0000	0.0000	n.d.	n.d.	n.d.	6408	-67.3	-63.6	-38.7	-304.5	n.d.	sI	
41	Ostrov	VER16-03	2016St3GC2	102	99.9893	0.0102	0.0004	0.0000	0.0000	n.d.	n.d.	n.d.	9431	-67.0	-63.6	-34.8	-302.8	n.d.	sI	
42	Turka	VER16-03	2016St5GC2	10	99.9465	0.0533	0.0002	n.d.	0.0000	n.d.	n.d.	n.d.	1869	-66.9	-55.8	-33.9	-307.1	-243.3	sI	
42	Turka	VER16-03	2016St5GC2	50	99.9609	0.0388	0.0002	n.d.	0.0000	n.d.	n.d.	n.d.	2560	-66.6	-55.7	-41.0	-306.0	n.d.	sI	
42	Turka	VER16-03	2016St5GC2	65	99.9719	0.0276	0.0005	n.d.	0.0000	n.d.	n.d.	n.d.	3563	-66.8	-55.5	-36.3	-304.9	n.d.	sI	
42	Turka	VER16-03	2016St5GC2	103	99.9620	0.0378	0.0002	n.d.	0.0000	n.d.	n.d.	n.d.	2632	-66.8	-55.6	-33.7	-306.2	n.d.	sI	
42	Turka	VER16-03	2016St5GC2	106	99.9613	0.0385	0.0002	n.d.	0.0000	n.d.	n.d.	n.d.	2584	-66.2	-55.3	-34.7	-306.1	n.d.	sI	
42	Turka	VER16-03	2016St5GC2	112	99.9383	0.0615	0.0002	0.0000	0.0000	n.d.	n.d.	n.d.	1620	-66.6	-55.8	-41.9	-307.8	-241.3	sI	
42	Turka	VER16-03	2016St5GC2	112	99.9360	0.0639	0.0001	n.d.	0.0000	n.d.	n.d.	n.d.	1562	-67.1	-55.9	-25.7	-307.6	-245.1	sI	
42	Turka	VER16-03	2016St5GC3	181	99.9429	0.0569	0.0002	n.d.	0.0000	n.d.	n.d.	n.d.	1750	-66.7	-56.6	-29.3	-308.6	-247.2	sI	
42	Turka	VER16-03	2016St5GC3	195	99.9459	0.0539	0.0002	n.d.	n.d.	n.d.	n.d.	n.d.	1847	-66.9	-56.5	-34.5	-308.3	-246.6	sI	
42	Turka	VER16-03	2016St5GC3	196	99.9511	0.0487	0.0002	n.d.	0.0000	n.d.	n.d.	n.d.	2044	-66.7	-56.6	-42.3	-308.1	-243.3	sI	
43	Kedr-2	VER16-03	2016St19GC1	149	86.9349	13.0515	0.0088	0.0005	0.0002	0.0040	n.d.	n.d.	7	-44.7	-26.8	-11.5	-277.0	-208.7	sII	b)
43	Kedr-2	VER16-03	2016St19GC1	158	87.1356	12.8516	0.0085	0.0003	0.0001	0.0039	n.d.	n.d.	7	-44.7	-27.0	-13.7	-276.3	-210.1	sII	b)
43	Kedr-2	VER16-03	2016St19GC1	162	86.5556	13.4284	0.0106	0.0005	0.0002	0.0047	n.d.	n.d.	6	-44.7	-26.9	-14.1	-277.0	-208.5	sII	b)
43	Kedr-2	VER16-03	2016St19GC1	175	86.8662	13.1142	0.0120	0.0006	0.0001	0.0069	n.d.	n.d.	7	-44.5	-27.0	-10.5	-276.6	-210.4	sII	b)

Table S2 (continue)

No.	Site	Cruise	Core	Sample Depth [cmblf]	CH_4 [%]	C_2H_6 [%]	C_3H_8 [%]	$i\text{-}C_4H_{10}$ [%]	$n\text{-}C_4H_{10}$ [%]	$neo\text{-}C_5H_{12}$ [%]	$i\text{-}C_5H_{12}$ [%]	$n\text{-}C_5H_{12}$ [%]	$C_1/(C_2+C_3)$	CH_4 $\delta^{13}C$ [‰VPDB]	C_2H_6 $\delta^{13}C$ [‰VPDB]	C_3H_8 $\delta^{13}C$ [‰VPDB]	CH_4 δ^2H [‰VSMOW]	C_2H_6 δ^2H [‰VSMOW]	Crystallographic Structure	note
					Molecular Composition									Isotopic Composition						
43	Kedr-2	VER16-03	2016St19GC1	177	87.0761	12.9044	0.0120	0.0007	0.0001	0.0067	n.d.	n.d.	7	-44.4	-26.9	-11.4	-276.9	-209.6	sII	b)
43	Kedr-2	VER17-03	2017St13GC3	181	87.0554	12.9289	0.0092	0.0005	0.0001	0.0059	0.0000	n.d.	7	-44.0	-27.6	-7.6	-275.0	-216.8	sII	b)
43	Kedr-2	VER17-03	2017St13GC3	216	86.4017	13.5547	0.0264	0.0027	0.0002	0.0143	0.0000	n.d.	6	-44.0	-27.6	-9.2	-277.2	-211.6	sII	b)
43	Kedr-2	VER17-03	2017St13GC3	223	85.8880	14.0586	0.0349	0.0030	0.0004	0.0151	0.0000	n.d.	6	-44.2	-27.6	-10.0	-278.1	-211.1	sII	b)
43	Kedr-2	VER17-03	2017St13GC3	232	86.5250	13.4431	0.0200	0.0019	0.0001	0.0098	n.d.	n.d.	6	-44.1	-27.5	-12.6	-275.5	-211.8	sII	b)
43	Kedr-2	VER17-03	2017St13GC3	246	94.9870	4.9987	0.0112	0.0008	0.0003	0.0019	0.0001	n.d.	19	-44.4	-27.7	-11.0	-274.9	-202.7	sI	b)
43	Kedr-2	VER17-03	2017St13GC3	253	94.2214	5.7328	0.0297	0.0039	0.0002	0.0119	0.0000	n.d.	16	-44.5	-27.5	-11.6	-277.4	-204.8	sI	b)
43	Kedr-2	VER17-03	2017St13GC3	255	95.7819	4.2136	0.0035	0.0002	0.0001	0.0007	0.0000	n.d.	23	-44.6	-27.4	-10.9	-276.4	-208.8	sI	b)
44	Solzan	VER16-03	2016St37GC1	190	99.8770	0.1228	0.0001	n.d.	0.0000	n.d.	n.d.	n.d.	812	-70.0	-69.4	-42.2	-293.9	-271.7	sI	
44	Solzan	VER16-03	2016St37GC1	197	99.8695	0.1303	0.0001	n.d.	0.0001	n.d.	n.d.	n.d.	766	-68.7	-67.9	-47.9	-294.6	-278.6	sI	
44	Solzan	VER16-03	2016St37GC6	210	99.8995	0.1004	0.0000	n.d.	0.0001	n.d.	n.d.	n.d.	995	-70.1	-68.9	-33.2	-294.5	-244.2	sI	
44	Solzan	VER16-03	2016St37GC6	210	99.8983	0.1017	0.0000	n.d.	0.0000	n.d.	n.d.	n.d.	982	-70.1	-68.7	-3.7	-295.4	-245.4	sI	
45	Oblom	VER17-03	2017St4GC1	187	99.9602	0.0396	0.0002	0.0000	0.0000	n.d.	n.d.	n.d.	2511	-66.0	-35.5	-29.9	-322.0	-244.6	sI	
45	Oblom	VER17-03	2017St4GC1	187	99.9608	0.0389	0.0002	0.0000	0.0000	n.d.	n.d.	n.d.	2553	-66.5	-35.2	-23.4	-320.6	-239.6	sI	
45	Oblom	VER17-03	2017St4GC1	228	n.d.	n.d.	n.d.	n.d.	n.d.	n.d.	n.d.	n.d.	n.d.	-66.3	n.d.	n.d.	-319.7	n.d.	sI	
46	Turka-2	VER17-03	2017St6GC3	319	n.d.	n.d.	n.d.	n.d.	n.d.	n.d.	n.d.	n.d.	n.d.	-67.5	n.d.	n.d.	-305.2	n.d.	sI	
46	Turka-2	VER17-03	2017St6GC5	153	99.9739	0.0260	0.0000	n.d.	0.0000	n.d.	n.d.	n.d.	3839	-67.2	-60.1	n.d.	-304.6	-309.1	sI	
46	Turka-2	VER17-03	2017St6GC5	153	99.9621	0.0377	0.0002	n.d.	0.0000	n.d.	n.d.	n.d.	2640	-67.0	-60.2	-32.8	-304.5	-316.6	sI	
47	Peschanka P-3	VER17-03	2017St7GC1	309	99.9678	0.0320	0.0001	0.0000	0.0000	n.d.	n.d.	n.d.	3112	-67.7	-66.4	n.d.	-306.7	-303.8	sI	
47	Peschanka P-3	VER17-03	2017St7GC1	309	99.9827	0.0167	0.0005	n.d.	0.0000	0.0000	n.d.	n.d.	5802	-67.4	-66.7	-36.4	-306.0	n.d.	sI	
49	Enkhaluk	VER18-03	2018St1GC1	234	99.9523	0.0476	0.0000	n.d.	0.0000	n.d.	n.d.	n.d.	2098	-66.9	-38.5	n.d.	-313.6	-237.0	sI	
49	Enkhaluk	VER18-03	2018St1GC1	206	99.9379	0.0621	0.0000	n.d.	0.0000	n.d.	n.d.	n.d.	1610	-67.0	-38.5	n.d.	-313.8	-237.4	sI	
50	Sukhaya	VER18-03	2018St5GC1	117	99.9738	0.0261	0.0001	0.0000	0.0000	n.d.	n.d.	n.d.	3814	-66.8	-52.8	n.d.	-311.6	-284.4	sI	
50	Sukhaya	VER18-03	2018St5GC1	123	99.9710	0.0289	0.0000	0.0000	0.0000	n.d.	n.d.	n.d.	3452	-66.8	-52.3	n.d.	-311.2	-282.9	sI	
50	Sukhaya	VER18-03	2018St5GC1	127	99.9745	0.0253	0.0001	0.0000	0.0000	n.d.	n.d.	n.d.	3933	-66.9	-52.0	n.d.	-309.3	-282.5	sI	
50	Sukhaya	VER18-03	2018St5GC1	151	99.9767	0.0232	0.0001	0.0000	0.0000	n.d.	n.d.	n.d.	4295	-66.7	-52.1	n.d.	-309.6	-280.5	sI	
50	Sukhaya	VER18-03	2018St5GC1	164	99.9763	0.0237	0.0000	0.0000	0.0000	n.d.	n.d.	n.d.	4224	-67.1	-52.2	n.d.	-309.8	-277.6	sI	
50	Sukhaya	VER18-03	2018St7GC1	96	99.9665	0.0330	0.0005	0.0000	0.0000	n.d.	n.d.	n.d.	2988	-66.5	-52.1	-30.2	-311.1	-283.4	sI	
50	Sukhaya	VER18-03	2018St7GC1	104	99.9572	0.0427	0.0001	0.0000	0.0000	n.d.	n.d.	n.d.	2337	-66.7	-52.5	n.d.	-313.4	-284.6	sI	
50	Sukhaya	VER18-03	2018St7GC1	110	99.9700	0.0295	0.0005	0.0000	0.0000	n.d.	n.d.	n.d.	3340	-67.0	-52.0	-30.4	-310.5	-280.9	sI	
51	KIT (Kitami)	VER18-03	2018St13GC1	117	99.9647	0.0348	0.0004	0.0000	0.0000	n.d.	n.d.	n.d.	2839	-67.3	-59.2	-35.6	-308.6	-286.5	sI	
51	KIT (Kitami)	VER18-03	2018St13GC1	121	99.9707	0.0287	0.0005	0.0000	0.0000	n.d.	n.d.	n.d.	3423	-67.3	-59.1	-36.0	-307.5	-284.7	sI	
51	KIT (Kitami)	VER18-03	2018St13GC1	125	99.9711	0.0282	0.0005	0.0001	0.0001	n.d.	n.d.	n.d.	3475	-67.3	-59.1	-36.3	-307.9	-284.2	sI	
51	KIT (Kitami)	VER18-03	2018St13GC1	128	99.9645	0.0352	0.0002	0.0000	0.0000	n.d.	n.d.	n.d.	2824	-66.8	-58.7	-36.6	-308.5	-286.2	sI	
51	KIT (Kitami)	VER18-03	2018St14GC1	171	99.9764	0.0229	0.0006	n.d.	0.0000	n.d.	n.d.	n.d.	4252	-66.8	-57.9	-37.1	-306.7	-284.3	sI	
51	KIT (Kitami)	VER18-03	2018St15GC1	137	99.9516	0.0483	0.0000	n.d.	0.0001	n.d.	n.d.	n.d.	2068	-67.7	-59.7	n.d.	-311.1	-290.8	sI	
51	KIT (Kitami)	VER18-03	2018St15GC1	146	99.9665	0.0332	0.0002	n.d.	0.0001	n.d.	n.d.	n.d.	2985	-67.2	-59.0	-37.1	-307.6	-290.6	sI	

Table S2 (continue)

No.	Site	Cruise	Core	Sample Depth [cmblf]	Molecular Composition CH_4 [%]	C_2H_6 [%]	C_3H_8 [%]	i-C_4H_{10} [%]	n-C_4H_{10} [%]	neo-C_5H_{12} [%]	i-C_5H_{12} [%]	n-C_5H_{12} [%]	$C_1/(C_2+C_3)$	Isotopic Composition CH_4 $\delta^{13}C$ [‰VPDB]	C_2H_6 $\delta^{13}C$ [‰VPDB]	C_3H_8 $\delta^{13}C$ [‰VPDB]	CH_4 δ^2H [‰VSMOW]	C_2H_6 δ^2H [‰VSMOW]	Crystallographic Structure	note
51	KIT (Kitami)	VER18-03	2018St15GC1	153	99.9804	0.0191	0.0004	n.d.	0.0000	n.d.	n.d.	n.d.	5112	-67.0	-59.7	-33.6	-306.4	-283.6	sI	
51	KIT (Kitami)	VER18-03	2018St15GC1	155	99.9761	0.0235	0.0003	n.d.	0.0000	n.d.	n.d.	n.d.	4197	-67.3	-59.3	-33.0	-306.8	-285.2	sI	
52	LIN	VER18-03	2018St16GC1	25	99.9742	0.0252	0.0003	0.0000	0.0002	n.d.	n.d.	n.d.	3913	n.d.	n.d.	n.d.	-306.1	n.d.	sI	
52	LIN	VER18-03	2018St16GC1	50	99.9715	0.0283	0.0001	n.d.	0.0000	n.d.	n.d.	n.d.	3519	-67.0	-64.8	n.d.	-309.0	-290.8	sI	
52	LIN	VER18-03	2018St16GC1	60	99.9731	0.0262	0.0003	0.0001	0.0004	n.d.	n.d.	n.d.	3778	-66.3	n.d.	n.d.	n.d.	n.d.	sI	
52	LIN	VER18-03	2018St16GC1	80	99.9691	0.0306	0.0002	0.0000	0.0001	n.d.	n.d.	n.d.	3250	-66.6	n.d.	n.d.	-307.5	n.d.	sI	
52	LIN	VER18-03	2018St16GC1	96	99.9682	0.0316	0.0001	n.d.	0.0000	n.d.	n.d.	n.d.	3149	-66.9	-64.9	n.d.	-308.4	-288.0	sI	
52	LIN	VER18-03	2018St16GC2	101	99.9497	0.0502	0.0000	n.d.	0.0000	n.d.	n.d.	n.d.	1988	-66.6	-65.0	n.d.	-309.9	-292.8	sI	
52	LIN	VER18-03	2018St16GC2	122	99.9575	0.0425	0.0000	n.d.	0.0000	n.d.	n.d.	n.d.	2352	-66.9	-64.6	n.d.	-309.8	-290.3	sI	
53	PosolCanyon	VER18-03	2018St69GC1	110	99.9042	0.0954	0.0003	n.d.	0.0000	n.d.	n.d.	n.d.	1044	-66.3	-31.7	-26.5	-318.1	-224.9	sI	
53	PosolCanyon	VER18-03	2018St69GC1	117	99.9355	0.0640	0.0004	n.d.	0.0000	n.d.	n.d.	n.d.	1550	-66.8	-31.5	-26.8	-316.8	-222.9	sI	
53	PosolCanyon	VER18-03	2018St69GC1	124	99.9312	0.0685	0.0003	n.d.	0.0000	n.d.	n.d.	n.d.	1453	-66.7	-31.5	-30.1	-316.8	-223.8	sI	
53	PosolCanyon	VER18-03	2018St69GC5	25	99.9155	0.0838	0.0004	0.0001	0.0002	n.d.	n.d.	n.d.	1187	n.d.	n.d.	n.d.	-313.1	n.d.	sI	
53	PosolCanyon	VER18-03	2018St69GC5	55	99.8936	0.1060	0.0004	n.d.	0.0000	n.d.	n.d.	n.d.	939	-66.2	-31.0	-21.8	-315.2	-223.5	sI	
53	PosolCanyon	VER18-03	2018St69GC5	72	99.9062	0.0933	0.0005	n.d.	0.0000	n.d.	n.d.	n.d.	1065	-66.3	-31.2	-26.4	-316.1	-223.9	sI	
53	PosolCanyon	VER18-03	2018St69GC5	85	99.9502	0.0492	0.0005	n.d.	0.0000	n.d.	n.d.	n.d.	2009	-66.4	-31.9	-27.6	-314.8	-223.3	sI	
53	PosolCanyon	VER18-03	2018St69GC5	98	99.9465	0.0529	0.0006	n.d.	0.0000	n.d.	n.d.	n.d.	1869	-66.2	-31.6	-28.1	-315.5	-221.9	sI	
53	PosolCanyon	VER18-03	2018St69GC5	111	99.9157	0.0837	0.0005	n.d.	0.0001	n.d.	n.d.	n.d.	1186	-66.2	-31.0	-30.1	-315.4	-223.9	sI	
54	PosolCanyon-2	VER18-03	2018St70GC1	125	99.9278	0.0714	0.0006	0.0001	0.0000	n.d.	n.d.	n.d.	1387	-66.4	-31.6	-30.2	-313.2	-225.2	sI	
54	PosolCanyon-2	VER18-03	2018St70GC1	210	99.9380	0.0613	0.0006	0.0000	0.0000	n.d.	n.d.	n.d.	1614	-66.3	-31.7	-30.1	-314.3	-225.1	sI	
54	PosolCanyon-2	VER18-03	2018St70GC1	217	99.9268	0.0726	0.0005	0.0001	0.0000	n.d.	n.d.	n.d.	1366	-66.6	-31.5	-29.5	-312.5	-225.1	sI	
54	PosolCanyon-2	VER18-03	2018St70GC2	106	99.9520	0.0472	0.0007	0.0000	0.0000	n.d.	n.d.	n.d.	2086	-66.0	-31.6	-30.2	-315.3	-224.0	sI	
54	PosolCanyon-2	VER18-03	2018St70GC2	130	99.9486	0.0508	0.0006	n.d.	0.0000	n.d.	n.d.	n.d.	1944	-66.1	-31.6	-31.3	-316.1	-224.6	sI	
54	PosolCanyon-2	VER19-03	2019St47GC1	65	99.9360	0.0632	0.0007	0.0001	0.0000	n.d.	n.d.	n.d.	1564	-66.7	-29.3	-27.7	-314.2	-224.8	sI	
54	PosolCanyon-2	VER19-03	2019St47GC1	85	99.9416	0.0511	0.0020	0.0031	0.0005	0.0003	0.0014	n.d.	1883	-65.6	-28.9	-28.1	-312.8	-217.9	sI	
54	PosolCanyon-2	VER19-03	2019St47GC1	85	99.9485	0.0493	0.0011	0.0007	0.0001	0.0000	0.0003	n.d.	1985	-66.3	-29.3	-28.3	-313.5	-220.7	sI	
54	PosolCanyon-2	VER19-03	2019St47GC1	90	99.9450	0.0540	0.0008	0.0002	0.0001	n.d.	n.d.	n.d.	1825	-66.5	-29.1	-29.0	-314.0	-224.0	sI	
54	PosolCanyon-2	VER19-03	2019St47GC1	107	99.9410	0.0578	0.0009	0.0003	0.0001	n.d.	n.d.	n.d.	1703	-66.0	-29.0	-26.9	-314.3	-222.2	sI	
54	PosolCanyon-2	VER19-03	2019St47GC1	131	99.9595	0.0395	0.0009	0.0001	0.0001	n.d.	n.d.	n.d.	2476	-66.9	-29.2	-26.3	-313.5	-221.1	sI	
55	Zelen	VER19-03	2019St4GC1	118	99.9907	0.0088	0.0005	0.0000	0.0000	n.d.	n.d.	n.d.	10792	-66.3	-57.1	-24.0	-316.4	n.d.	sI	
55	Zelen	VER19-03	2019St4GC1	143	99.9899	0.0095	0.0004	0.0001	0.0001	n.d.	n.d.	n.d.	10085	-66.6	-56.3	-21.8	-315.9	n.d.	sI	
55	Zelen	VER19-03	2019St4GC1	155	99.9912	0.0081	0.0006	0.0001	0.0000	n.d.	n.d.	n.d.	11468	-66.7	-56.9	-23.5	-316.5	n.d.	sI	
55	Zelen	VER19-03	2019St4GC1	189	99.9892	0.0105	0.0003	0.0000	0.0000	n.d.	n.d.	n.d.	9303	-66.7	-56.4	-23.2	-318.6	n.d.	sI	
55	Zelen	VER19-03	2019St4GC1	238	99.9907	0.0090	0.0002	0.0000	0.0000	n.d.	n.d.	n.d.	10812	-67.2	-56.6	-24.1	-314.4	n.d.	sI	
56	ZelenSeep	VER19-03	2019St6GC1	246	98.6637	1.3261	0.0097	0.0001	0.0001	0.0003	n.d.	n.d.	74	-50.2	-24.5	-11.6	-294.7	-208.4	sI	
56	ZelenSeep	VER19-03	2019St6GC1	250	99.0388	0.9520	0.0084	0.0001	0.0001	0.0006	n.d.	n.d.	103	-49.6	-24.7	-10.6	-294.5	-208.3	sI	
56	ZelenSeep	VER19-03	2019St6GC1	254	99.0806	0.9103	0.0087	0.0000	0.0000	0.0003	n.d.	n.d.	108	-49.4	-24.4	-11.0	-293.2	-207.3	sI	
56	ZelenSeep	VER19-03	2019St6GC1	271	98.9785	1.0123	0.0089	0.0000	0.0001	0.0002	n.d.	n.d.	97	-49.8	-24.6	-10.4	-293.1	-207.5	sI	

Table S2 (continue)

No.	Site	Cruise	Core	Sample Depth [cmblf]	Molecular Composition CH$_4$ [%]	C$_2$H$_6$ [%]	C$_3$H$_8$ [%]	i-C$_4$H$_{10}$ [%]	n-C$_4$H$_{10}$ [%]	neo-C$_5$H$_{12}$ [%]	i-C$_5$H$_{12}$ [%]	n-C$_5$H$_{12}$ [%]	C$_1$/(C$_2$+C$_3$)	Isotopic Composition CH$_4$ δ^{13}C [‰VPDB]	C$_2$H$_6$ δ^{13}C [‰VPDB]	C$_3$H$_8$ δ^{13}C [‰VPDB]	CH$_4$ δ^{2}H [‰VSMOW]	C$_2$H$_6$ δ^{2}H [‰VSMOW]	Crystallographic Structure	note
57	Melky	VER19-03	2019St7GC2	193	99.5283	0.4574	0.0136	0.0004	0.0001	0.0002	n.d.	n.d.	211	-56.7	-25.9	-13.3	-305.2	-209.9	sI	
57	Melky	VER19-03	2019St7GC2	193	99.5338	0.4519	0.0134	0.0004	0.0001	0.0004	n.d.	n.d.	214	-57.1	-26.0	-13.0	-305.7	-210.7	sI	
57	Melky	VER19-03	2019St7GC2	208	99.5715	0.4057	0.0202	0.0011	0.0001	0.0015	n.d.	n.d.	234	-57.1	-25.9	-13.1	-304.9	-208.6	sI	
57	Melky	VER19-03	2019St7GC3	5	99.5690	0.4181	0.0110	0.0002	0.0001	0.0017	n.d.	n.d.	232	-57.0	-25.9	-12.1	-305.6	-210.4	sI	
57	Melky	VER19-03	2019St7GC3	15	99.5175	0.4640	0.0137	0.0005	0.0001	0.0042	n.d.	n.d.	208	-55.9	-25.3	-12.0	-303.7	-208.8	sI	
57	Melky	VER19-03	2019St7GC3	35	99.5391	0.4456	0.0110	0.0003	0.0001	0.0039	0.0001	n.d.	218	-56.5	-25.6	-11.9	-304.7	-209.3	sI	
58	Katko	VER19-03	2019St13GC1	205	99.8290	0.1703	0.0005	0.0001	0.0000	n.d.	n.d.	n.d.	584	-65.6	-53.9	-34.5	-303.3	-294.5	sI	
58	Katko	VER19-03	2019St13GC3	276	99.7920	0.2079	0.0001	0.0000	0.0000	n.d.	n.d.	n.d.	480	-66.3	-53.7	-33.3	-307.4	-295.8	sI	
58	Katko	VER19-03	2019St13GC3	276	99.7796	0.2201	0.0002	0.0001	0.0001	n.d.	n.d.	n.d.	453	-66.2	-53.7	-33.7	-307.9	-294.7	sI	
59	BelKamen	VER19-03	2019St14GC2	121	99.9786	0.0203	0.0010	0.0001	0.0000	n.d.	n.d.	n.d.	4702	-66.7	-57.9	-34.1	-294.4	-268.7	sI	
59	BelKamen	VER19-03	2019St14GC2	136	99.9545	0.0441	0.0012	0.0002	0.0001	n.d.	n.d.	n.d.	2207	-67.0	-58.4	-31.8	-297.7	-280.6	sI	
59	BelKamen	VER19-03	2019St14GC2	152	99.9514	0.0464	0.0017	0.0004	0.0001	n.d.	n.d.	n.d.	2079	-66.6	-58.2	-31.3	-298.2	-279.6	sI	
59	BelKamen	VER19-03	2019St14GC2	155	99.9454	0.0218	0.0247	0.0069	0.0008	0.0001	0.0003	n.d.	2149	-66.0	-58.2	-33.7	-294.7	-269.2	sI	
60	Kukuy K-17	VER19-03	2019St43GC1	85	99.9884	0.0114	0.0002	0.0000	0.0000	n.d.	n.d.	n.d.	8632	-67.4	-46.4	-31.6	-314.1	n.d.	sI	
60	Kukuy K-17	VER19-03	2019St43GC1	85	99.9885	0.0113	0.0002	0.0000	0.0000	n.d.	n.d.	n.d.	8695	-67.3	-46.9	-27.9	-314.8	n.d.	sI	
60	Kukuy K-17	VER19-03	2019St43GC1	86	99.9886	0.0112	0.0002	0.0000	0.0000	n.d.	n.d.	n.d.	8795	-67.2	-46.9	-29.5	-314.8	n.d.	sI	
60	Kukuy K-17	VER19-03	2019St43GC1	86	99.9886	0.0112	0.0002	0.0000	0.0000	n.d.	n.d.	n.d.	8769	-67.3	-46.5	-27.3	-315.1	n.d.	sI	
